# NEDD4L-mediated Gasdermin D and E ubiquitination regulates cell death and tissue injury

**DOI:** 10.1038/s41418-025-01598-1

**Published:** 2025-11-19

**Authors:** Sonia S. Shah, Jantina A. Manning, Yoon Lim, Diva Sinha, Ambika Mosale Venkatesh Murthy, Raja Ganesan, Nirmal Robinson, Emad S. Alnemri, Seth L. Masters, James E. Vince, Sharad Kumar

**Affiliations:** 1 Centre for Cancer Biology, University of South Australia, Adelaide, SA 5001 Australia; 2 Adelaide University, Adelaide, SA 5005 Australia; 3 University Hospital Cologne, TRIO Research Center, 50931 Köln, Germany; 4 Adelaide Medical School, The University of Adelaide, Adelaide, SA 5005 Australia; 5 Department of Biochemistry and Molecular Biology, Sidney Kimmel Cancer Center, Thomas Jefferson University, Philadelphia, PA 19107 USA; 6 The Walter and Eliza Hall Institute of Medical Research, Parkville, VIC 3052 Australia; 7 Centre for Innate Immunity and Infectious Diseases, Hudson Institute of Medical Research, Clayton, VIC 3168 Australia; 8 The Department of Medical Biology, University of Melbourne, Parkville, VIC 3010 Australia

**Keywords:** Proteolysis, Proteolysis

## Abstract

The membrane pore-forming gasdermin (GSDM) proteins are essential executors of pyroptosis. The GSDM family members GSDMD and GSDME can also target mitochondrial membranes, driving apoptosis. Here, we identify the ubiquitin ligase NEDD4L as a key regulator of GSDMD and GSDME, two GSDMs involved in cell death. NEDD4L ubiquitinates both these proteins to control their stability and intracellular expression levels. Knockout of mouse *Nedd4l* (also called *Nedd4-2*) results in lung and kidney damage with perinatal lethality within three weeks of birth. These mice demonstrated elevated GSDMD in alveolar epithelia and increased GSDME in kidney tubular epithelia, suggesting tissue-specific regulation by NEDD4L. Renal tubule-specific *Nedd4l* knockout mice showed GSDM activation, tubular cell death and reduced kidney function after high sodium diet. NEDD4L-deficient cells showed increased GSDM activation, IL-1β release and were significantly more susceptible to cell death induced by NLRP3 agonists, cytotoxic agents, and bacterial infection. These results demonstrate that NEDD4L regulates GSDMD and GSDME functions by preventing their accumulation and reveals an unexplored link between GSDM stability and cell death.

## Introduction

Gasdermins are pore-forming proteins that play an essential role in mediating pyroptosis, a lytic type of inflammatory cell death [1–3]. The Gasdermin (GSDM) protein family in humans has six members GSDMA, GSDMB, GSDMC, GSDMD, GSDME and PJVK/GSDMF that show distinct tissue expression [4]. They play a crucial role in various biological processes such as immune response, and have been associated with cancers, autoimmune diseases and other disorders [5]. All GSDMs contain a pore-forming N-terminal domain (NTD) and a repressor C-terminal domain (CTD) that is separated by a linker region (except PJVK/GSDMF). Several cellular stressors such as bacterial infections, ionic imbalance, and toxins cause proteolytic cleavage at the linker that allows the NTD to oligomerise and form large membrane pores. This results in osmotic swelling, cytokine release, plasma membrane rupture and pyroptosis [4–7]. Recently, some other groups have shown pore formation that occurs independently of GSDM cleavage such as PARylation of GSDME that induces conformational changes to relieve the autoinhibition or palmitoylation of GSDMD at Cys191, which allows the full-length protein to oligomerise and form membrane pores [8, 9]. Moreover, GSDMs have non-lytic roles such as maintaining ion flux, organelle integrity, and protein release [10] with several post-transcriptional and post-translational changes being identified as crucial modifiers of GSDM activity [10–12].

Ubiquitination is a versatile process that involves the covalent attachment of a 8 kD protein, Ubiquitin (Ub) to its substrate in a multistep process mediated by Ub activating enzyme (E1), Ub conjugating enzymes (E2) and Ub-substrate ligases (E3) [13]. Protein ubiquitination not only regulates protein stability but can affect protein sorting, intracellular trafficking and signaling and this is largely determined by the topology of the Ub chains [14, 15]. Several proteins involved in the process of cell death and inflammation such as TNFR1, RIP1, RIP3, Mcl-1, Bcl-xl and NLRP3 are regulated by ubiquitination [16]. GSDMD is polyubiquitinated by SYVN1, a RING E3 ligase and this modification was found to promote cell death [17].

NEDD4L (mouse NEDD4-2; neural precursor cell expressed developmentally downregulated 4-like), is a member of the NEDD4 family of HECT type of E3 that maintains cellular homeostasis by regulating a number of membrane proteins, including ion channels and transporters [18, 19]. The complete deletion of *Nedd4-2* in mice (*Nedd4-2*^−/−^) results in perinatal lethality due severe respiratory distress from collapsed lungs caused by increased epithelial sodium channel (ENaC) activity leading to premature fluid clearance. Around 4% of knockout (KO) animals that survive birth die within three weeks with sterile lung inflammation and kidney damage [20]. *Nedd4-2*^*Ksp1.3*^ mice with kidney tubule-specific deletion of *Nedd4-2* in adult animals have a normal lifespan; however, they develop progressive kidney disease with loss of tubular epithelial cells, immune cell infiltration, and upregulation of sodium ion channels ENaC and sodium chloride co-transporter (NCC) [21]. When fed a high sodium diet (3.1% Na^+^) *Nedd4-2*^*Ksp1.3*^ mice rapidly develop tubular damage, decline in kidney function, increased fibrosis and end-stage kidney disease [22]. This was partly attributed to ionic imbalance caused by increased ENaC activity and Na^+^ reabsorption. Tubular cell death in *Nedd4-2*^*Ksp1.3*^ mice accompanies caspase-3 activation, however precise mechanisms of tubular epithelial loss remain unknown.

In this study, we show that NEDD4L (mouse NEDD4-2) is an essential regulator of the stability of GSDMD and GSDME. We find increased GSDMD and GSDME expression in cells and tissues that lack *NEDD4L* and show that NEDD4L binds and directly ubiquitinates both GSDMD and GSDME. *NEDD4L* KO cells undergo rapid cell death with GSDMD and GSDME activation when treated with NLRP3 agonists or cell death inducers such as etoposide, cisplatin or TNFα/cycloheximide. A high Na^+^ diet induced acute kidney injury in *Nedd4-2*-deficient mice caused GSDMD and GSDME activation, tubular cell death and an inflammatory phenotype in the kidney that led to a significant decline in kidney function. Further, loss of *NEDD4L* promoted cell death in *S*. Typhimurium-infected THP-1 cells that was associated with increased GSDMD and GSDME activation. Our data demonstrates for the first time that ubiquitination by NEDD4L is a crucial regulator of GSDMD and GSDME stability and their functions.

## Results

### Loss of *Nedd4-2* in mice causes Gasdermin D and E activation and cell death

**Fig. 1:**
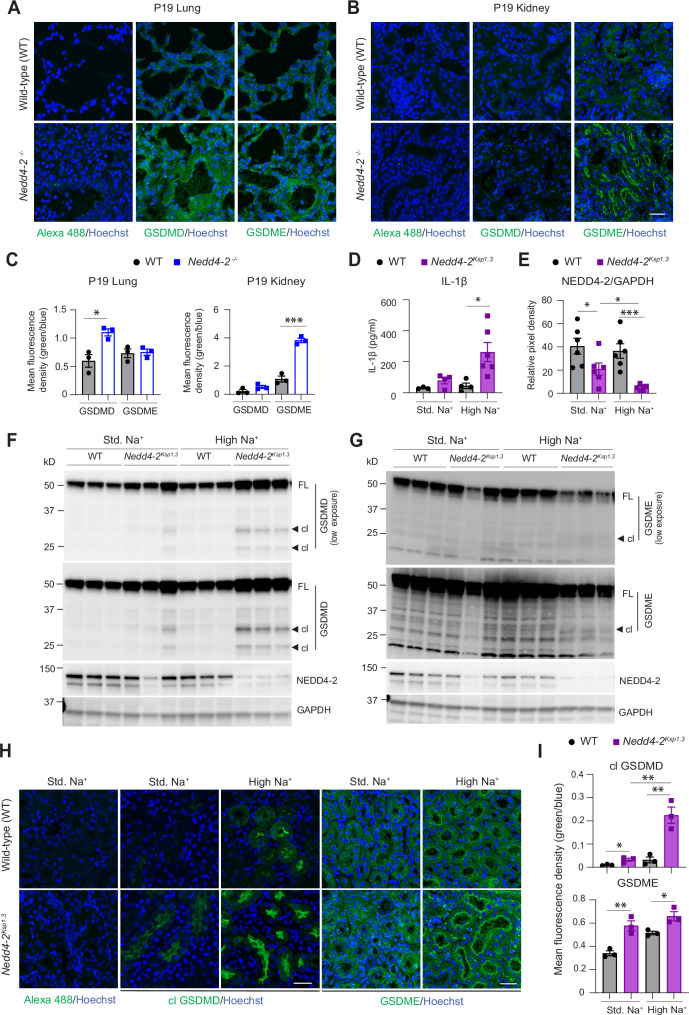
High salt diet causes Gasdermin D and E activation in *Nedd4-2*^*Ksp1.3*^ mouse kidney. Representative images showing immunostaining for GSDMD and GSDME (green) in **A** lungs and **B** kidneys of survivor postnatal day 19 (P19) WT and *Nedd4-2* knockout (*Nedd4-2*^*−/−*^) mice. DNA is stained with Hoechst (blue). Scale bar = 50 μm. **C** Quantitation of GSDMD and GSDME immunostaining using ImageJ. **D** Blood IL-1β levels measured in WT and *Nedd4-2*^*Ksp1.3*^ mice fed standard (Std.) or high Na^+^ diet. **E** Quantitation of NEDD4-2 expression in kidney samples using ImageJ. **F**, **G** Immunoblots showing full-length (FL) and cleaved (cl) forms of GSDMD and GSDME in kidney lysates from WT and *Nedd4-2*^*Ksp1.3*^ mice fed standard (Std.) or high Na^+^ diet. **H** Representative images showing immunostaining for cleaved GSDMD and total GSDME (green) in kidneys from WT and *Nedd4-2*^*Ksp1.3*^ mice fed standard (Std.) or high Na^+^ diet. Scale bar = 50 μm. **I** Quantitation of cleaved GSDMD and total GSDME immunostaining in kidneys from WT and *Nedd4-2*^*Ksp1.3*^ mice fed standard (Std.) or high Na^+^ diet using ImageJ. Immunoblots and confocal images are representative from three independent experiments. Data are Mean ± SEM (*n* = at least 3 mice/group). Statistical analysis was performed using the two-tailed unpaired Student’s *t*-test. *P < 0.05, **P < 0.01 and ***P < 0.001.

As tubular cell death in kidney and loss of alveolar epithelia in the lung of *Nedd4-2* KO animals are accompanied by immune cell infiltration and fibrosis [20, 21] we investigated the possible role of *Nedd4-2* deficiency in inflammatory cell death. While exploring additional NEDD4-2 substrates, we identified GSDMD and GSDME as candidates (Supplementary Fig. S1A) using Ubibrowser 1.0, an integrated database for predicting E3-substrate interactions [23]. Initially, we used lung and kidney sections from surviving P19 *Nedd4-2*^*−/−*^ animals and observed increased levels of GSDMD protein in the lung and GSDME protein in the kidney (Fig. 1A–C). This was associated with a higher incidence of macrophages (F4/80^+^) in the P19 *Nedd4-2*^*−/−*^ mice lung and kidney (Supplementary Fig. S1B, C). GSDMD and GSDME are known to undergo caspase-mediated cleavage at the linker region that results in the release of the pore-forming NTD (caspase cleavage sites summarised in Supplementary Fig. S1D). Adult kidney tubule-specific *Nedd4-2* KO (*Nedd4-2*^*Ksp1.3*^) fed a high salt diet showed elevated IL-1β levels in blood (Fig. 1D). As the caspase-1-GSDMD pathway is involved in the activation and release of mature IL-1β, we assessed proteins involved in this pathway in mice kidneys from variable salt diets. It is important to note that the *Nedd4-2*^*Ksp1.3*^ mice lack NEDD4-2 only in renal tubules [21]. The immunoblots of kidney lysates display about 50% reduction of NEDD4-2 (Fig. 1E) in *Nedd4-2*^*Ksp1.3*^ mice. This is because these mice were generated by crossing the *Nedd4-2*^*fl/fl*^ mouse line to the Ksp1.3-Cre transgenic mice and lack *Nedd4-2* expression in Ksp-cadherin (also known as cadherin-16) expressing cells of the collecting ducts, loop of Henle, proximal and distal tubules. Non-tubular cells (non-cadherin-16 expressing cells) such as glomerulus and blood vessels account for the remaining NEDD4-2 expression [21]. We observed that GSDMD expression was elevated in *Nedd4-2*^*Ksp1.3*^ mice on a standard salt diet (Fig. 1F). The high Na^+^ diet resulted in further decline in NEDD4-2 expression (Fig. 1E) along with activation of both GSDMD and GSDME, and increased cell death indicated by activation of caspase-3 in the *Nedd4-2*^*Ksp1.3*^ mice (Fig. 1F, G, and Supplementary Fig. S1E). Consistently, the high Na^+^ diet in *Nedd4-2*^*Ksp1.3*^ mice promoted increased localisation of cleaved GSDMD and total GSDME protein to apical epithelia of renal tubules (Fig. 1H, I). No changes in TNFα or IL6 levels were observed (Supplementary Fig. S1F). However, NLRP3 and caspase-1 expression was higher in the high Na^+^ fed *Nedd4-2*^*Ksp1.3*^ mice with increased activation of caspase-1 observed in this group suggesting inflammasome activation (Supplementary Fig. S1G). Thus, GSDMD and GSDME mediated cell death likely augments tubular loss and results in tissue injury in *Nedd4-2*^*Ksp1.3*^ mice. As dietary salt can alter kidney transcriptome [24], we performed a qRT-PCR on kidney samples and found that *GsdmE* gene expression remained unaltered however the high Na^+^ diet increased *GsdmD*, *Nlrp3* and *IL-1*β expression in *Nedd4-2*^*Ksp1.3*^ mice (Supplementary Fig. S2A). The loss of tubular cells was accompanied by increased infiltration of macrophages (F4/80^+^) and neutrophils (Ly6G^+^) in the *Nedd4-2*^*Ksp1.3*^ kidneys (Supplementary Fig. S2B–D) that are recruited to the damaged tubules and contribute to inflammation and fibrosis. Overall, our data demonstrate that increased cell death due to GSDMD and GSDME mediated pyroptosis and apoptosis exacerbates salt induced tubular damage in *Nedd4-2*^*Ksp1.3*^ mice.

### NEDD4L regulates Gasdermin D and E

**Fig. 2:**
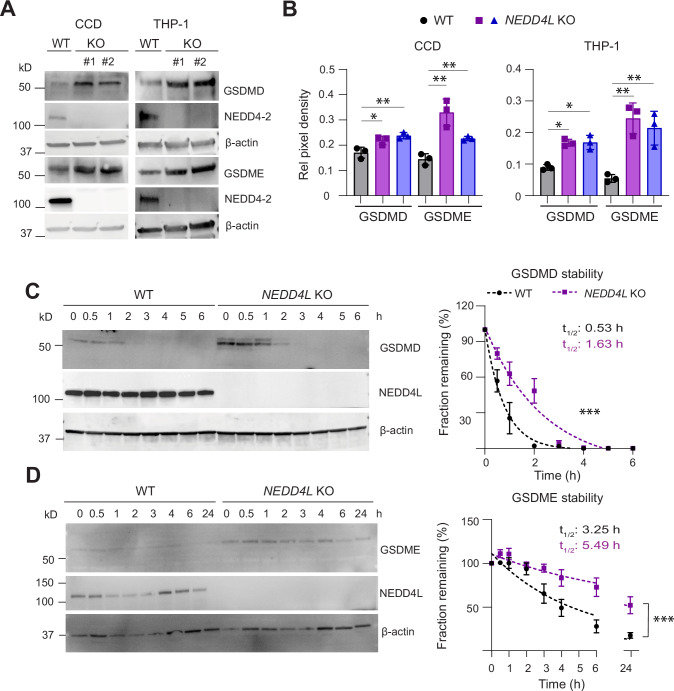
NEDD4L/NEDD4-2 deficiency results in increased Gasdermin D and E expression. **A** Immunoblots from kidney cortical collecting duct (CCD) and THP-1 cells and **B** their quantitation shows higher GSDMD and GSDME expression in *Nedd4-2/NEDD4L* KO cells. #1, #2 are different clones of the *Nedd4-2*/*NEDD4L* KO cells used. **C**, **D** Protein stability was measured by blocking translation in THP-1 cells using cycloheximide (CHX) and harvesting cells at different time points as shown. Data are Mean ± SD from three independent experiments. Statistical analysis was performed using the two-tailed unpaired Student’s *t*-test in (**B**) and nonlinear regression analysis using one-phase decay was used to calculate the half-life (t_1/2_) for GSDMD and GSDME in (**C**, **D**). *P < 0.05, **P < 0.01 and ***P < 0.001.

To determine if the altered GSDMD and GSDME expression in mouse tissues was directly due to the absence of *Nedd4-2*, we used previously generated kidney cortical collecting duct (CCD) cells with CRISPR-mediated *Nedd4-2* deletion [22]. These cells do not express NLRP3 but have high NEDD4-2 expression making them an excellent cell line to study GSDM expression independently of NLRP3. Significantly increased GSDMD and GSDME expression was observed in the *Nedd4-2* KO cells (Fig. 2A, B). Next, using human myeloid THP-1 cells, we generated *NEDD4L* KO THP-1 cells by CRISPR/Cas9. These were validated by immunoblotting for NEDD4L (Supplementary Fig. S3A). Higher GSDMD and GSDME expression was observed in the *NEDD4L* KO THP-1 cells (Fig. 2A, B). *NEDD4L* knockdown in other cell lines, such as A549 and U2OS cells, also showed similar results (Supplementary Fig. S3B–D). To test if the loss of one *Nedd4-2* allele affects GSDM expression and if this causes any kidney injury, high Na^+^ diet was fed to age matched WT and *Nedd4-2* heterozygous (*Nedd4-2*^+/−^) mice for 3 months and animals were monitored by measuring weekly body weight changes, blood pressure (BP) and metabolic parameters such as food and water intake, urination before (Pre) and after (Post) the Na^+^ diet (Supplementary Fig. S4A). Urine and blood samples were analysed for common metabolites and kidney function (glomerular filtration rate, GFR) calculated (Table 1 and Supplementary Fig. S4B). Although no significant changes in systolic BP, and GFR were observed, *Nedd4-2*^+/−^ mice demonstrated elevated expression of kidney injury marker (*Kim*1), polyuria, polydipsia with increased urea and protein levels in urine (Supplementary Fig. S4B and Table 1) suggesting elevated kidney damage or loss of renal function. Furthermore, compared to the WT mice, elevated urinary Na^+^, K^+^, Cl^−^ and Ca^2+^ in *Nedd4-2*^+/−^ mice indicates electrolyte imbalance. Kidney lysates from high Na^+^ diet fed *Nedd4-2*^+/−^ mice also showed elevated GSDMD and GSDME expression and activation of GSDMD (Supplementary Fig. S4C). NLRP3 expression between the control and *Nedd4-2*^+/−^ mice was unchanged. In addition, we isolated bone marrow from these mice and differentiated them into macrophages (BMDMs). A significant increase in GSDMD and GSDME expression and activation in BMDMs obtained from *Nedd4-2*^+/−^ mice was observed (Supplementary Fig. S4D). Thus, GSDMD and GSDME are regulated by NEDD4-2.

**Table 1:** Urine and serum analysis of wild-type (WT) and heterozygous mice (*Nedd4-2*^*+/−*^) fed a high Na^+^ diet for 3 months.

	Wild-type (WT)	Heterozygous (*Nedd4-2*^*+/−*^)
Body weight (g)	21.14 ± 1.16 (5)	21.41 ± 1.02 (10)
GFR (ml/g body weight/24 h)	7.55 ± 1.96 (5)	8.99 ± 2.51 (8)
**Urine**		
Urea (mM)	284.40 ± 104.14 (5)	191.78 ± 91.50 (9)
Creatinine, Cre (mM)	0.78 ± 0.46 (5)	**0.35** ± 0.22 (10)*
Osmolarity (mOsmol/L)	1160.66 ± 334.06 (3)	817.14 ± 162.91 (7)
Na^+^/Cre	458.00 ± 206.02 (5)	**1430.00** ± **811.26** (9) **
K^+^/Cre	79.60 ± 20.20 (5)	**195.77** ± **86.79** (9) ***
Cl^-^/Cre	454.00 ± 208.510 (5)	**1449.11** ± **861.83** (9) **
Ca^2+^/Cre	3.31 ± 0.59 (5)	**8.39** ± **4.94 (10)** **
Pro/Cre	300.40 ± 100.50 (5)	**512.20** ± **109.04 (10)** **
Urea/Cre	390.60 ± 57.32 (5)	**790.11** ± **333.88 (9)** ***
**Serum**		
Na^+^ (mM)	159.00 ± 10.41 (5)	155.40 ± 3.97 (10)
K^+^ (mM)	10.14 ± 1.45 (5)	9.12 ± 1.29 (10)
Cl^-^ (mM)	120.20 ± 8.28 (5)	114.30 ± 5.07.5(10)
Bicarbonate (mM)	20.80 ± 3.42 (5)	22.90 ± 3.60 (10)
Glucose (mM)	14.82 ± 2.11 (5)	12.91 ± 1.78 (10)
Urea (mM)	11.40 ± 4.72 (5)	**6.72** ± **1.34 (10)** **
Creatinine (mM)	9.00 ± 2.44 (5)	**5.87** ± **1.35 (8)** **
Albumin (g/L)	16.20 ± 1.48 (5)	15.80 ± 1.47 (10)
Globulin (g/L)	35.40 ± 2.70 (5)	34.30 ± 3.02 (10)
Total protein	51.60 ± 4.15 (5)	50.10 ± 4.33 (10)

Data is Mean ± SD for a number (*n*) of mice in parentheses. Statistical significance was calculated by the Mann–Whitney test. Bold values are statistically significant.

*P < 0.05, **P < 0.01, ***P < 0.001.

Given that NEDD4L is an E3 ligase that regulates its substrates by ubiquitination, we examined if the stability of GSDMD and GSDME proteins is altered by NEDD4L. Cycloheximide chase experiments were performed in WT and *NEDD4L* KO cells (THP-1 cells for GSDMD and CCD cells for GSDME). Data were analysed by non-linear regression (GraphPad Prism) to measure one-phase decay using a least squares approach. The GSDMD and GSDME decay rates were significantly different between WT and *NEDD4L* KO cells (*p* < 0.0001) resulting in notably longer half-life of both GSDMD and GSDME in the *NEDD4L* KO cells (Fig. 2C, D), thus indicating that NEDD4L controls the turnover of these proteins.

### NEDD4L interacts with GSDMD and GSDME

**Fig. 3:**
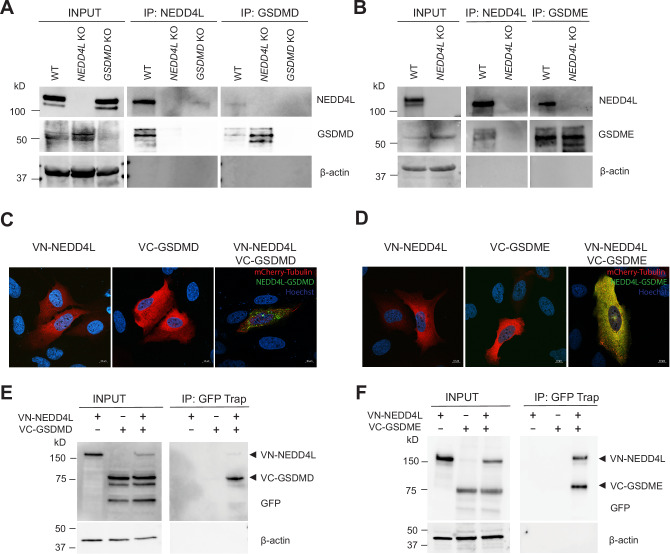
Gasdermin D and E interact with NEDD4L. Immunoprecipitations were performed in THP-1 cells (for GSDMD) and CCD (for GSDME) using the indicated antibodies. Immunoblots showing interaction of endogenous NEDD4L with **A** GSDMD and **B** GSDME in the WT cells. *NEDD4L* and *GSDMD* KO cells were used as controls. INPUT; IP: Immunoprecipitation. **C**, **D** Representative confocal images from a BiFC experiment showing non-fluorescent controls (individually expressed VN-NEDD4L and VC-GSDMD/ VC-GSDME) and green GFP signal when the proteins are co-expressed and interact with each other. mCherry-Tubulin was used as a transfection control. Scale bar = 10 μm. **E**, **F** Immunoblots from IP analysis using GFP Trap beads suggest interaction and formation of NEDD4L-GSDMD/E complex. We show a representative result from three independent experiments.

To examine if NEDD4L interacts with GSDMD and GSDME, wild-type (WT) THP-1 cells were used to perform immunoprecipitation assays using antibodies specific for NEDD4L, GSDMD and GSDME (Fig. 3A, B). *NEDD4L* KO and *GSDMD* KO cells were used as controls. We observed that endogenous NEDD4L interacts with endogenous GSDMD and GSDME proteins (Fig. 3A, B). Bimolecular fluorescence complementation (BiFC) was then used to monitor protein interaction. U2OS cells were co-transfected with VN-NEDD4L and VC-GSDMD or VC-GSDME or either plasmid alone. mCherry-tubulin was used as a transfection control. No BiFC positive fluorescence was observed when either plasmid was expressed alone. However, proximity-induced interaction generated the BiFC positive Venus fluorescence when VN-NEDD4L was co-expressed with either VC-GSDMD or VC-GSDME confirming protein interaction (Fig. 3C, D). Similarly, pull-down assays of the above proteins using GFP-TRAP beads also demonstrated formation of a Venus complex when both the proteins are expressed together, confirming that NEDD4L interacts with GSDMD and GSDME (Fig. 3E, F).

### NEDD4L ubiquitinates GSDMD and GSDME

**Fig. 4:**
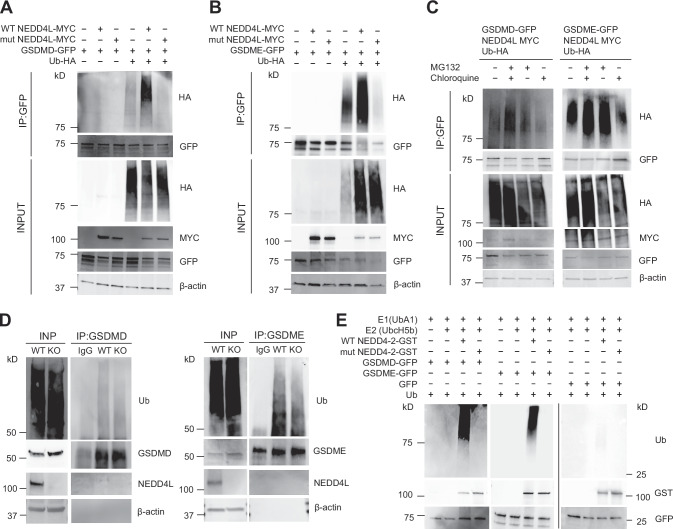
Gasdermin D and E are ubiquitinated by NEDD4L. **A**, **B** Immunoprecipitation (IP) analysis using HEK293T cells co-expressing GSDMD and GSDME with either WT NEDD4L or a catalytically inactive mutant (mut). Anti-GFP immunoprecipitates were analysed by immunoblots using the indicated antibodies and show that WT NEDD4L ubiquitinates GSDMD and GSDME but not the mutant protein. **C** IP analysis was performed after treating transfected cells with either MG132 or chloroquine or both for 4 h prior to cell harvest. Immunoblots show IP of the ubiquitinated proteins. **D** WT and *NEDD4L* KO cells were harvested and used to immunoprecipitate endogenous GSDMD and GSDME. Immunoblots from IPs show *NEDD4L* KO cells display a weaker signal from ubiquitin tracts suggesting reduced ubiquitination of GSDMD and GSDME. Rabbit IgG (serum) was used for non-specific binding (*n* = 3). INP: INPUT; IP: Immunoprecipitation. **E** GST-tagged NEDD4-2 proteins (both WT and a catalytic mutant) were purified. HEK293T cells were transfected with GSDMD-GFP, GSDME-GFP or empty vector (pEGFP-N1). Cells were harvested and GFP-tagged proteins were immobilised on GFP-Trap beads and thereafter eluted. Ubiquitination reactions were set up in vitro using E1, E2, N4-2 (E3), ATP and ubiquitin for 90 min at 37 °C. Reaction was stopped by adding SDS loading buffer and samples were analysed by immunoblotting with desired antibodies. Representative data from three independent experiments is shown.

To examine if NEDD4L ubiquitinates GSDMD and GSDME, we co-expressed GFP-GSDMD/E and MYC-NEDD4L in HEK293T cells. Expression vectors for active WT NEDD4L and a catalytically inactive mutant (Cys942Ala, mut) were used with or without HA-tagged Ubiquitin (Ub-HA). As shown in Fig. 4A, B, both GSDMD and GSDME were ubiquitinated by NEDD4L, and this process was dependent on the presence of the active enzyme since the cells transfected with Cys942Ala NEDD4L displayed significantly reduced ubiquitination. To check how NEDD4L mediated ubiquitination affects GSDMD and GSDME, we transfected HEK293T cells with GFP-GSDMD/E, MYC-NEDD4L and Ub-HA. Four hours before harvesting cells, MG132 (a proteasome inhibitor) or chloroquine (a lysosomal inhibitor) were added either separately or together to prevent degradation of the ubiquitinated substrates. Cells were lysed in denaturing conditions using 2% SDS in lysis buffer, followed by pull-down with anti-GFP antibody conjugated sepharose beads and analysed by immunoblotting. The result showed that both GSDMD and GSDME are targeted for proteasomal degradation following ubiquitination by NEDD4L (Fig. 4C). We also used THP-1 cells (WT and *NEDD4L* KO) to analyse ubiquitination of endogenous GSDMD and GSDME. Cells were lysed in denaturing condition and cell lysates used for immunoprecipitation with commercially available antibodies. We observed reduced GSDMD and GSDME polyubiquitination in the *NEDD4L* KO cells (Fig. 4D). Finally, to determine if this process involved direct ubiquitination of GSDMD and GSDME by NEDD4L we used purified recombinant NEDD4-2 (both WT and Cys942Ala mutant protein), E1 (UBA1), E2 (UbcH5b) and Ub to test if NEDD4-2 can directly ubiquitinate these proteins. GFP and GFP-tagged GSDMD and GSDME were obtained from transfected HEK293T cells as described in the Methods section. GFP protein was used as control. Importantly, NEDD4-2 (WT) but not the catalytically inactive enzyme (NEDD4-2 Cys942Ala mutant) ubiquitinated GSDMD and GSDME (Fig. 4E). Thus, GSDMD and GSDME are both NEDD4L /NEDD4-2 substrates.

### NEDD4L ubiquitinates lysine residues in GSDMD and GSDME

Next, we used constructs expressing full-length (FL), N- and C-terminal domains of GSDMD and GSDME proteins [12] to identify what regions are ubiquitinated by NEDD4L. As expected, the transfection of N-GSDMD caused significant cell death. All cells (floating and attached) were harvested and used for detection of ubiquitinated proteins. We observed that the N-terminal domain of GSDMD and both N- and C- termini of GSDME are ubiquitinated by NEDD4L (Supplementary Fig. S5A, B). We note that the N-GSDMD-GFP signal was very weak likely due to degradation when overexpressed with NEDD4L and ubiquitin.

**Fig. 5:**
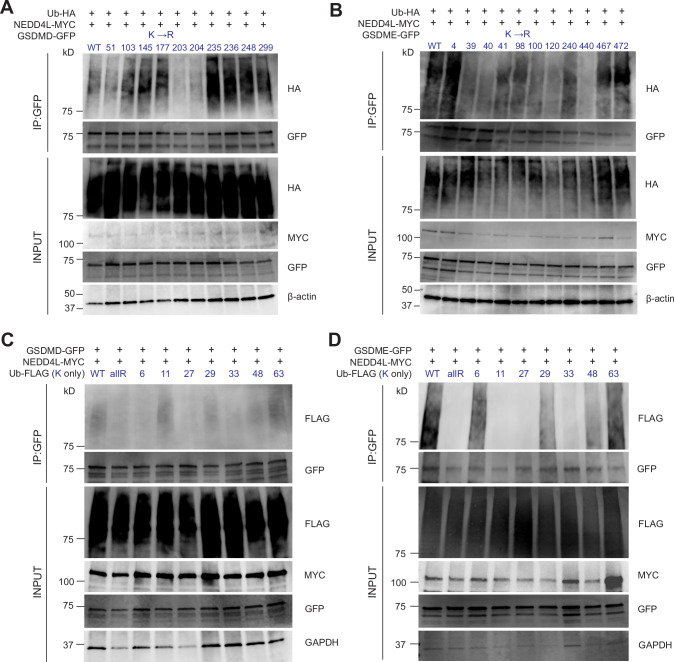
Identification of potential ubiquitination sites and specific Ub linkages in GSDMD and GSDME. **A**, **B** HEK293T cells were transfected with various K to R mutants expressing GFP-tagged GSDMD/E along with NEDD4L-MYC and Ub-HA. Samples were analysed after IP with anti-GFP antibody and blotting with the indicated antibodies (*n* = 3). **C**, **D** HEK293T cells were transfected with GFP-tagged GSDMD/E and NEDD4L-MYC along with Ub-FLAG plasmid that expresses Ub with only one type of Ub chain (K6; K11, K27; K29; K33; K48; K63) or WT Ub or Ub lacking all K (allR, all K mutated to R). Cells were harvested, lysed and IP reaction set up using anti-GFP antibody. Samples were washed and analysed by immunoblotting using the indicated antibodies showing the formation of unique branched ubiquitin chains in GSDMD and GSDME. Representative blots from three independent experiments are shown.

Lysine (K) residues are typical ubiquitination sites for most E3s. We used online prediction tools UBIPRED (https://flipper.diff.org/app/tools/info/2503) and BDM-PUB (http://bdmpub.biocuckoo.org/) to predict candidate K residues in GSDMD and GSDME that could be ubiquitinated (Supplementary Fig. S5C, D). Based on the scores in Supplementary Fig. S5C, D we selected 9 and 14 K residues in GSDMD and GSDME, respectively and mutated them to arginine (R). Both WT and mutant constructs were transfected in HEK293T cells, and ubiquitination assays were performed. We observed that NEDD4L ubiquitinates GSDMD at K51, K203 and K204 (all in N-terminal domain) whereas GSDME was mostly ubiquitinated at K39, K40, K120 in N-terminal domain and K440 in C-terminal domain (Fig. 5A, B). As the type of Ub chain linkage determines cellular fate of the substrate [25], we assessed the ubiquitin linkages for both these proteins. Ub has seven internal K residues (at positions 6, 11, 27, 29, 33, 48 and 63). Using a lysine-deficient Ub construct where all Lys residues were mutated to arginine residues (allR), individual arginine residues were mutated back to lysine residues to obtain K6, K11, K27, K29, K33, K48 and K63 Ub and one with all K (WT Ub). These Ub mutants were transfected along with MYC-NEDD4L and GFP-GSDMD/GSDME and ubiquitination was measured by immunoprecipitation and immunoblotting to identify the type of Ub chain linkages. GSDMD showed mixed chain formation with K11, K29 and K63 as major linkages involved. On the other hand, K6, K29, K48 and K63-linked Ub chains were observed for GSDME (Fig. 5C, D).

### NEDD4L mediated ubiquitination of GSDMD and GSDME prevents cell death

**Fig. 6:**
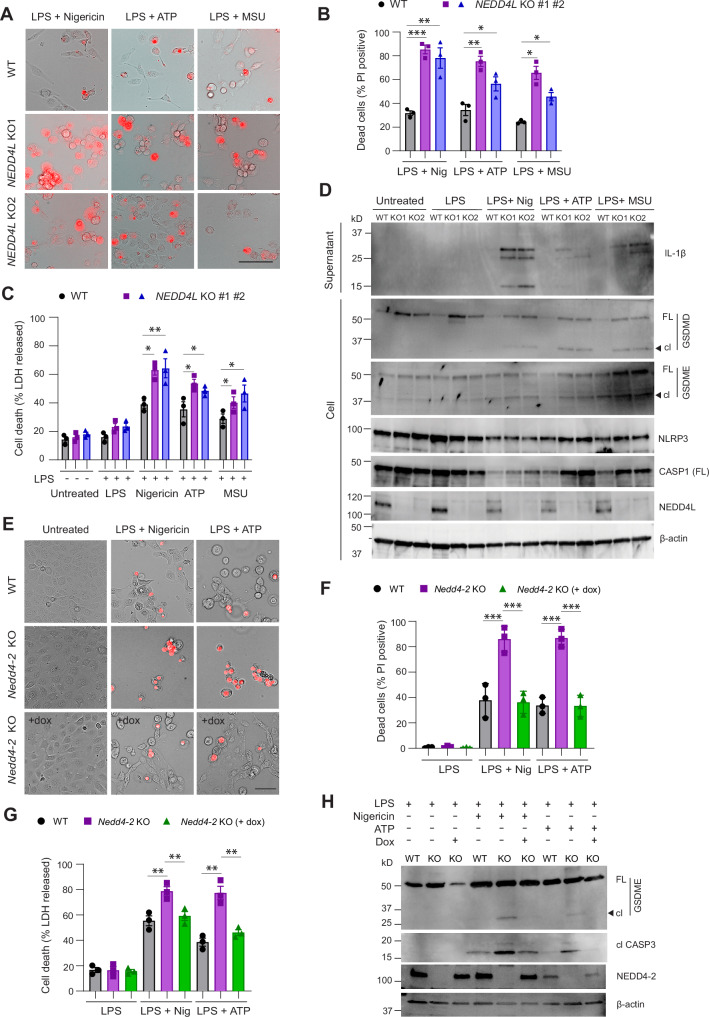
Loss of NEDD4L promotes GSDMD and GSDME activation and cell death. THP-1 cells were primed with LPS and treated with nigericin (5 μM, 1 h), ATP (2.5 mM, 1 h) or MSU (500 μg/ml, 5 h). **A** Representative images from propidium iodide (PI) stained cells and **B** their quantitation demonstrates a higher number of PI-positive cells and prominent cell swelling (pyroptotic morphology) in both *NEDD4L* KO clones, *NEDD4L* KO1 and *NEDD4L* KO2. Scale bar = 100 μm. **C** Histograms show LDH levels measured in culture supernatant after various treatments in WT and *NEDD4L* KO cells as above. **D** Immunoblots from the treated cells showing GSDMD activation and IL-1β release in the supernatant fraction of KO cells. **E** Representative images from propidium iodide (PI) stained CCD cells primed with LPS and treated with nigericin or ATP and **F** their quantitation is shown. **G** Histograms represent LDH levels measured in culture supernatant after various treatments. **H** Immunoblots from the treated cells showing GSDME cleavage in the *Nedd4-2* KO cells that was prevented after *Nedd4-2* re-expression. Data are Mean ± SEM (*n* = 3 independent experiments). Full-length (FL) and cleaved (cl) forms are shown. Statistical analysis was performed using the two-tailed unpaired Student’s *t*-test. *P < 0.05, **P < 0.01 and ***P < 0.001.

Gasdermins are essential effectors of pyroptosis, and GSDMD or GSDME cleavage is an established indicator of cells undergoing pyroptosis [1, 5]. To assess if NEDD4L mediated ubiquitination affects GSDM cleavage and cell death, we used mouse CCD cells and human myeloid THP-1 cells. *GSDMD* KO THP-1 cells [26] were used as a control. THP-1 (WT, *NEDD4L* KO and *GSDMD* KO) were primed with LPS (100 ng/ml, 2 h) and treated with various agents such as nigericin (5 μM, 1 h), ATP (2.5 mM, 1 h) or monosodium urate (MSU, 500 μg/ml, 5 h). All these agents are known to promote the formation of NLRP3 inflammasome, which results in the activation of caspase-1 that cleaves GSDMD and IL-1β to their mature forms. LPS increased NLRP3 expression in samples treated with NLRP3 agonists. However, IL-1β release and GSDMD activation was observed only in WT and *NEDD4L* KO cells but not in *GSDMD* KO THP-1 cells (Supplementary Fig. S6A). Importantly, *NEDD4L* KO cells showed increased release of mature IL-1β (cleaved 17 kD band) and GSDMD activation compared to WT. We also observed higher expression of cleaved GSDME in the treated *NEDD4L* KO cells (Supplementary Fig. S6A). We repeated the above treatments in two *NEDD4L* KO clones. Using propidium iodide (PI) staining, we observed increased cell death in the absence of *NEDD4L* (Fig. 6A, B). In the culture supernatants from *NEDD4L* KO cells, we observed significantly higher levels of LDH and presence of both full-length and mature IL-1β protein compared to the WT cells (Fig. 6C, D). Immunoblots from the cell lysates also indicated increased GSDMD and GSDME cleavage in *NEDD4L* KO cells, suggesting pyroptosis in these cells (Fig. 6D). Inflammasome activation can activate both apoptotic and pyroptotic pathways by inducing cleavage of initiator caspase (casp-8/casp-9) and executioner caspase (casp-3/casp-7) [27]. WT and *NEDD4L* KO cells were primed with LPS for 2 h and then treated with nigericin to monitor the rate of cell death with Incucyte using caspase-3/7 green dye to mark dead cells. We observed that *NEDD4L* KO cells undergo cell death significantly earlier than WT cells (Supplementary Fig. S6B, C showing images from 3 h).

**Fig. 7:**
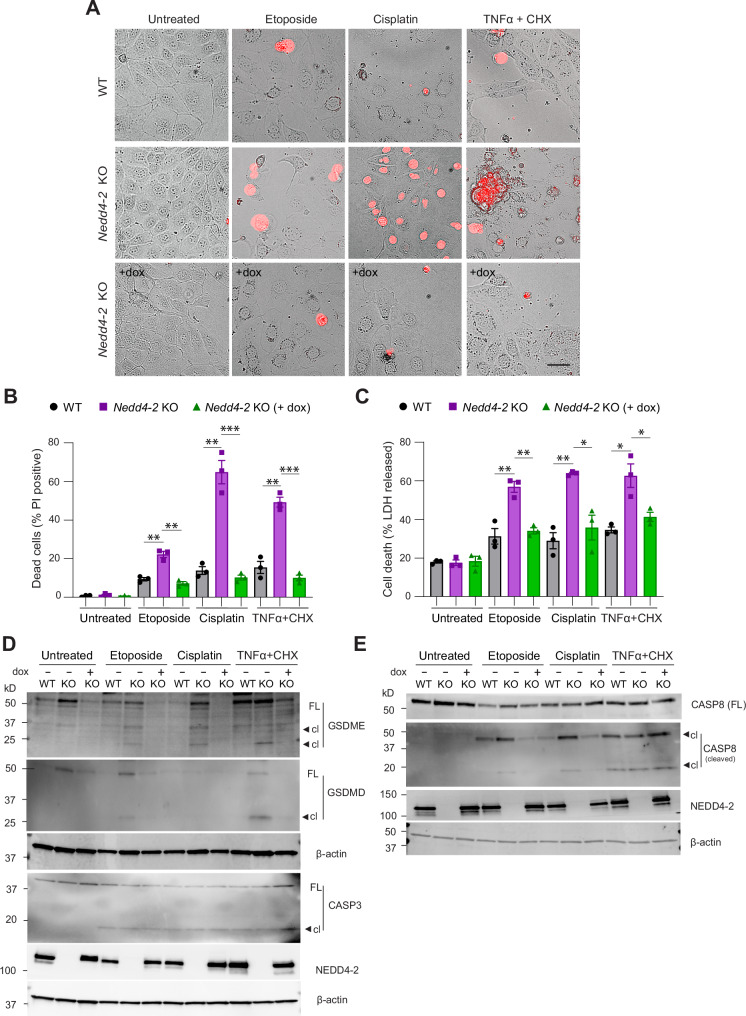
Loss of NEDD4-2 causes GSDMD and GSDME activation and cell death in CCD cells which is prevented when NEDD4-2 is re-expressed. CCD cells were treated with etoposide, cisplatin or TNFα/CHX for 24 h. **A** PI-stained images show increased pyroptotic cells in treated *Nedd4-2* KO cells that is prevented in KO cells re-expressing *Nedd4-2* after dox administration. Scale bar = 50 μm. **B** Histograms showing quantitation of PI-stained cells. **C** Histograms showing measurement of LDH in culture supernatant after treatment. **D**, **E** Immunoblots showing caspase-3, caspase-8 are cleaved after the various treatments in WT and *Nedd4-2* KO (+/− dox) cells. GSDMD and GSDME activation is observed in *Nedd4-2* KO cells that is abolished after *Nedd4-2* re-expression. Full-length (FL) and cleaved (cl) forms are shown. Data are Mean ± SEM (*n* = 3 independent experiments). Statistical analysis was performed using two-tailed unpaired Student’s *t* test. *P < 0.05, **P < 0.01 and ***P < 0.001.

To further assess if Gasdermin activation is dependent on NEDD4L we generated a doxycycline (dox) inducible CCD cell line that lacks *Nedd4-2* and restores *Nedd4-2* expression following treatment with dox (Supplementary Fig. S7A, B; [28]). CCD cells were treated with LPS for 24 h, thereafter media was replaced and cells exposed to nigericin or ATP. Nigericin and ATP-treated *Nedd4-2* KO cells showed cell swelling and there were significantly more PI-positive cells compared with WT or dox-treated *Nedd4-2* knockout cells (Fig. 6E, F). LDH levels were also elevated in the culture supernatants collected from nigericin and ATP-treated *Nedd4-2* KO cells and were restored after dox induction (Fig. 6G). Importantly, GSDME was activated in the *Nedd4-2* KO cells and this was prevented after *Nedd4-2* re-expression (Fig. 6H). These data clearly demonstrated that NEDD4-2 regulates GSDME activation. As chemotherapy drugs often induce pyroptosis through caspase-3 cleavage of GSDME [29], we treated CCD cells with 100 μM etoposide, 30 μM cisplatin or 30 ng/ml TNFα plus 100 μg/ml cycloheximide (CHX) for 24 h. CCD cells lacking *Nedd4-2* showed a higher number of PI-positive cells and significantly increased LDH levels in the supernatant (Fig. 7A–C). Immunoblots showed caspase-3 cleavage after 24 h of drug treatment and increased GSDME activation in the *Nedd4-2* KO cells (Fig. 7D). Both etoposide and cisplatin treatment also caused increased caspase-8 activation in the *Nedd4-2* KO cells. TNFα/CHX resulted in caspase-8 activation that was similar in the three different cell types (Fig. 7E). Caspase-8 can directly cleave and activate GSDMD [30, 31]. While all the drug-treated *Nedd4-2* KO cells displayed a reduction in full-length GSDMD, cleaved GSDMD was not observed in the cisplatin-treated cells. Importantly, when *Nedd4-2* was re-expressed in these cells (by dox addition), cell death was prevented and was associated with reduced LDH levels (Fig. 7A–C). Immunoblots further showed GSDMD and GSDME were activated and cleaved in *Nedd4-2* KO cells but dox-induced *Nedd4-2* re-expression prevented this (Fig. 7D). These results clearly establish that NEDD4-2 regulates GSDMD and GSDME activation and pyroptosis.

### NEDD4L-deficiency exacerbates infection-induced cell death

**Fig. 8:**
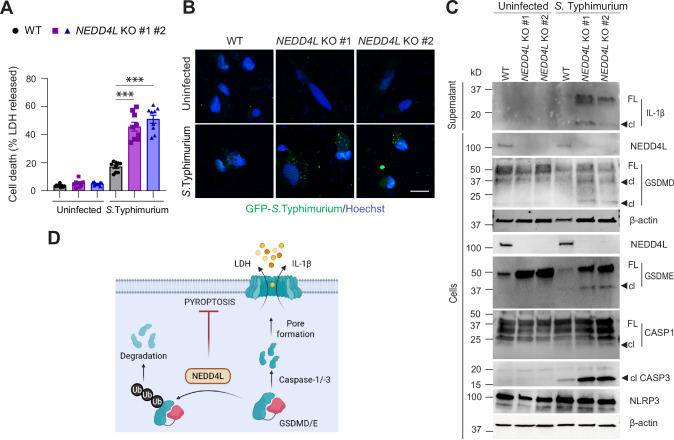
*NEDD4L* deficiency promotes cell death induced by *Salmonella* Typhimurium. **A** Histograms showing increased LDH levels in culture supernatant from infected *NEDD4L* KO THP-1 cells. **B** Phagocytosis of GFP labelled *S*. Typhimurium in macrophage derived THP-1 cells. Cells were counterstained with Hoechst nuclear stain (blue). Scale bar = 50 μm. **C** Immunoblots show bacterial infection results in NLRP3 activation, caspase-1, caspase-3 cleavage with increased GSDMD, GSDME activation and IL-1β release in *NEDD4L* KO THP-1 cells. Full-length (FL) and cleaved (cl) forms are shown. Data are Mean ± SEM. Statistical analysis was performed using the two-tailed unpaired Student’s *t*-test. ***P < 0.001. Representative data from three independent experiments are shown. **D** A model showing that NEDD4L polyubiquitinates GSDMD and GSDME. This regulates their intracellular levels and prevents excessive cell death.

We then tested if NEDD4L-mediated ubiquitination attenuates bacterial infection-induced cell death. THP-1 cells (WT and *NEDD4L* KO) were differentiated into macrophages and then infected with *S*. Typhimurium. Samples were collected after 24 h when a marked increase in the number of floating dead cells was observed in *NEDD4L* KO wells. This correlated with significantly elevated LDH levels in the *NEDD4L* KO clones compared to the WT (Fig. 8A). Confocal imaging indicated the presence of THP-1 cells containing engulfed GFP-positive *S*. Typhimurium with increased bacterial load in the *NEDD4L* KO cells compared to the WT cells (Fig. 8B). Immunoblots showed increased IL-1β release in the culture supernatant and cell lysates also revealed GSDMD and GSDME activation along with increased levels of cleaved caspase-3 in the infected *NEDD4L* KO cells (Fig. 8C). While NLRP3 expression was higher and caspase-1 activation occurred in the infected cells, there was no difference between the WT and *NEDD4L* KO cells. These data indicate that NEDD4L prevents cell death induced by *S*. Typhimurium infection via both apoptotic and GSDMD/E mediated pyroptosis.

## Discussion

Gasdermin D and E are two members of the family of membrane pore-forming proteins that have an essential role in mediating pyroptosis [1, 2]. This study identifies for the first time that the HECT E3 ligase, NEDD4L (mouse NEDD4-2) plays a crucial role in regulating intracellular GSDMD and GSDME levels. NEDD4L polyubiquitinates GSDMD and GSDME with the formation of several typical and atypical Ub chains. Importantly, using *Nedd4-2* knockout mice, we found increased membrane localisation of GSDMD and GSDME in the lungs and kidneys that likely contributes to the respiratory distress and kidney damage observed in *Nedd4-2* deficient mice. We found that cell death occurs rapidly in *NEDD4L* KO cells with GSDMD/GSDME activation, suggesting that NEDD4L-mediated ubiquitination plays an important role in preventing cell death, acting as a brake by keeping intracellular GSDMD and GSDME protein levels low (Fig. 8D).

Gasdermins have been proposed to contribute to other types of cell death such as necroptosis or apoptosis, depending on the cell type and the stimuli [12, 32]. Activated GSDMD localises to mitochondria and lysosomes prior to the plasma membrane during pyroptosis [33]. N-GSDME forms pores in the mitochondrial membrane resulting in the loss of membrane potential and apoptosis [34]. In macrophages, mitochondrial GSDMD pores can also initiate necroptosis by switching on the RIPK1/RIPK3/MLKL-pathway [32] while N-GSDMD was shown to predominantly localise to the lysosomal membranes in neutrophils [35]. Other studies suggest non-cell death roles for GSDMs such as membrane repair [36, 37]. Understanding how GSDM pore formation can be controlled has therefore received much attention. While proteolytic cleavage at the linker region is necessary for the release of NTD and GSDM activation, recent studies have identified unique pathways that can result in the formation of pores by full length GSDMD and GSDME [8, 9]. Post translational regulatory mechanisms are emerging as important regulators of GSDM activity [11, 12, 38].

NEDD4L is expressed in most tissues [18, 19] and plays important roles in several signaling pathways such as inhibition of TGF-β signaling, autophagy, antiviral immunity and allergic inflammation [39–42]. It is a key regulator of several ion channels and transporters such as ENaC that has a critical function in tissues such as the kidney and lung [19]. In mice, a complete loss of *Nedd4-2* results in perinatal lethality that has been attributed to the upregulation of ENaC critical for fetal lung fluid clearance and proper airway surface hydration [20]. The data presented in this study clearly demonstrate a role for GSDMD and GSDME in lung and kidney injury in these mice. Typically, we find increased expression of apically localised GSDMD protein (and not GSDME) in the epithelial cells of *Nedd4-2* KO lungs. In contrast in the kidney, increased expression of apically localised GSDME protein (and not GSDMD) is apparent in the tubular epithelial cells of *Nedd4-2* KO mice, suggesting tissue-specific regulation. We then used a high Na^+^ diet as an inducer of kidney injury in two different mouse lines - mice heterozygous for *Nedd4-2* (*Nedd4-2*^*+/−*^) or with kidney tubule specific loss of *Nedd4-2* (*Nedd4-2*^*Ksp1.3*^ mice). In the heterozygous mice, high Na^+^ intake for a longer time of 3 months did not result in loss of kidney function as the GFR remained unchanged. However, it caused kidney damage, proteinuria, polyuria, polydipsia and elevated GSDMD and GSDME expression in *Nedd4-2*^*+/−*^ mice. BMDMs obtained from these mice also showed that high Na^+^ caused GSDMD and GSDME activation suggesting their role in mediating cell death in cells/tissues with reduced NEDD4-2. Kidney specific knockout, *Nedd4-2*^*Ksp1.3*^ mice do not tolerate high salt diet and develop end stage renal disease within 3 weeks of this diet [22]. Our data show that NEDD4-2 expression declines when mice were fed a high Na^+^ diet. Dietary Na^+^ intake impacts *Nedd4-2* abundance [43, 44]. For example, *Nedd4l* transcript levels are lower in Dahl salt-sensitive (DS) rats compared to their normotensive littermate, the Dahl salt-resistant (DR) rats under either low or high Na^+^ intake [43]. Reduced expression of NEDD4L is also correlated with disease progression in several conditions such as diabetic kidney disease [45, 46]; clear cell renal cell carcinoma [47] and is suggestive of increased kidney injury in this group. Furthermore, we find that high Na^+^ intake in *Nedd4-2*^*Ksp1.3*^ mice results in GSDMD and GSDME activation, elevated IL-1β levels, cell death and increased recruitment of macrophage and neutrophils to the damaged tubules exacerbating kidney injury. Increased cleavage of both GSDMD and GSDME was observed in the high Na^+^ diet fed *Nedd4-2*^*Ksp1.3*^ mice. The large reduction in full length GSDME in high salt fed KO kidney samples is likely due to increased activation and cleavage of GSDME without any transcriptional upregulation. High salt intake has been shown to downregulate the transcription factor Nrf2 in a sodium dependent manner in the mouse kidney collecting duct cells [48]. Further, binding of Nrf2 to the *GsdmD* promoter inhibits its expression [49]. It is plausible that high Na^+^ intake reduces Nrf2 expression and activity which in turn promotes *GsdmD* expression in the *Nedd4-2*^*Ksp1.3*^ mice. Upregulation of *GsdmD* likely results in increased full length GSDMD protein despite increased cleavage observed in this group. Based on our findings here we propose that (i) NEDD4-2 regulates GSDMD and GSDME proteins in a tissue specific manner and (ii) increased protein levels of active GSDMD and GSDME result in cell death that is responsible for epithelial cell loss in *Nedd4-2* deficient mice.

Reduced NEDD4L levels and *NEDD4L* variants have been implicated in several human pathologies including IPF (idiopathic pulmonary fibrosis) [50], cystic fibrosis [51]; diabetic nephropathy [45], and hypertension [52]. It is possible that some of these pathologies are associated with dysregulation of GSDMD and GSDME and inflammatory cell death. High expression of GSDMD is associated with many pulmonary conditions such as asthma, COPD and acute lung injury [53–55]. GSDMD was found to cause silica induced pulmonary inflammation and fibrosis [54] with reduction in GSDMD expression preventing inflammation and damage induced by acute lung injury [55]. In diabetic nephropathy, GSDMD mediates pyroptosis in both podocytes [56] as well as renal tubular cells [57]. On the other hand, chemotherapy drug induced nephrotoxicity involves renal tubular epithelial cell pyroptosis mediated by ROS-JNK-caspase 3-GSDME signaling pathway [58]. It has been reported that loss of GSDMD prevents kidney injury and fibrosis [59, 60]. A role for GSDMD in hyperuricemia nephropathy was also observed that resulted in loss of renal function, fibrosis and reduced body weight. Notably, these changes were not observed in GSDMD knockout mice [61]. GSDMD expression was reported to be higher in renal tubules of biopsies obtained from T2 diabetic patients with kidney disease and reduced GSDMD levels correlated with reduced fibrosis and pyroptosis in high glucose treated HK2 cells [62]. Similarly, GSDME expression was found to be elevated in obstructive kidney disease resulting in renal tubule damage, cell loss, fibrosis and inflammation that was ameliorated in *GsdmE* knockout mice kidneys [63]. Importantly, loss of GSDME in mice attenuated inflammation and acute kidney injury induced by cisplatin or renal ischemia reperfusion injury (IRI) [64]. Further, GSDME is upregulated during renal IRI and exacerbates renal injury by causing pyroptosis, inflammation and mitochondrial damage while knockout of *GsdmE* prevents this [65]. NEDD4L is also involved in other inflammatory diseases including atherosclerosis [66], cardiovascular disease [67] and has been identified as a key target gene involved in the occurrence, progression and treatment of atherosclerosis [68]. Both GSDME [69] and GSDMD [70] mediated pyroptosis play a role in the progression of atherosclerosis. Macrophage GSDMD promotes foam cell formation in an IL-1β dependent manner, inhibits reverse cholesterol transport and leads to atherosclerosis in hyperlipidemic mice [70]. Single-cell RNA sequencing on human carotid atherosclerotic plaques revealed that *GSDME* is mainly expressed in macrophages, which contribute to disease progression by inducing inflammation and pyroptosis [69]. These previous findings suggest that the higher expression of GSDMD and GSDME in *Nedd4-2* KO mice lungs and kidney causes tissue injury due to increased inflammation and cell death.

NEDD4L controls GSDMD and GSDME stability which is reflected by the longer half-life of both proteins in knockout cells and higher expression of these proteins in *NEDD4L* knockout cells and tissues (mouse epithelial and human myeloid cells). While most known substrates of NEDD4L/NEDD4-2 are membrane proteins, it also targets cytoplasmic proteins such as tyrosine kinase Syk, TRAF-3 [41, 42]. Both GSDMD and GSDME are lysine rich proteins and are predicted to have multiple sites of ubiquitination. NEDD4L interacts with both GSDMD and GSDME, and ubiquitinates these proteins. We observed that the NTD pore forming domain of GSDMD was predominantly ubiquitinated by NEDD4L with K51, K203, K204 being identified as the major sites that are modified. Contrary to this, both NTD and CTD of GSDME were modified by NEDD4L. A previous study [17] showed ubiquitination of GSDMD by SYVN1 at K203 and K204 that involves K27 polyubiquitin chain formation and results in enhanced pyroptosis. However, we found K11, K63 and K29 as the predominant Ub linkages mediated by NEDD4L that led to reduced pyroptosis. Further, we observed NEDD4L ubiquitinates K39, K40, K120 and K440 in GSDME and this involves K6, K29, K48 and K63 linked polyubiquitin chain formation. The various type of Ub linkages that polyubiquitin chains adopt give rise to distinct conformations that cause different cellular outcomes [25, 71] and may contribute to the increased cell death in *NEDD4L* KO cells.

Various NLRP3 inflammasome activators like nigericin, ATP and MSU cause increased cell death in THP-1 cells lacking *NEDD4L*. We found that *NEDD4L* KO cells show GSDMD and GSDME activation, higher LDH release and dying cells appear swollen resembling the characteristic pyroptotic cells. *NEDD4L* KO cells also undergo cell death faster than the WT cells. Similar results were also seen in *Nedd4-2* KO kidney cortical collecting duct cells (CCD) when treated with these agents. This was rescued after *Nedd4-2* was re-expressed in the knockout cells suggesting NEDD4-2 regulates GSDME. Additionally, we tested other cell death inducers in the CCD cells. Apoptosis was induced using chemotherapy drugs such as etoposide, cisplatin or TNFα and cycloheximide treatment. These drugs activate caspase-3 that can cause GSDME cleavage resulting in pyroptosis [29]. In the kidney, etoposide and cisplatin also activate caspase-8 [72, 73] while TNFα/CHX is an established activator of caspase-8 [74]. It is known that caspase-8 activates GSDMD [30, 31]. We found that caspase-3 and caspase-8 was induced similarly in all cells by these drugs implying cell death by apoptosis is identical. Remarkably, increased GSDMD/GSDME activation and LDH release was observed in *Nedd4-2* KO cells suggesting increased pyroptotic cell death. Further, when *Nedd4-2* was re-expressed in the knockout cells by addition of dox, GSDMD/GSDME activation and LDH levels were reduced. It has been shown that in cells with high GSDME expression, active caspase-3 causes GSDME activation and pore formation, resulting in osmotic swelling and pyroptosis while low GSDME expressing cells die by apoptosis [75]. These observations point to a prosurvival function for NEDD4L in preventing GSDMD and GSDME mediated cell death in both myeloid and epithelial cells.

We also observed that *S*. Typhimurium induced significantly more cell death in *NEDD4L* deficient THP-1 derived macrophages compared to the control cells, and this was associated with increased GSDMD and GSDME cleavage and IL-1β release. *S*. Typhimurium infection is known to induce apoptosis in infected macrophages [76] and can also initiate pyroptosis by activation of the NLRC4 and NLRP3 inflammasome, resulting in caspase-1 cleavage, GSDMD activation and IL-1β release [77–79]. Such bacterial infections induced pyroptosis in host cells often acts as a mechanism to prevent bacterial replication [80]. *S*. Typhimurium infection activates both GSDMD and GSDME [81]. It is likely that increased GSDMD and GSDME in *NEDD4L* deficient cells augments death of infected cells by both pyroptosis and apoptosis.

In conclusion, our work demonstrates that NEDD4L is a critical regulator of GSDMD and GSDME stability and intracellular concentrations, thereby inhibiting their ability to form membrane pores (pore density) to prevent cell death.

## Materials and methods

### Antibodies

The following antibodies were used: anti-NEDD4-2 (produced in-house [82]); anti-GFP (600-101-215, Rockland, Limerick, PA, USA); anti-FLAG (M2, F3165, clone M2, Sigma-Aldrich); anti-HA (3F10, Roche); anti-MYC tag (9B11, 2276, Cell Signaling Technology, Beverly, MA, USA); anti-Ub HRP (P4D1, sc-8017, Santa Cruz Biotechnology); anti-GSDMD (EPR19828, ab209845, Abcam); anti-DFNA5/GSDME (EPR19859, ab215191); anti-cleaved GSDMD (E7H9G, 36425, Cell Signaling Technology); anti-caspase-1 (Casper-1, AG-20B-0042, AdipoGen Life Sciences); anti-cleaved caspase-8 (8592, Cell Signaling Technology, Beverly, MA, USA) and anti-NLRP3 (D4D8T, Cell Signaling Technology); anti IL-1β (AF-401-NA, R and D Systems); anti-caspase-3 (9662, Cell Signaling Technology, Beverly, MA, USA); anti-cleaved caspase-3 (9664, Cell Signaling Technology); anti-GST (G7781, Sigma-Aldrich); anti-F4/80 (MF48000, Invitrogen, Waltham, MA, USA); anti-Ly6G (551459, BD Biosciences, Franklin Lakes, NJ, USA); anti-β-actin (A1978, Sigma-Aldrich) and anti-GAPDH (Cell Signaling Technology, 14C10). The following secondary antibodies were used: donkey anti-rabbit horseradish peroxidase, anti-mouse horseradish peroxidase, ECL plex goat anti-mouse and anti-rabbit Cy5 from GE Healthcare (Buckinghamshire, UK); peroxidase conjugate IgG fraction anti rabbit IgG, light chain specific (5A6-1D10) was obtained from Jackson Laboratories; anti-goat Alkaline Phosphatase, anti-mouse Alkaline Phosphatase, anti-rabbit Alkaline Phosphatase and anti-rat Alkaline Phosphatase from Merck Millipore (Billerica, MA, USA). Alexa-Fluor donkey anti-rabbit 488 was purchased from Thermo Fisher Scientific (Waltham, MA, USA). Immunoblots were developed using ECL prime (GE Healthcare) and West Femto (Thermo-Fisher Scientific) or ECF (GE Healthcare).

### Expression vectors

Plasmids expressing human GSDMD-GFP, GSDME-GFP, GSDMD-N-GFP and GSDME-N-GFP were from the Alnemri laboratory (Thomas Jefferson University, Philadelphia) [12], human GSDMD-C-GFP, GSDME-C-GFP were generated using appropriate primers (Supplementary Table 1). Plasmids coding K-R mutants for GSDMD-GFP and GSDMD-GFP were generated using site-directed mutagenesis. NEDD4-2, NEDD4-2 cysteine mutant constructs have been described previously [83]. To obtain MYC-tagged NEDD4L-WT, NEDD4L ORF of the corresponding fragments (aa 363–423) were amplified from the pBS(+)-NEDD4L using the following PCR primers listed in Supplementary Table 1 and subcloned into pCMV-Myc vector GEX-4T-3 using BglII and KpnI sites. A catalytically inactive NEDD4L (Cys942Ala, mut) was generated by PCR mutagenesis. The HA-ubiquitin expression plasmid was kindly provided by Dirk Bohmann (University of Rochester, Rochester, NY). FLAG-tagged Wild-type (WT) ubiquitin and KallR mutant (ubiquitin in which all Lysine (K) residues are mutated to Arginine (R) constructs were purchased from the MRC Protein Phosphorylation and Ubiquitylation Unit, University of Dundee. As previously described [84], plasmids coding for only one specific polyubiquitin chain mutant was generated in our laboratory by reverting specific Arginine, R residues back to Lysine, K in the KallR construct (R6K, R11K, R27K, R29K, R33K, R48K, R63K).

### Cell culture and transfection

U2OS, HEK293T and A549 cell lines were maintained in Dulbecco’s Modified Eagles Medium (DMEM, Sigma-Aldrich) supplemented with 10% foetal bovine serum (JRH Biosciences, Lenexa, KS, USA), 0.2 mM L-glutamine (Sigma-Aldrich), 15 mM HEPES (Sigma-Aldrich) and 100 µM penicillin/streptomycin (Sigma-Aldrich) in a humidified incubator at 37 °C with 10% CO_2_. THP-1 cells (Wild-type, WT; *NEDD4L* KO and *GSDMD* KO) were maintained in RPMI 1640 (Sigma-Aldrich) containing 10% foetal bovine serum (JRH Biosciences, Lenexa, KS, USA), 0.2 mM L-glutamine (Sigma-Aldrich), 15 mM HEPES (Sigma-Aldrich) and 100 µM penicillin/streptomycin (Sigma-Aldrich) in a humidified incubator at 37 °C with 10% CO_2_. The *GSDMD* KO THP-1 cells were treated with 1 μg/ml dox for 72 h, as expression of the sgRNA (GFP) is doxycycline inducible. Kidney cortical collecting duct (CCD) cell line CCD-N21 were grown in DMEM/F12 media (Gibco) supplemented with 2% fetal calf serum, 1% ITS, 1 nM 3,3′,5-triiodo-L-thyronin, 10 ng/mL EGF and 50 nM dexamethasone (all from Sigma-Aldrich). Where indicated, THP-1 cells were treated with 100 nM PMA (Sigma-Aldrich) for 48 h to differentiate into macrophages and treated with 5 µM nigericin, 2.5 mM ATP, 500 μg/ml MSU (Sigma-Aldrich), for inducing cell death. CCD cells were treated with 100 μM etoposide (Merck, E1383), 30 μM cisplatin (Calbiochem, 232120) or 30 ng/ml TNFα (Merck, T7539) along with 100 µg/ml cycloheximide (Selleck Chemicals, Houston, TX, USA) for 24 h. Transfection of plasmid DNA was performed using Lipofectamine 3000 (Invitrogen) and siRNA mediated *NEDD4L* knockdown was performed using Lipofectamine RNAimax (Invitrogen) according to the manufacturer’s instructions. All cell lines were tested for mycoplasma contamination.

### Bimolecular Fluorescence Complementation (BiFC) analysis

BiFC analysis for identifying if NEDD4L interacts with GSDMD and/or GSDME was performed as described previously with minor modifications [85]. To generate BiFC constructs for GSDMD, GSDME and NEDD4L, these molecules were amplified from GSDMD-GFP, GSDME-GFP and NEDD4L-MYC constructs using the PCR primers with added BglII and KpnI sites listed in Supplementary Table 1. The amplified fragments were cut with BglII/KpnI and subsequently subcloned in-frame into pBiFC-VN173 and pBiFC-VC155, respectively. All final constructs were confirmed by DNA restriction enzyme digestion and DNA sequencing. All DNA restriction enzymes used in this work were purchased from NEB (Ipswich, MA, USA). For detection by immunoprecipitation, HEK293T cells were co-transfected with pBiFC-GSDMD-VC155 or pBiFC-GSDME-VC155 and pBiFC-NEDD4L-VN173 (150 ng each) using Lipofectamine 3000 reagent (Invitrogen). After 24 h, cells were harvested, washed in PBS and lysed in NP40 buffer (150 mM NaCl, 1% NP-40, 50 mM Tris-HCl, pH 8.0) containing 1X Halt protease and phosphatase Inhibitor cocktail, EDTA (Thermo Fisher Scientific). The cell lysates were then incubated with GFP-Trap beads for 2 h at 4 °C with gentle rocking. Beads were washed (four washes in lysis buffer) and proteins eluted by boiling in 2x Laemmli buffer for 5 min at 95 °C and subjected to immunoblotting. 20 μg samples were loaded as input. For cell imaging, U2OS were seeded onto 22 mm glass coverslips (Thermo Fisher Scientific) and incubated overnight at 37 °C in a 10% CO_2_ incubator. The next day, the cells were co-transfected with 150 ng of the pBiFC-GSDMD/GSDME-VC155 and pBiFC-NEDD4L-VN173 (human) along with 100 ng of mCherry-tubulin as a transfection reporter plasmid, using Lipofectamine 3000 reagent (Invitrogen). pBiFC-GSDMD-VC155, pBiFC-GSDME-VC155 and pBiFC-NEDD4L-VN173 (human) were transfected separately as controls. 24 h after transfection, cells were fixed with 4% paraformaldehyde (Sigma-Aldrich) in PBS and BiFC imaged by confocal microscopy using a LSM 800 confocal microscope (Zen 2011 Black Edition, Carl Zeiss Microscopy, Jena, Germany). At least 100 cells were counted in five different areas in two independent experiments to quantify BiFC-positive cells.

### Generation of *NEDD4L* KO THP-1 cells and dox inducible *Nedd4-2* CCD cells

*Nedd4-2* deficient CCD cells were generated as described [22]. For generating *NEDD4L* knockout (KO) THP-1 cells, two sgRNAs targeting exons 7 and 10 of the *NEDD4L* gene were designed using the ChopChop algorithm (https://chopchop.cbu.uib.no/). The following sgRNA sequences were selected: *NEDD4L*-sg1: 5′-GGAGCGACCCTATACATTTA-3′ (PAM: AGG) targeting exon 7 and *NEDD4L*-sg2: 5′-GATGTTATTGTCCGACTCCG-3′ (PAM: AGG) targeting exon 10 and cloned into a lentiviral CRISPR/Cas9 expression vector (pLentiCRISPRv2, Addgene). Lentivirus production was performed by transfecting HEK293T cells and supernatant containing viral particles collected after 48 h. THP-1 cells (5 × 10⁵) were resuspended in viral supernatant supplemented with polybrene (0.4 µg/mL; Sigma-Aldrich), seeded in 6-well plates and incubated at 37 °C for 48 h. Thereafter, the media were replaced and supplemented with 1 μg/mL puromycin (Sigma-Aldrich) for a further 2–3 days to select for transfected cells. Surviving cells were seeded in a few 96-well plates by limiting dilution to isolate single colonies. *NEDD4L* loss was confirmed by immunoblotting and genomic analysis. For the genomic validation, genomic DNA was extracted from the selected clones, and PCR was performed to amplify the sgRNA target regions in exons 7 and 10. Further gene editing and indel formation were obtained by performing Sanger sequencing on PCR products, followed by TIDE (Tracking of Indels by Decomposition) analysis. Colonies with confirmed *NEDD4L* deletion were expanded for further characterisation and functional studies.

For the generation of inducible *Nedd4-2* CCD cells, the full-length mouse *Nedd4-2* cDNA (NCBI RefSeq: NM_001114386.1) was cloned into the pENTR™/D-TOPO™ vector (Invitrogen) and recombined into the pInducer20 destination vector using Gateway® LR Clonase™ II (Invitrogen). The recombination products were transformed into Stbl3™ E. coli. Lentivirus production was carried out by co-transfecting HEK293T cells with pInducer20-mNEDD4-2 (2.1 µg), Gag-Pol (1.25 µg), Rev (1 µg), and VSV-G (0.625 µg) using Lipofectamine 3000 (Invitrogen). Viral supernatant was collected after 48 h, filtered, and used to transduce *Nedd4-2* KO CCD cells in the presence of 4 µg/mL polybrene. After 48 h, cells were selected with G418 (444 µg/mL; 24 h). Single-cell clones were isolated by limiting dilution in 96-well plates, expanded, and induced with 2 µg/mL doxycycline (dox) for 24 h to confirm NEDD4-2 expression by immunoblotting. Clones with robust inducible expression were expanded and used for further analyses.

### Protein stability assays

THP-1 and CCD (WT and *NEDD4L* KO) were seeded in 60 cm dish (1 × 10^6^ cells) and allowed to grow (24 h, 37 °C). Next day, cells were treated with 100 μg/ml cycloheximide for the desired time intervals. Cells were collected and lysed in NP40 lysis buffer containing HALT protease and phosphatase inhibitor cocktail. Samples were lysed by two freeze-thaw cycles followed by incubation on ice for 30 min. Lysates were cleared by centrifugation (13,000 rpm, 10 min, 4 °C) and then 30 μg protein used for immunoblotting as described below.

### Preparation of recombinant NEDD4-2

The plasmids expressing NEDD4-2 WT (ΔC2,WW1,WW2) or NEDD4-2 mut (ΔC2,WW1,WW2) Cys mutant GST expression plasmids were generated previously in our laboratory [86]. BL21 star (*E coli*; DE3 pLysS; Invitrogen) cells containing these plasmids were diluted 1:25, grown overnight to log phase at room temperature, and then induced with 1 mm isopropyl β-d-thiogalactoside at room temperature for 5–6 h. GST only fusion protein was prepared similarly but grown at 37 °C. Following induction, GST fusion proteins were purified. Briefly, bacterial cell pellets were resuspended in phosphate-buffered saline (PBS), lysed by sonication, and clarified by centrifugation at 10,000 rpm for 10 min. Glutathione-Sepharose (Amersham Pharmacia Biotech) was incubated with the cleared lysate for 2 h at room temperature, and then the beads were washed three times with PBS. GST fusion proteins were eluted using glutathione buffer (50 mM Tris, 10 mM reduced glutathione; pH 8.0), run on SDS-polyacrylamide gels and Coomassie stained. Lanes expressing the recombinant protein were pooled and protein concentration measured using a BCA kit (Pierce).

### In vitro ubiquitination assays

This was done according to previously published method with minor modifications [87]. HEK293T cells were transfected with 5 µg of GFP-N1 or GFP-GSDMD/E using Lipofectamine 3000 (Invitrogen). After 24 h cells were washed with PBS and lysed in 800 µl Verhagen lysis buffer (150 mM NaCl, 20 mM Tris-HCl pH 7.5, 2 mM EDTA, 10% glycerol, and 1% Triton-X) for 30 min. Cell lysate was clarified (13,000 rpm, 10 min) and 50 µL of lysate was reserved as the input and the remaining lysates were then added to 20 µl of magnetic protein G-Sepharose (Amersham Biosciences) and 1 µL of rabbit anti-GFP (equilibrated in 1 ml of Verhagen’s lysis buffer) and incubated at 4 °C overnight while rotating. The protein-bound beads were then washed twice in lysis buffer then once with ubiquitin assay buffer (40 mM Tris-HCl pH 7.5, 10 mM MgCl_2_, 0.6 mM DTT) and eluted with 40 μl glycine (200 mM, pH 2.5) and immediately neutralised with 10 μl 1 M Tris (pH 10.4). This was repeated three times and the pooled eluates were diluted in ubiquitin assay buffer (200 µg/ml diluted) for 30 min while mixing at room temperature. For ubiquitination assay E1 (~80 ng, Boston Biochem kit, K-995), E2 (1 µM, UbcH5b, Boston Biochem, E2-622), E3 (0.5 µM, GST-NEDD4-2 WT or mutant, purified above) and 15 µl of purified GFP-GSDMD/GSDME in ubiquitin assay buffer containing 2 mM ATP (total volume of 30 µl) were added and reactions performed at 37 °C for 90 min. Controls included lanes without E3 or catalytically inactive mutant protein and ubiquitination of GFP protein. The reactions were stopped by the addition of 2× Laemmli sample buffer and boiling for 5 min prior to immunoblot analysis.

### Cell death assays and Propidium Iodide staining

After treatment with the various drugs, cells were cultured for 15 min with 1.0 μg/ml propidium iodide (P4864, Sigma-Aldrich) and observed and imaged using ZOE cell imager (Bio-Rad). The dead/floating cells (THP-1 and CCD) were collected by spinning the supernatant at 13,000 rpm, 10 min, 4 °C. The cleared supernatant was transferred to a fresh tube. The remaining cells were harvested using trypsin and cell scraping, washed once with PBS and lysed in NP40 lysis buffer containing Halt protease inhibitor. For analysing IL-1β in culture supernatant, proteins were precipitated using the chloroform-methanol protocol as described previously [88]. Briefly, after the desired treatment, the culture media were spun at 2000 rpm for 5 min to pellet cell debris. Supernatants were transferred to a fresh tube and an equal volume of methanol, and 0.25 volumes of chloroform was added to each tube. Samples were thoroughly mixed by inverting vigorously and centrifuged at 13,000 rpm for 10 min. The upper phase was discarded, and the same volume of methanol added to the interphase, mixed by inverting and the samples were spun again at 13,000 rpm for 5 min. The supernatants were discarded, and protein pellets were dried at room temperature, resuspended in 2× Laemmli buffer, and boiled for 10 min at 98 °C until dissolved. The resuspended proteins were fractionated on 4–20% SDS-PAGE gels (Bio-Rad) followed by electroblotting onto PVDF membranes. Blots were probed with appropriate antibodies.

### LDH release assay

Pyroptotic cell death was determined by quantitating the amount of LDH released into cell culture supernatants using the CytoTox96 LDH release kit (Promega). Cell culture supernatant was collected after various treatments and LDH levels measured according to the manufacturer’s protocol.

### Real time imaging

For real-time imaging, THP-1 cells were seeded in 24-well plates (0.5 × 10^5^/well), PMA was added, and cells allowed to attach and differentiate (48 h). Cells were pre-treated with Nuclight rapid red (#4717) and caspase-3/7 green reagent (#4440, both from Sartorius and used at 1:2000) for 4 h, and LPS was added (100 nM; 2 h). Thereafter cells were treated with nigericin (Sigma-Aldrich) and then imaged at 30 min intervals for 2–4 h on the IncuCyte® S3 Live Cell Analysis System at 20× magnification. Data was analysed using IncuCyte S3 software.

### Immunoprecipitation and immunoblotting

For co-immunoprecipitation assays to detect protein interaction between endogenous proteins, WT THP-1 and CCD cells were used with *NEDD4L* KO and *GSDMD* KO cells as controls. Cells were harvested and lysed in Verhagen lysis buffer (150 mM NaCl, 20 mM Tris-HCl pH 7.5, 2 mM EDTA, 10% glycerol, and 1% Triton-X). The cleared lysates were bound to washed Protein A/G magnetic agarose beads (Thermo Scientific) and 1 μl of antibody (NEDD4-2, GSDMD/GSDME) and incubated at 4 °C overnight. The beads were washed twice in ice-cold lysis buffer, resuspended in 2× Laemmli sample buffer and immunoprecipitated proteins eluted by boiling for 5 min prior to immunoblot analysis. For ubiquitination assays, following transfection in HEK293T or THP-1 cells (WT and *NEDD4L* KO, for ubiquitination of endogenous GSDMD and GSDME) were treated with 20 μM MG132 (Boston Biochemical, Boston, MA, USA) and 400 μM chloroquine (Sigma-Aldrich) for 4 h prior to harvest to block proteasomal and lysosomal degradation. Cells were lysed in 2% SDS lysis buffer (2% SDS, 150 mM NaCl, 10 mM Tris-HCl, pH 8.0). Briefly, cell pellet were resuspended in the lysis buffer and the samples denatured by heating at 95 °C for 10 min. Samples were cooled on ice and diluted by adding dilution buffer (10 mM Tris-HCl, 150 mM NaCl, 2 mM EDTA, 1% Triton X-100, pH 8.0) to bring SDS concentration to 0.2% SDS. The samples were sonicated and left on ice for 20 min. Protein lysates were obtained by centrifuging at 13,000 rpm for 10 min at 4 °C. These were used in pull-down assays (anti-GFP following transfection or anti-GSDMD/anti-GSDME and anti-NEDD4L antibody for endogenous protein) to determine ubiquitination of transfected or endogenous proteins. Proteins were separated by electrophoresis using 4–20% precast polyacrylamide gels (Bio-Rad Laboratories, Hercules, CA, USA) and then transferred to polyvinylidene difluoride (PVDF) membrane using a Trans-blot turbo instrument (Bio-Rad). Membranes were blocked with 5% skim milk (1 h, RT) and immunoblotted with primary antibody diluted in 5% skim milk in TBS-T (Tris-buffered saline with 0.05% Tween 20) incubated overnight at 4 °C, followed by incubation with appropriate secondary antibody. For detection of HRP and Cy5 signals, images were acquired on a ChemiDoc Touch Imager (BioRad) whereas a Typhoon FLA biomolecular imager (GE Healthcare) was used to visualize signals from alkaline phosphatase. Quantitation was conducted using Image Lab Software (BioRad), with each band normalized to GAPDH or β-actin.

### Mouse lines and sample collection

All animal studies were approved by the institutional ethics and biosafety committees of the University of South Australia and were carried out according to the National Health and Medical Research Council of Australia guidelines. *Nedd4-2*-deficient mice (*Nedd4-2* KO), *Nedd4-2*-heterozygous mice (*Nedd4-2*^*+/−*^) and kidney-specific *Nedd4-2*-deficient mice (*Nedd4-2*^*Ksp1.3*^) were generated in our laboratory previously [20, 21] and bred at the University of South Australia core animal facility (Adelaide, Australia) under specific pathogen-free conditions. Mice Na^+^ diet feeding studies were done as reported previously [22]. Briefly, 8-week-old male *Nedd4-2*^*Ksp1.3*^ mice were fed standard sodium chow (0.2% Na^+^) or high sodium chow (3.1% Na^+^) (Specialty Feeds, WA, Australia) for 17 days. *Nedd4-2*^*+/−*^ mice were fed the same diets for 12 weeks. Mice were housed in metabolic cages for measuring metabolic parameters and blood pressure was measured by the tail-cuff method as reported before [22]. At the time of collection, mice were anesthetised, and blood and organs collected after cervical dislocation. Kidneys were decapsulated and the left kidneys were placed into Histochoice reagent (ProSciTech, Kirwan, QLD, Australia) for histological analysis of paraffin-embedded tissues. GFR was calculated based on creatinine clearance using a standard formula: GFR = urine flow × [urine creatinine]/[plasma creatinine] [21]. The right kidney was snap-frozen in liquid nitrogen for immunoblot or mRNA analysis. Serum and urine were analysed to obtain electrolyte levels and kidney function data (SA Pathology, Adelaide, Australia). Mouse IL-1β was measured in serum samples (mouse IL-1beta/IL-F2 ELISA kit # MLB00C, R & D Systems).

### Quantitative real-time PCR (qRT-PCR)

Total RNA was isolated from kidney using TRIzol (Life Sciences) and reverse transcription was performed using the High-Capacity cDNA reverse transcription kit (Applied Biosciences). The qRT-PCR reaction was set up, carried out and analysed as described previously [89]. For all reactions, *TBP* (*TATA box binding protein*) was used as a housekeeping gene and data normalised to TBP levels. Primers sequences are listed in Supplementary Table 1.

### Mouse tissue preparation and immunoblotting

For mouse samples, half of each kidney was lysed in ice-cold extraction buffer at pH 7.5 (50 mM Tris-HCl pH7.5, 1 mM EDTA, 1 mM EGTA, 0.27 M sucrose, 0.1% β-mercaptoethanol and HALT protease and phosphatase inhibitor cocktail (Thermo Fisher Scientific). Tissue was homogenised, frozen in liquid nitrogen, immediately thawed, and incubated at 4 °C on a nutator for 30 min and centrifuged at 13,000 rpm for 5 min to remove debris. 30–50 μg proteins were separated by electrophoresis using 4–20% precast polyacrylamide gels (Bio-Rad Laboratories, Hercules, CA, USA) and then transferred to polyvinylidene difluoride (PVDF) membrane using a Trans-blot turbo instrument (Bio-Rad). Blots were analysed with specific antibodies as mentioned above.

### Immunostaining

Mouse lung and kidney paraffin sections (5 μm) were deparaffinised and hydrated in graded ethanol series. Heat-mediated antigen retrieval was performed by heating sections at 95 °C for 10 min in 10 mM citric acid solution (pH 6). Tissue sections were blocked with 10% goat serum in PBS (1 h, 37 °C) in a humidified chamber followed by the addition of the primary antibodies anti-GSDMD (1:50, ab219800, Abcam), anti-cleaved GSDMD (1:50, 36425, Cell Signaling Technology), anti-GSDME (1:50, ab215191, Abcam), anti-F4/80 (1:100, MF48000, Invitrogen) and anti-Ly6G (1:100; 551459, BD Biosciences) overnight, 4 °C. Sections were washed three times (TBS-0.05% T20) and then incubated with the corresponding fluorescently tagged secondary antibodies (AlexaFluor-488, Thermo Fisher Scientific), counterstained with Hoechst 33342 (Thermo Fisher) and mounted in Prolong Gold Antifade reagent (Invitrogen). For immunostaining infected THP-1 cells, 24 h following infection, cells grown on coverslips were fixed in 4% paraformaldehyde for 15 min at room temperature and then permeabilised in 0.1% Triton X-100 in PBS for 10 min. After permeabilisation, cells were blocked in 1% BSA for 30 min at room temperature and then incubated overnight with primary antibody (anti-GFP, 1:500, 4 °C). Next day, cells were washed 3 times with TBS-T20 and incubated with anti-goat AlexaFluor-488 secondary antibody (1 h, 37 °C). Cell nuclei were stained with Hoechst 33342 (Thermo Fisher) and mounted using Prolong Gold Antifade reagent (Invitrogen). Images were acquired using a LSM 800 confocal microscope using Zen 2011 Black Edition (Carl Zeiss Microscopy, Jena, Germany). Image analysis was conducted using Adobe Image Suite software.

### Bacterial infection studies

The PMA differentiated THP-1 cells were seeded (1 × 10^6^) cells in DMEM containing 10% FBS without antibiotics. A single colony of *Salmonella enterica* serovar Typhimurium (*S*. Typhimurium) expressing GFP was picked up from a BHI plate and inoculated into a 5 ml BHI medium at 37 °C/180 rpm overnight. The overnight culture was sub-cultured into 50 ml BHI medium and grown until it reached OD600 value of 1.0 (stationary phase). The THP-1 cells were infected at MOI 10 for 10 min at room temperature followed by 30 min incubation at 37 °C. After 30 min, extracellular bacteria were removed and cells were then washed with medium containing 50 μg/ml of gentamicin (15750037, Life Technologies) and incubated in medium with 50 μg/ml of gentamicin. After 2 h, the concentration of gentamycin was reduced to 10 μg/ml. The infected cells were stained for GFP to visualise engulfed bacteria or collected for immunoblot analysis after 24 h as described above. All experiments with *S*. Typhimurium infection were handled in a Biosafety Level 2 laboratory and the protocol was approved by the University of South Australia Institutional Biosafety Committee (Reference No: IBC-B-032).

### Statistical analyses

Statistical analyses were performed using Graphpad Prism software (version 10, GraphPad Inc., La Jolla, CA, USA) or Microsoft Excel. Sample size for variable salt diet studies in heterozygous mice was calculated by G*Power software. Mice urine and serum samples were analysed by non-parametric Mann-Whitney test. Data was analysed by investigators not blinded to group identity. Two-way ANOVA was used to analyse caspase-3/7 positive THP-1 cells in a time-dependent manner. Nonlinear regression analysis using one phase decay was used to calculate the half-life of GSDMD and GSMDE. For confocal images, the mean fluorescence density of a protein (green) was measured and normalised to the number of cells (blue) in a tissue area of interest using Image J. All other analyses were performed using the two-tailed unpaired Student’s *t*-test. Data are expressed as mean ± standard error of mean (SEM) or mean ± standard deviation (SD), with *p* < 0.05 considered significant.

## Supplementary information

Supplementary Data

Uncropped Immunoblots

## Data Availability

All data generated or analysed during this study are included in this published article and its Supplementary Data files. Any data generated can be requested from the corresponding authors.
