# Correction: NEDD4L-mediated Gasdermin D and E ubiquitination regulates cell death and tissue injury

**DOI:** 10.1038/s41418-025-01653-x

**Published:** 2026-01-13

**Authors:** Sonia S. Shah, Jantina A. Manning, Yoon Lim, Diva Sinha, Ambika Mosale Venkatesh Murthy, Raja Ganesan, Nirmal Robinson, Emad S. Alnemri, Seth L. Masters, James E. Vince, Sharad Kumar

**Affiliations:** 1 Centre for Cancer Biology, University of South Australia, Adelaide, SA 5001 Australia; 2 Adelaide University, Adelaide, SA 5005 Australia; 3 University Hospital Cologne, TRIO Research Center, 50931 Köln, Germany; 4 Adelaide Medical School, The University of Adelaide, Adelaide, SA 5005 Australia; 5 Department of Biochemistry and Molecular Biology, Sidney Kimmel Cancer Center, Thomas Jefferson University, Philadelphia, PA 19107 USA; 6 The Walter and Eliza Hall Institute of Medical Research, Parkville, VIC 3052 Australia; 7 Centre for Innate Immunity and Infectious Diseases, Hudson Institute of Medical Research, Clayton, VIC 3168 Australia; 8 The Department of Medical Biology, University of Melbourne, Parkville, VIC 3010 Australia

**Keywords:** Proteolysis, Proteolysis

Correction to: *Cell Death & Differentiation* 10.1038/s41418-025-01598-1, published online 19 November 2025

During the proof correction process the final and revised fig. 3 has been mistakenly not included in the article.

**Incorrect Fig. 3**

**Figure:**
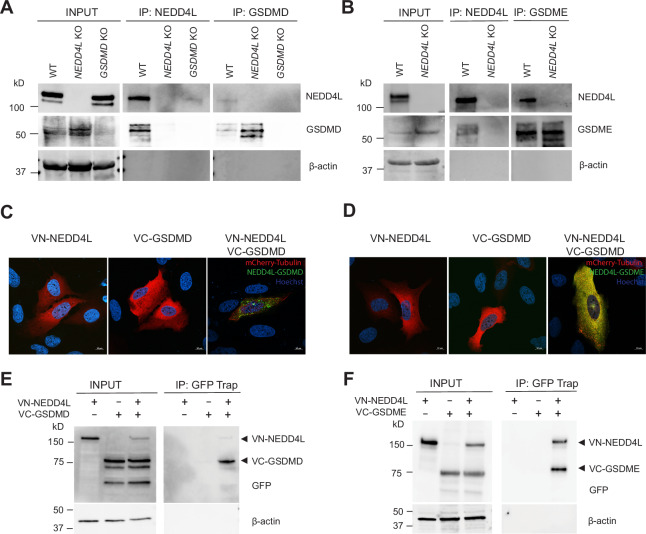

**Correct Fig. 3**

**Figure:**
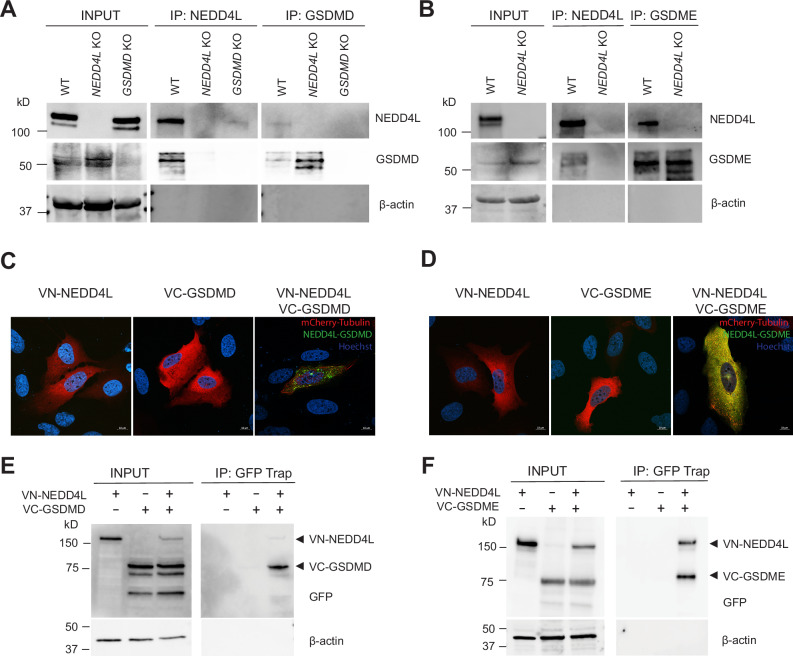

The original article has been corrected.

