# Supplementary material for: NEDD4L-mediated Gasdermin D and E ubiquitination regulates cell death and tissue injury
Source: Cell Death Differ. 2025 Nov 19;33(4):699–716. doi: 10.1038/s41418-025-01598-1 (PMC13076686; doi:10.1038/s41418-025-01598-1)

Legends for Supplementary Figures

Figure S1. (A) NEDD4L is predicted as an E3 for GSDMD and GSDME (DFNA5) by Ubibrowser 1.0. **(B and C)** Representative confocal images **(B)** and their quantitation **(C)** showing immunostaining for macrophage (F4/80, green) in lung and kidney of postnatal day 19 (P19) WT and *Nedd4-2* knockout (*Nedd4-2*$^{-/-}$) mice. DNA is stained with Hoechst (blue). Scale bar = 50 µm. **(D)** Schematic showing cleavage sites in GSDMD and GSMDE that can be inhibitory (✂) or cause activation (**✂**). NTD: N-terminal domain; CTD: C-terminal domain; C1: Caspase-1; C3: Caspase-3; C4: Caspase-4; C5: Caspase-5; C8: Caspase-8; C11: Caspase-11; GZMB: Granzyme B. **(E)** Immunoblots showing total and cleaved (cl) caspase 3 (CASP3) in kidney lysates from WT and *Nedd4-2*$^{Ksp1.3}$ mice fed standard (Std.) or high Na^+ diet. **(F)** qRT-PCR analysis shows no change in IL6 and TNFα expression in kidney tissue of WT and *Nedd4-2*$^{Ksp1.3}$ mice fed standard (Std.) or high Na^+ diet. **(G)** Immunoblots showing caspase 1 (CASP1) and NLRP3 expression in kidney lysates from WT and *Nedd4-2*$^{Ksp1.3}$ mice fed standard (Std.) or high Na^+ diet. GAPDH was used as loading control. Representative data from three independent experiments is shown. Data are Mean ± SEM (n = 3 mice/group). Statistical analysis was performed using two-tailed unpaired Student's t-test. **P < 0.01 and ***P < 0.001.

Figure S2. (A) qRT-PCR analysis for *GsdmD*, *GsdmE*, *Nlrp3* and *IL-1*β mRNA expression in kidney tissue of WT and *Nedd4-2*$^{Ksp1.3}$ mice fed standard (Std.) or high Na^+ diet. **(B and C)** Immunostaining (green) for F4/80 (**B**) and Ly6G (**C**) in mice kidney reveal increased infiltration in *Nedd4-2*$^{Ksp1.3}$ mice kidneys on high Na^+ diet. DNA is stained with Hoechst (blue). Scale bar = 50 µm. Representative images from three independent experiments are shown. **(D)**

Quantitation of immunostaining for F4/80 and Ly6G positive cells. Data are Mean ± SEM (at least n = 3 mice/group). Statistical analysis was performed using two-tailed unpaired Student's t-test. *P < 0.05 and **P < 0.01.

Figure S3. (A) Immunoblots showing generation of *NEDD4L* KO (THP-1) clones (#5, #6, #7). Clones #5 and #6 were used for cell death studies. *GSDMD* KO (GD KO) cells express NEDD4L. **(B and C)** Immunoblots and **(D)** their quantitation to show expression of GSDMD and GSDME proteins in A549 and U2OS cells after *NEDD4L* knockdown using two different siRNA (#1 and #2). siRNA sequences are provided in Supp. Table 2. Representative data from three independent experiments are shown. Statistical analysis was performed using two-tailed unpaired Student's t-test. *P < 0.05 and **P < 0.01.

Figure S4. (A) Diagram showing high salt diet feeding plan. **(B)** Data from WT and *Nedd4-2* heterozygous mice (*Nedd4-2*$^{+/-}$) fed high salt diet for 3 months. No difference in systolic blood pressure was observed. GFR, water intake and urine produced were measured using metabolic cages. Kidney injury marker (*Kim1*) levels were determined by qRT-PCR. PRE: before the start of high salt diet, POST: after 3 months high salt diet. **(C)** Immunoblots of kidney lysates from high Na^+ fed mice and their quantitation showing elevated GSDMD and GSDME expression in the *Nedd4-2*$^{+/-}$ mice. **(D)** Immunoblots from BMDMs from high salt fed mice show activation of both proteins in the *Nedd4-2*$^{+/-}$ mice. Blots for two WT and three heterozygous (*Nedd4-2*$^{+/-}$) mice are shown. Statistical analysis was performed using a two-tailed unpaired Student's t-test (Mean ± SEM). For blood pressure measurements, significance was calculated using one

way ANOVA for repeated measurements (Mean ± SEM, n=6,10). *P < 0.05, ***P < 0.001 and ns: non-significant.

Figure S5. Immunoblots showing ubiquitination of GSDMD and GSDME. **(A and B)** HEK293T cells were co-transfected with Ub-HA, NEDD4L-MYC and GSDMD-GFP or GSDME-GFP expression constructs containing either full-length (FL), NTD (N-terminal domain), or CTD (C-terminal domain) of each protein to identify the region that is ubiquitinated by NEDD4L. Transfection of NTD of GSDMD caused significant cell death and NTD was difficult to detect by immunoblotting. Representative blots and images from three independent experiments are shown. **(C and D)** Prediction of ubiquitination sites using BDM-PUB for **(C)** GSDMD and **(D)** GSDME.

Figure S6. (A) Immunoblots from WT, *NEDD4L* KO and *GSDMD* KO THP-1 cells that were primed with LPS and treated with nigericin (5 µM, 1 h), ATP (2.5 mM, 1 h) or MSU (500 µg/ml, 5 h) showing increased GSDMD and GSDME activation and release of mature IL-1β in the supernatant fraction of *Nedd4l* KO cells. *GSDMD* KO cells were used as control. (**B**) Representative images and (**C**) Histogram showing cell death in THP-1 cells treated with LPS and nigericin. THP-1 cells (WT and *NEDD4L* KO) were differentiated using PMA and primed with LPS, NucLight Casp-3/7 green dye was added (1:2000) and cells treated with nigericin (5 µM). Data were collected at 30 min intervals and presented as fraction of dead (Casp-3/7 green dye positive) cells. Images shown were obtained at the end of experiment Scale bar =

50µm. Representative blots and images from three independent experiments are shown. Statistical analysis was performed using 2-way ANOVA analysis in **C**. ***P < 0.001.

Figure S7 (A) Cloning strategy for the generation of pINDUCER20-MYC-NEDD4-2. Schematic was prepared using SnapGene. **(B)** Immunoblots showing detection of NEDD4-2-MYC after dox induction. Representative blots from three independent experiments are shown.

Supplementary Table 1: Sequence of primers pairs used in the study

Gene	Forward	Reverse
mGsdmD-qPCR	5'-GCGATCTCATTCCGGTGGACA-3'	5'-TTCCCATCGACGACATCAGAGAC-3'
mGsdmE-qPCR	5'-ACCCCCTGGCCTACAGAGAG-3'	5'-ACCGCAAAGGGATGGAAACT-3'
IL-1β-qPCR	5'-CCAGAGATACAAAGAAATGATGG-3'	5'-ACTCCAGAAGACCAGAGGAAAT-3'
Nlrp3-qPCR	5'-AGAGCCTACAGTTGGGTGAAATG-3'	5'-CCACGCCTACCAGGAAATCTC-3'
IL6-qPCR	5'-CAGCATGGCTGATGTTAAGTTTTC-3'	5'-CCCAGGCAGGCGCATAC-3'
TNFα-qPCR	5'-AGGTCCCTACAGGGAACAAA-3'	5'-TCTCTCTTTCCTTAGACACACGA-3'
Kim1-qPCR	5'-TGGTTGCCTTCCGTGTCTCT-3'	5'-TCAGCTCGGGAATGCACAA-3'
TBP-qPCR	5'-CAAACCCAGAATTGTTCTCCTT-3'	5'-ATGTGGTCTTCCTGAATCCCT-3'
C-GSDMD-GFP	5'-AAAGAATTCGCCACCATGGGGGTC CCTGCGGAGGGG-3'	5'-AAAGGATCCGCGTGGGGCTCC TGGCTCAGTCC-3'
C-GSDME-GFP	5'-CCCCTCGAGGCTGCGCATGGGATATCT-3'	5'-CGCGGATCCTCATGAATGTTCTCTGCCTAA-3'
MYC-NEDD4L	5'-AAAGATCTCGGAGCGACCCTATACATTTAAG-3'	5'-AAGGTACCTTAATCCACCCCTTCAAATCC-3'
VC-GSDMD	5'-AAAAGATCTTT GGGTCGGCCTTTGAGCGG -3'	5'-AAAGGTACCGTGGGGCTCCTGGCTCAG-3'
VC-GSDME	5'-AAAAGATCTTTTTTGCCAAAGCAACCAGGAATTTTC-3'	5'-AAAGGTACCTGAATGTTCTCTGCCTAAAGCAC-3'
VN-NEDD4L	5'-AAAAGATCTtGCGACCGGGCTCGGGGAG-3'	5'-AAAAGATCTTGCGACCGGGCTCGGGGAG-3'
GSDMD K51R	5'-TCATGGTTCTGGAGACCCCGTTATAAG-3'	5'-CTTATAACGGGGTCTCCAGAACCATGA-3'
GSDMD K103R	5'-CCAGGACAGGCAAGGATCGCAGGC-3'	5'-GCCTGCGATCCTTGCCTGTCCTGG-3'
GSDMD K145R	5'-CAGCCAGAACACAGAGTCCTGCAGCAGCT-3'	5'-AGCTGCTGCAGGACTCTGTGTTCTGGCT-3'G
GSDMD K177R	5'-ACGCGCACCCACAGGCGGGAGGGCTC-3'	5'-GAGCCCTCCCGCCTGTGGGTGCGCGT-3'
GSDMD K203R	5'-CATCTGAGCCAGAGGAAGACGGTCA-3'	5'-TGACCGTCTTCCTCTGGCTCAGATG-3'
GSDMD K204R	5'-TGAGCCAGAAGAGGACGGTCACCATCC-3'	5'-GGATGGTGACCGTCCTCTTCTGGCTCA-3'
GSDMD K235R	5'-CTCTTCCCGGATAGGAAGCAGAGGACCT-3'	5'-AGGTCCTCTGCTTCCTATCCGGGAAGAG-3'
GSDMD K236R	5'-CTCTTCCCGGATAAGAGGCAGAGGACCT-3'	5'-AGGTCCTCTGCCTCTTATCCGGGAAGAG-3'
GSDMD K248R	5'- GCGACAGGCCACAGGCGTTCCACGA-3'	5'- TCGTGGAACGCCTGTGGCCTGTCGC-3'
GSDMD K299R	5'- GAGACCATCTCCAGGGAACTGGAGCT-3'	5'- AGCTCCAGTTCCCTGGAGATGGTCTC-3'
GSDME K4R	5'- ATGTTTGCCAGAGCAACCAGGAATTTTC-3'	5'- GAAAATTCCTGGTTGCTCTGGCAAACAT-3'
GSDME K39R	5'- CTGGTGACAAGAAAGAAGAGATTCTGG-3'	5'- CCAGAATCTCTTCTTTCTTGTCACCAG-3'
GSDME K40R	5'-CTGGTGACAAAAAGGAAGAGATTCTGG-3'	5'-CCAGAATCTCTTCCTTTTTGTCACCAG-3'
GSDME K41R	5'-CTG GTG ACA AAA AAG AGG AGA TTC TGG	5'-CCA GAA TCT CCT CTT TTT TGT CAC CAG-3'
GSDME K98R	5'-GCACTGGGGAGGGTCAAGCTGAACCTG-3'	5'-CAGGTTCAGCTTGACCCTCCCCAGTGC-3'
GSDME K100R	5'-GCACTGGGGAAGGTCAGGCTGAACCTG-3'	5'-CAGGTTCAGCCTGACCTTCCCCAGTGC-3'
GSDME K120R	5'-GGAACCCTGAGGAGGCAGGAGGTG-3'	5'-CACCTCCTGCCTCCTCAGGGTTCC-3'
GSDME K240R	5'-CTTCTCCGAGGGAGGCAAGGTGGCTTC-3'	5'-GAAGCCACCTTGCCTCCCTCGGAGAAG-3'
GSDME K440R	5'-ACTCCCCTGAGAGATACAGAAAGG-3'	5'-CCTTTCTGTATCTCTCAGGGGAGT-3'
GSDME K467R	5'-GCTGTCATTCTGAGGGACTCTAAAGTC-3'	5'-GACTTTAGAGTCCCTCAGAATGACAGC-3'
GSDME K472R	5'-CTGAAGTCATCTGTGAGAGCTGTCATTCTG-3'	5'-CAGAATGACAGCTCTCACAGATGACTTCAG-3'
si*Ctrl*	5'-UUCUCCGAACGUGUCACGUTT-3'	5'-ACGUGACACGUUCGGAGAATT-3'
si*NEDD4L*#1	5'-AACCACAACACAAAGUCACACTT-3'	5'-GUGUGACUUUGUGUUGUGGUUTT-3'
si*NEDD4L*#2	5'-GGAGACAGCAUUCUAUUUATT-3'	5'-UAAAUAGAAUGCUGUCUCCTT-3'

Supp. Figure 1

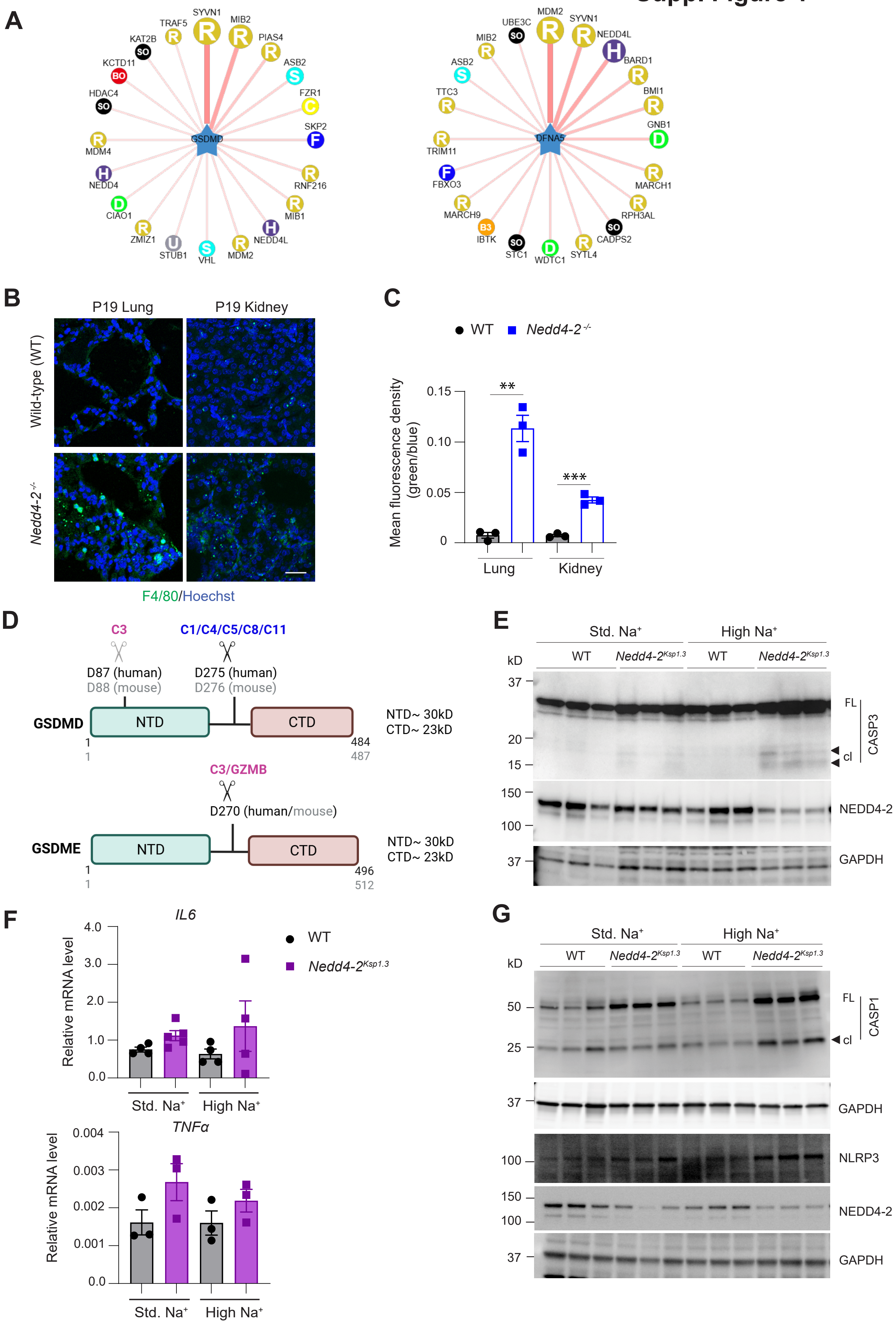

Supp. Figure 2

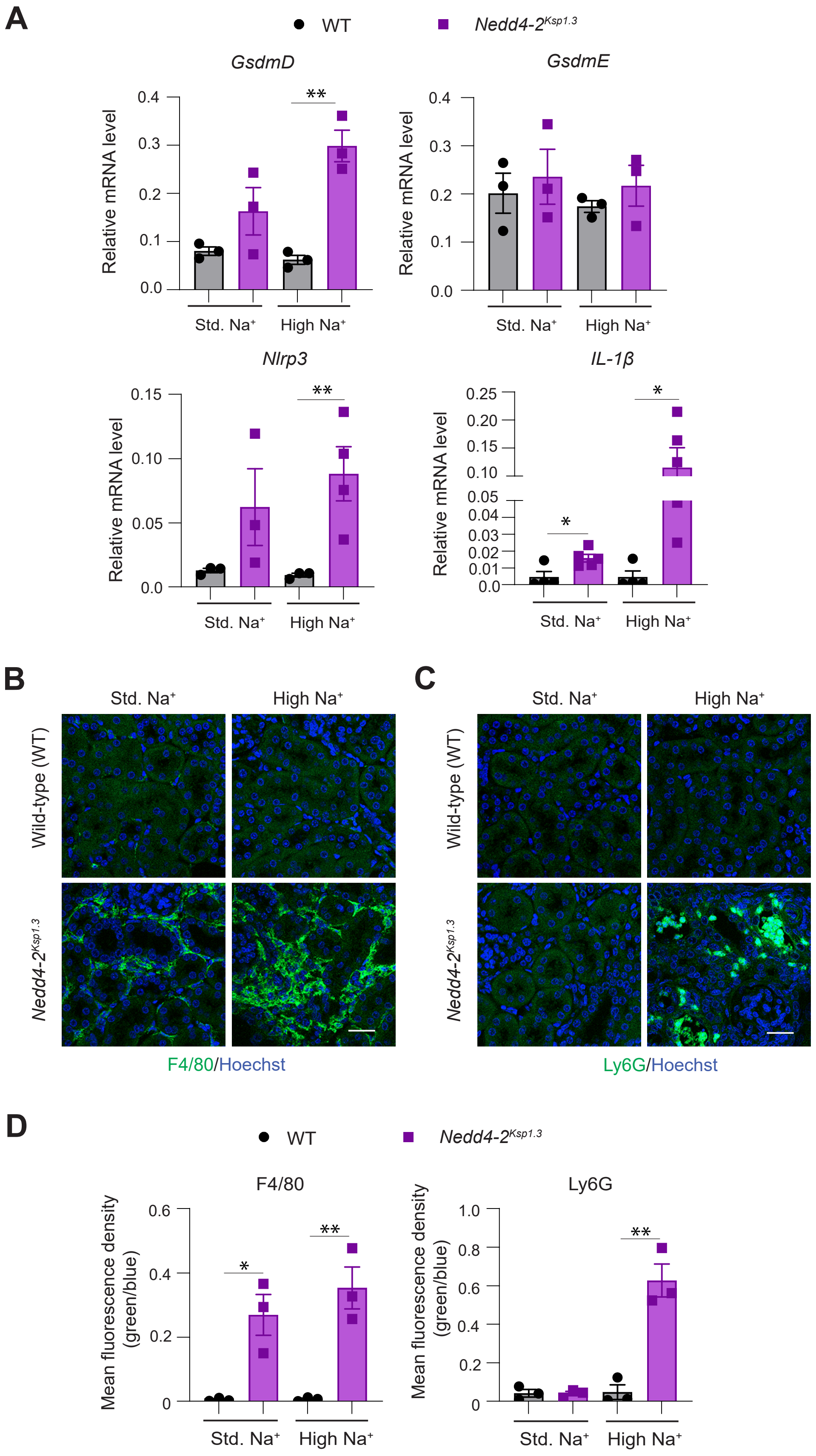

Supp. Figure 3

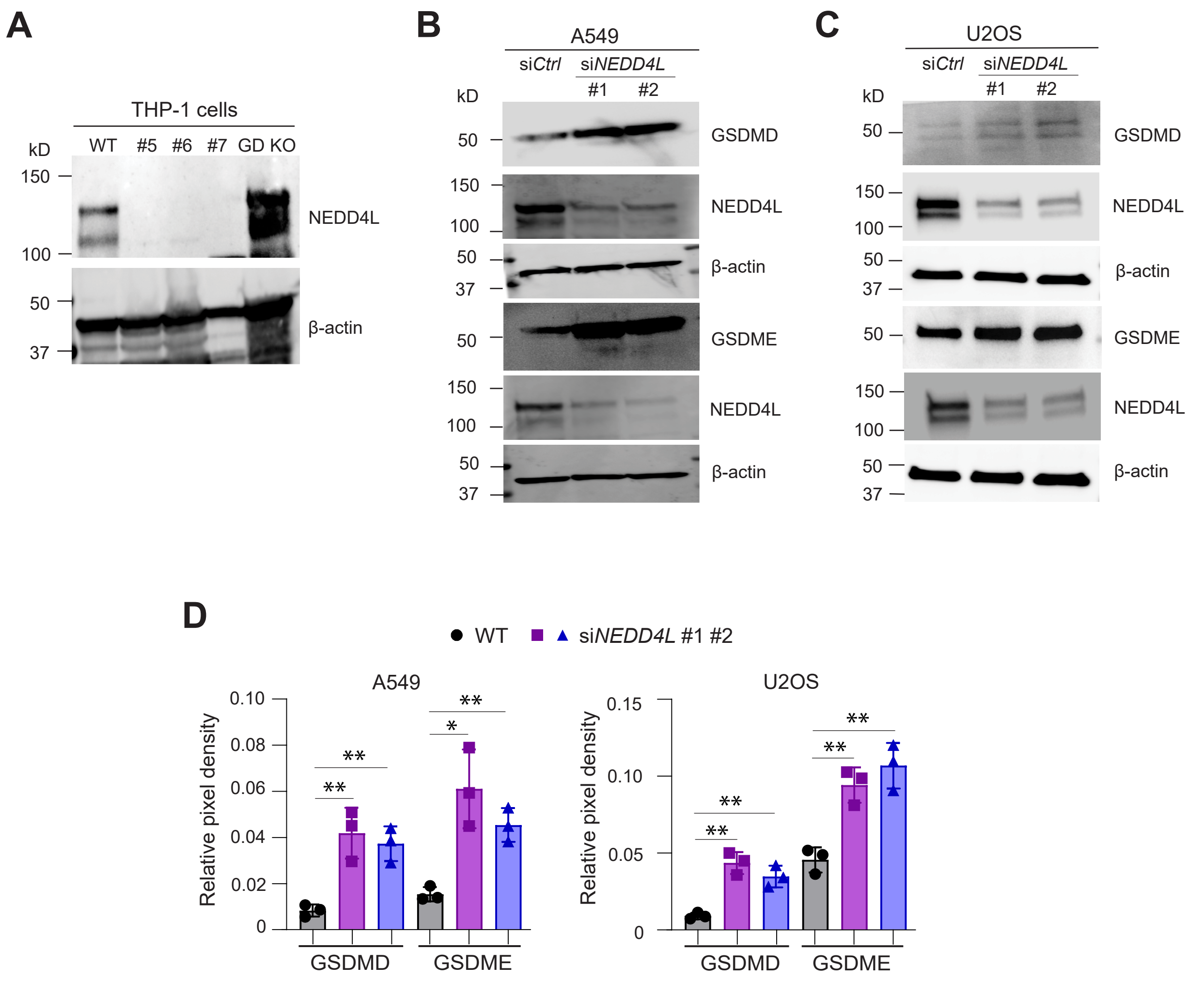

Supp. Figure 4

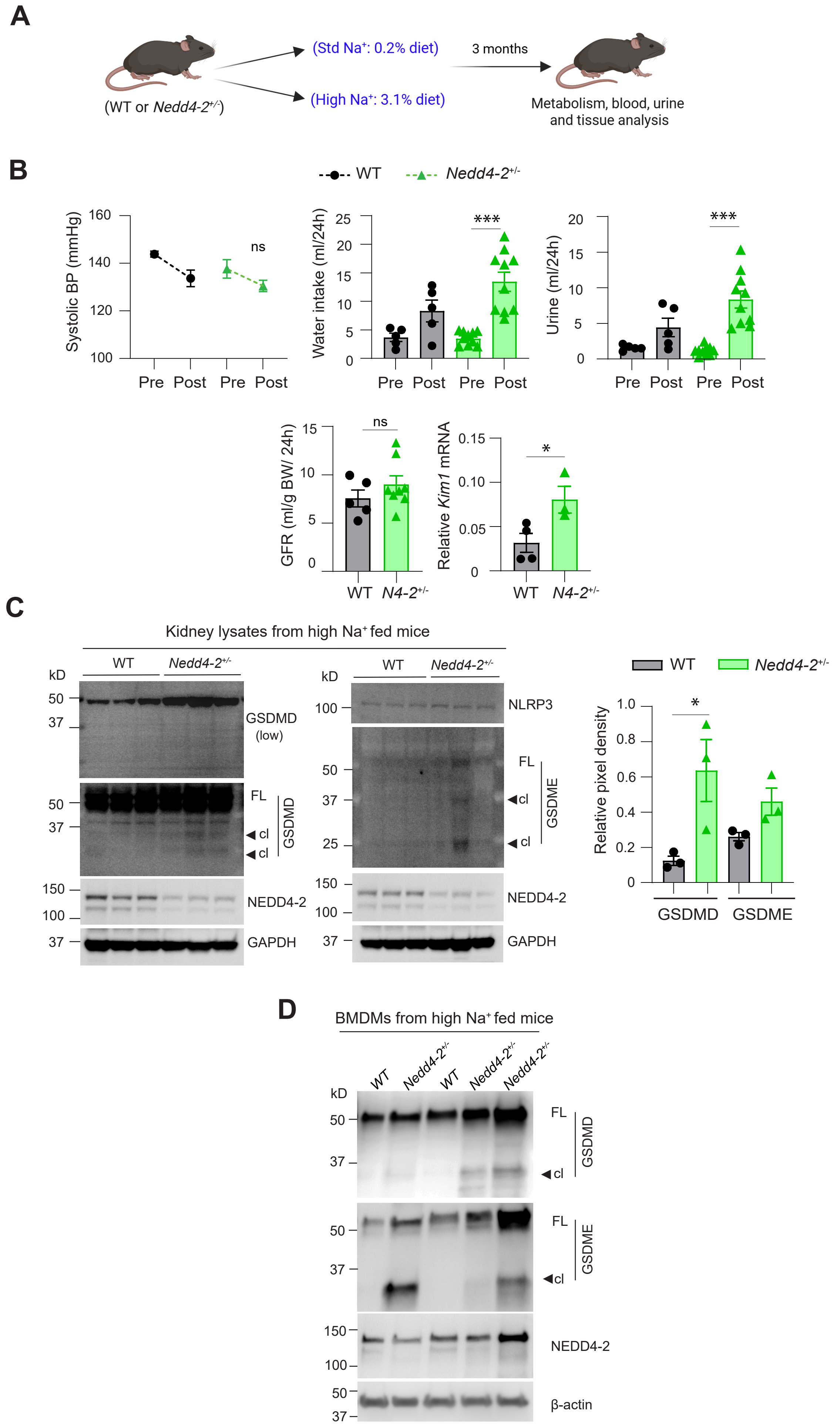

Supp. Figure 5

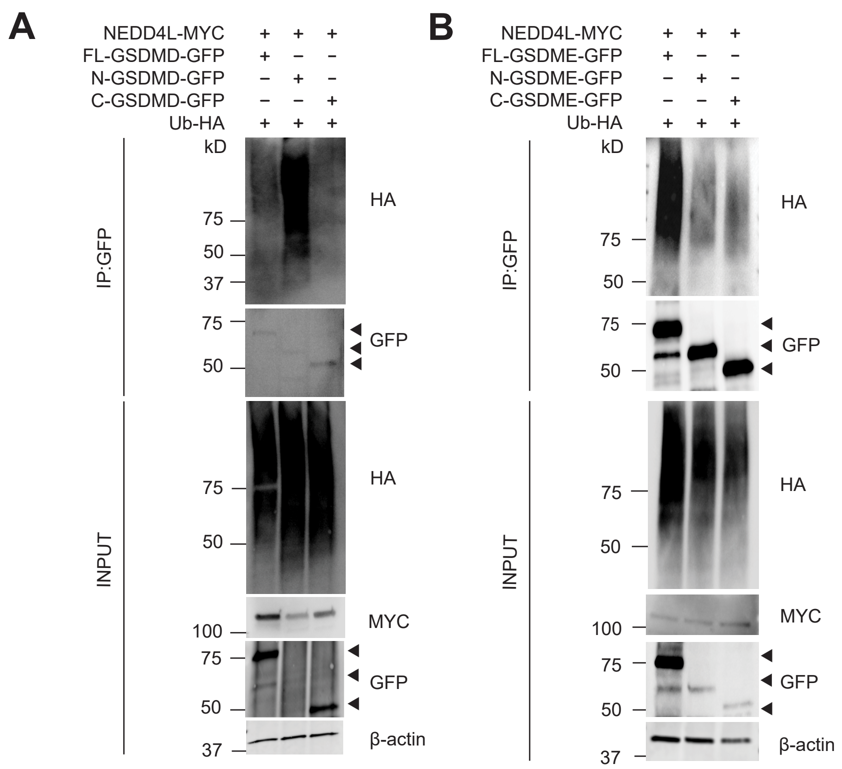

C

※ **BDM-PUB:** Prediction of **Ub**iquitination sites with **B**ayesian **D**iscriminant **M**ethod

Go back to BDM-PUB prediction page

Predicted Ubiquitination sites:

Peptide	Position	Score	Threshold
PSSSWFWKPRYKCVN	51	0.63	0.3
LAAPGQAKIAGGAAV	103	1.69	0.3
HLRQPEHKVLQQLRS	145	1.13	0.3
VEVTRTHKREGSGRF	177	1.02	0.3
GQGHLSQKKTVTIPS	203	1.18	0.3
QGHLSQKKTVTIPSG	204	1.61	0.3
QPPATGHKRSTSEGA	248	3.57	0.3

Download the TAB-deliminated data file from here.

Go back to BDM-PUB prediction page

D

※ **BDM-PUB:** Prediction of **Ub**iquitination sites with **B**ayesian **D**iscriminant **M**ethod

Go back to BDM-PUB prediction page

Predicted Ubiquitination sites:

Peptide	Position	Score	Threshold
****MFAKATRNFLR	4	2.73	0.3
DFVKYEGKFANHVSG	83	0.39	0.3
TLETALGKVKLNLGG	98	1.65	0.3
ETALGKVKLNLGGSS	100	1.87	0.3
SSFGTLRKQEVDLQQ	120	0.84	0.3
GIVGIQTKTVQVSAT	189	0.57	0.3
EFCLLRGKQGGFENK	240	1.61	0.3
QGGFENKKRIDSVYL	248	0.45	0.3
DGPLSVLKQATLLLE	286	1.96	0.3
GPEDAGSKQLFMTAY	380	2.52	0.3
ALLGTCCKLQIIPTL	409	0.51	0.3
DPTLTPLKDTERFGI	440	0.71	0.3
DISLERLKSSVKAVI	463	1.33	0.3
ERLKSSVKAVILKDS	467	2.35	0.3
SVKAVILKDSKVFPL	472	0.56	0.3
AVILKDSKVFPLLLC	475	1.40	0.3

Download the TAB-deliminated data file from here.

Supp. Figure 6

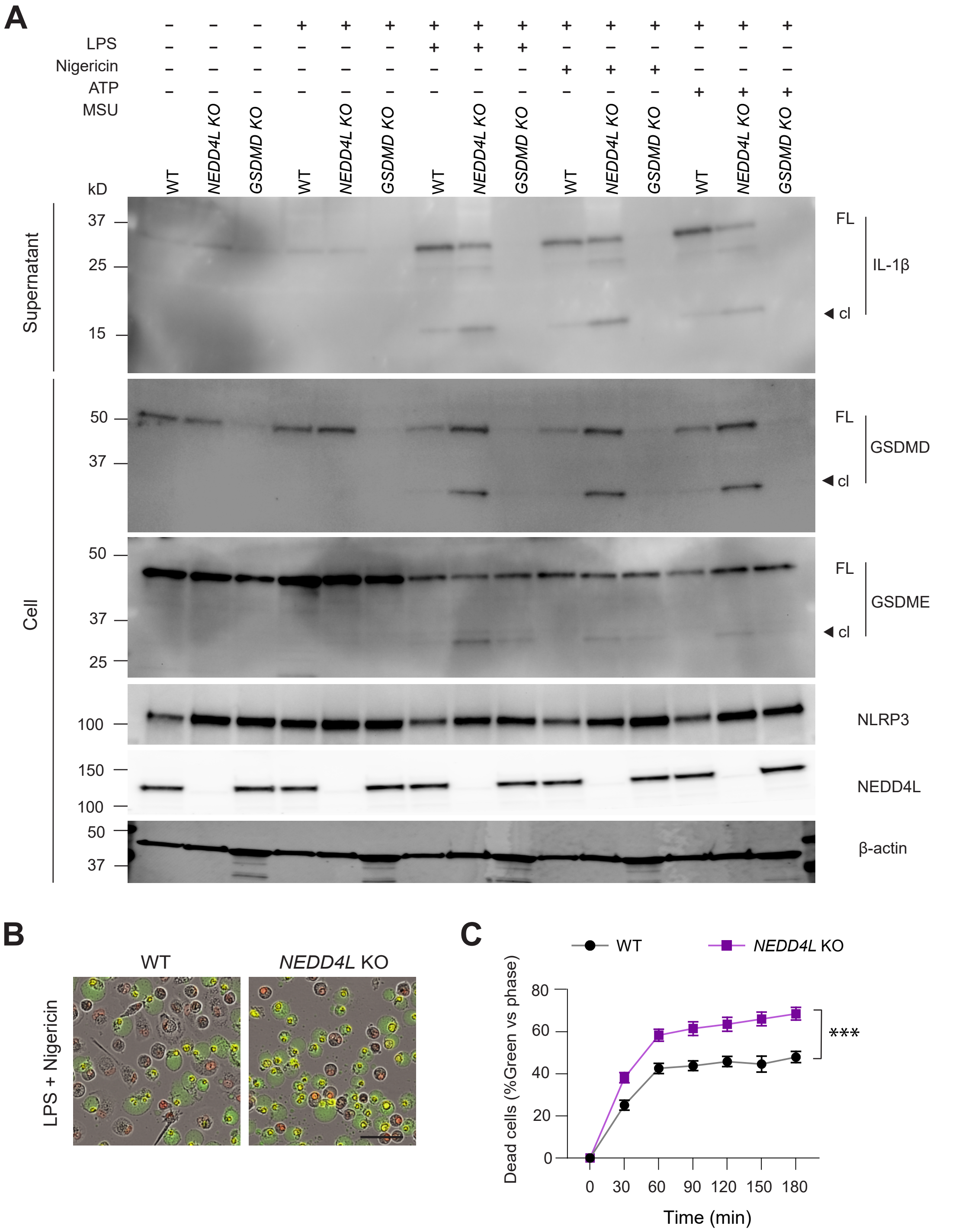

Supp. Figure 7

A

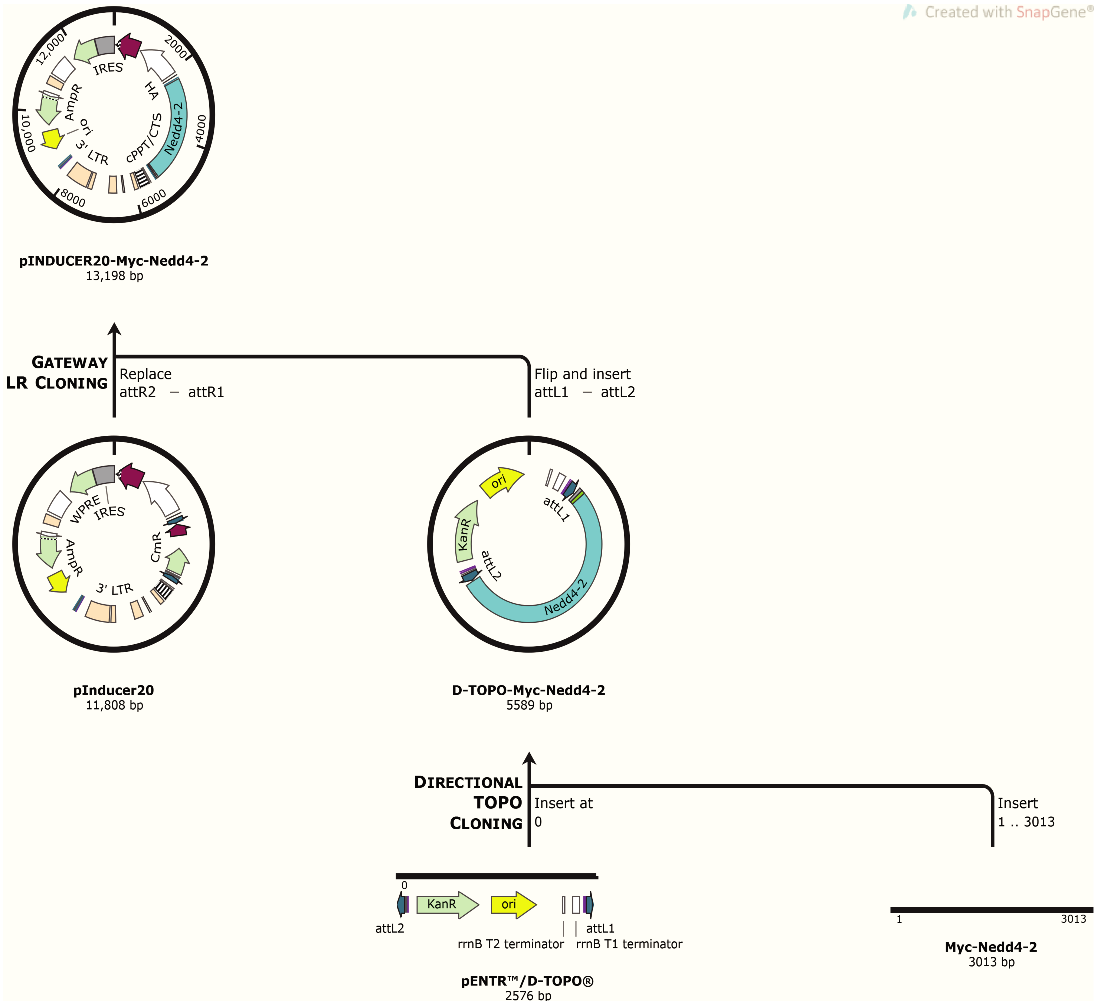

B

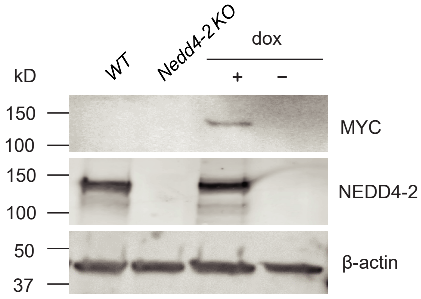

Supplement: Supplementary file 1 — Supplementary Data [file 41418_2025_1598_MOESM1_ESM.pdf]
