# Supplementary material for: NEDD4L-mediated Gasdermin D and E ubiquitination regulates cell death and tissue injury
Source: Cell Death Differ. 2025 Nov 19;33(4):699–716. doi: 10.1038/s41418-025-01598-1 (PMC13076686; doi:10.1038/s41418-025-01598-1)
Supplement: Supplementary file 2 — Uncropped Immunoblots [file 41418_2025_1598_MOESM2_ESM.pdf]

Figure1F, 1G

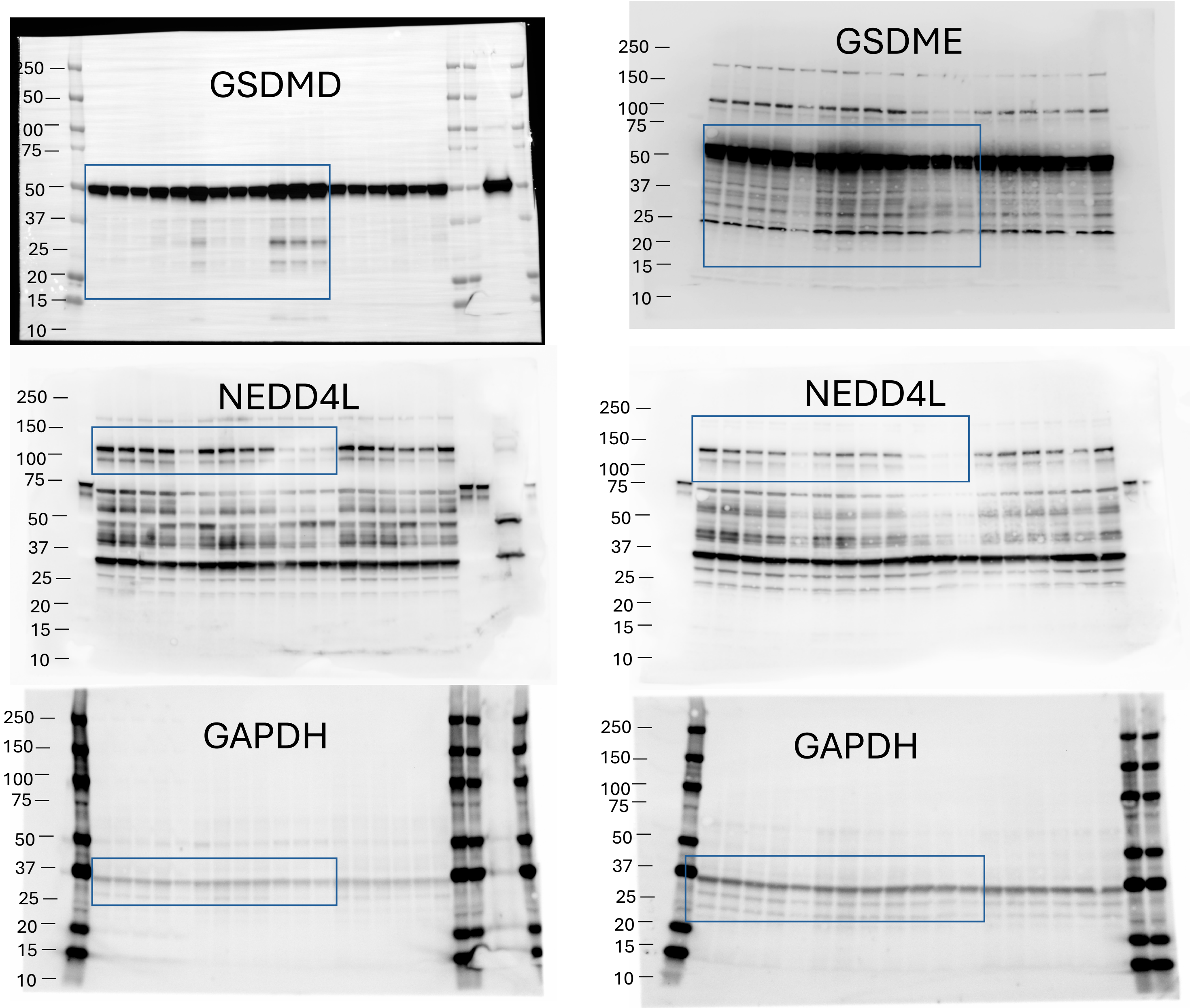

Figure 2A

THP-1

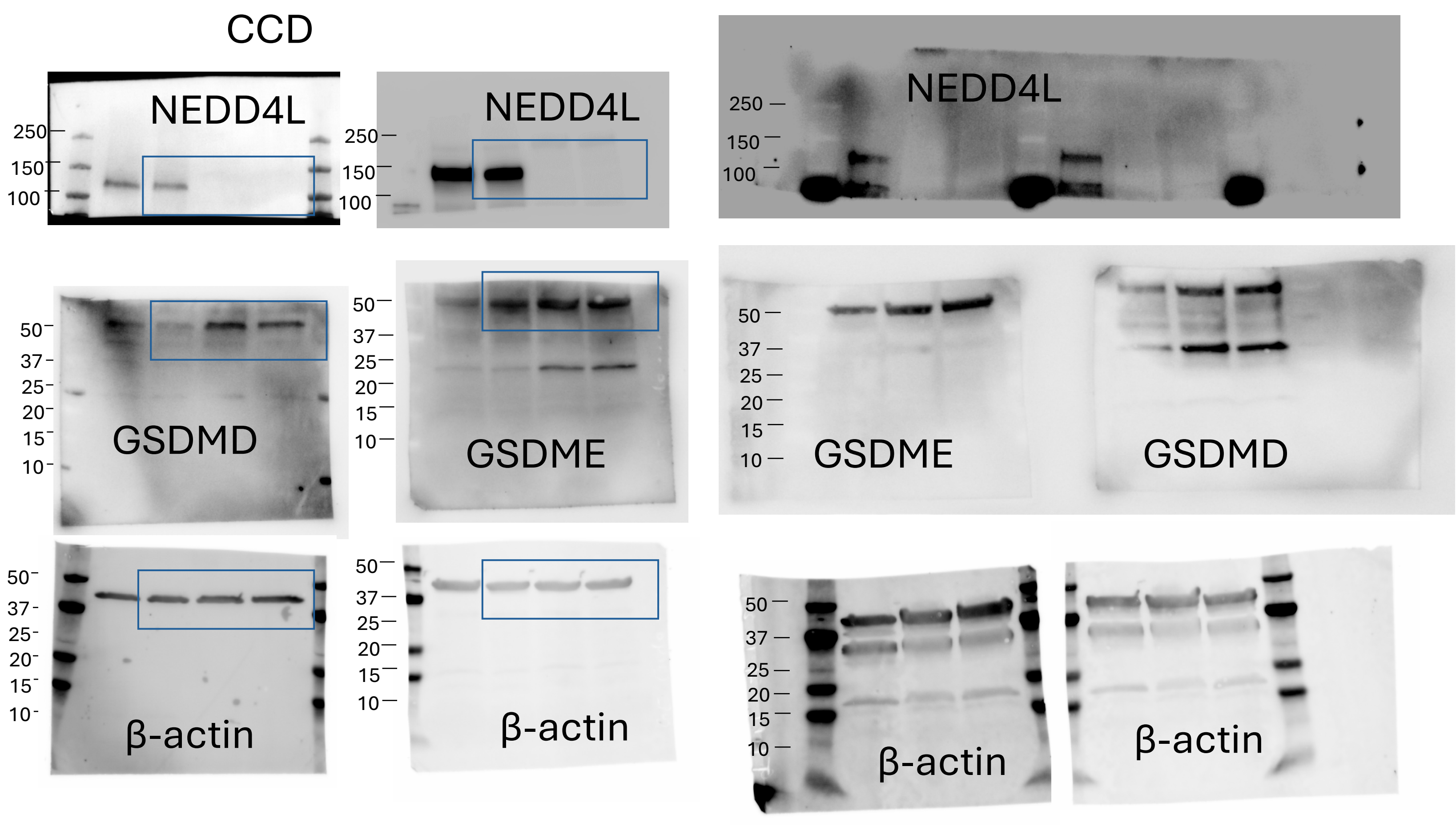

Figure 2C

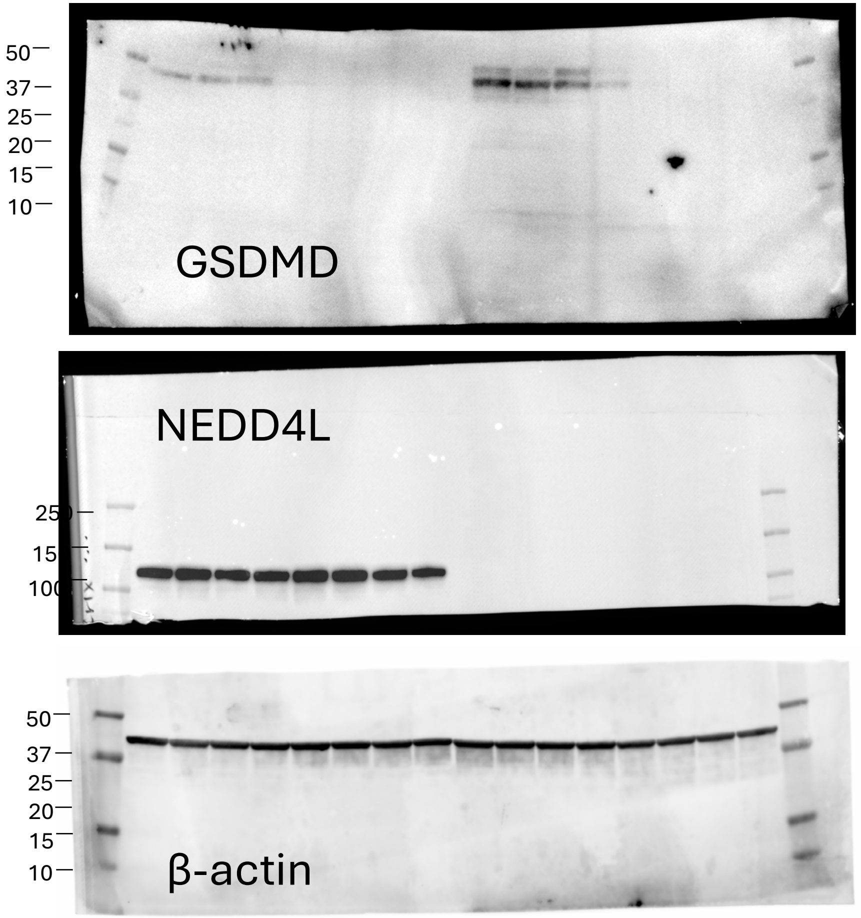

Figure 2D

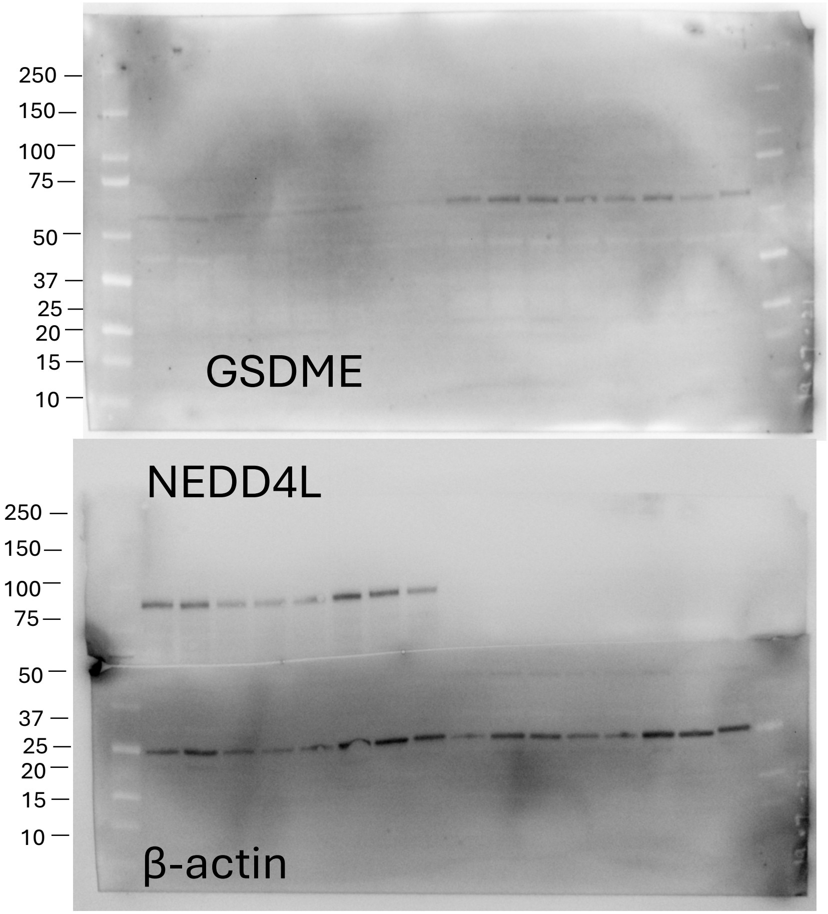

Figure 3A

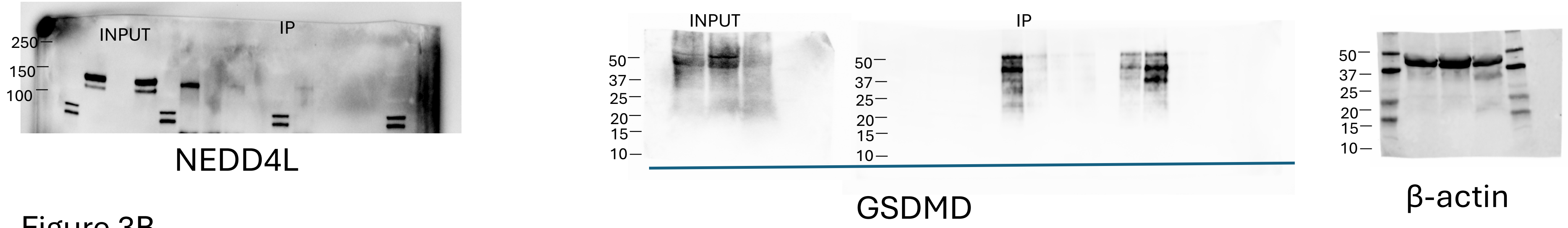

Figure 3B

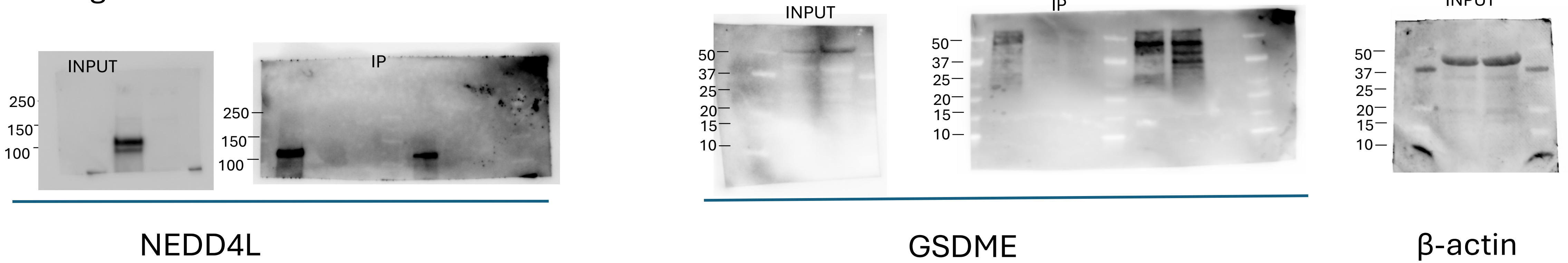

Figure 3E

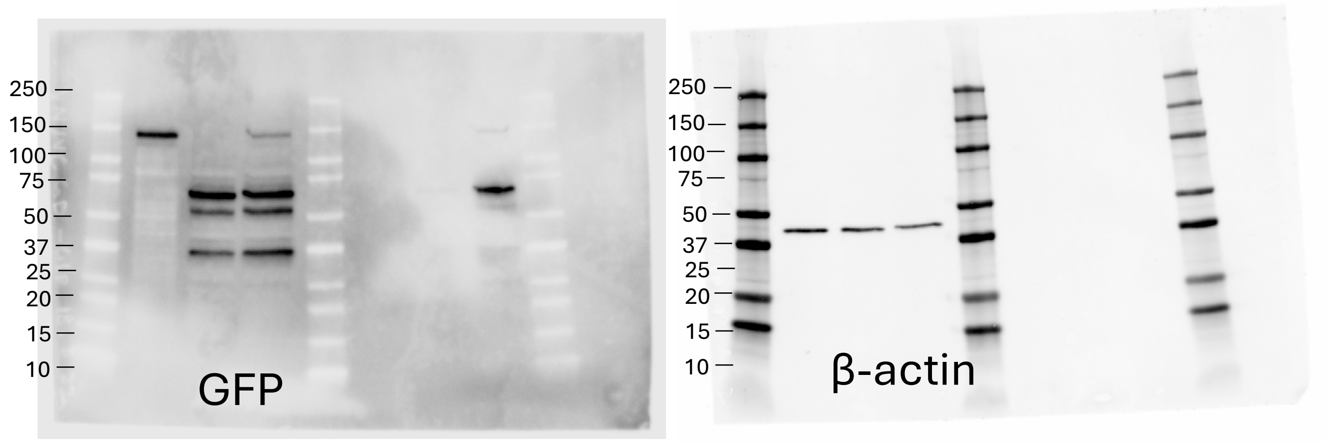

Figure 3F

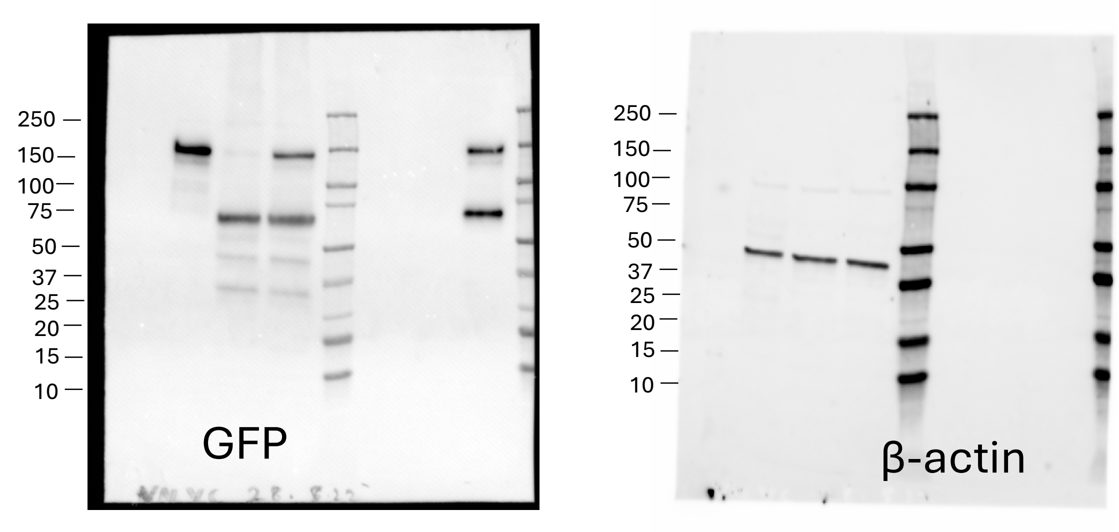

Figure 4A

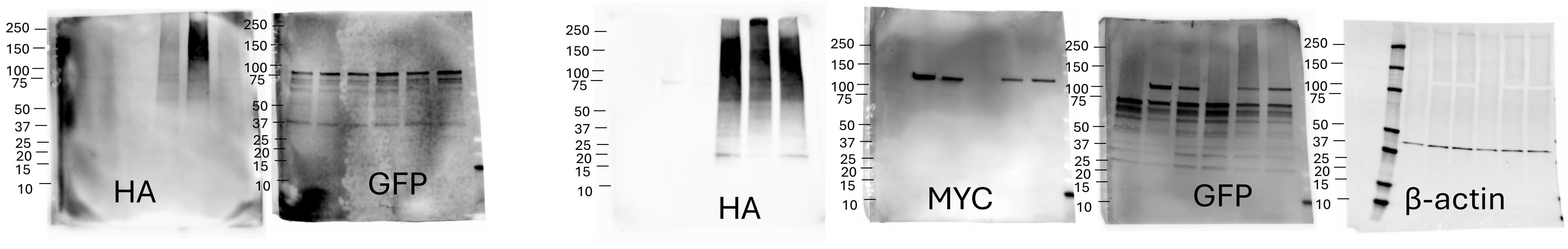

Figure 4B

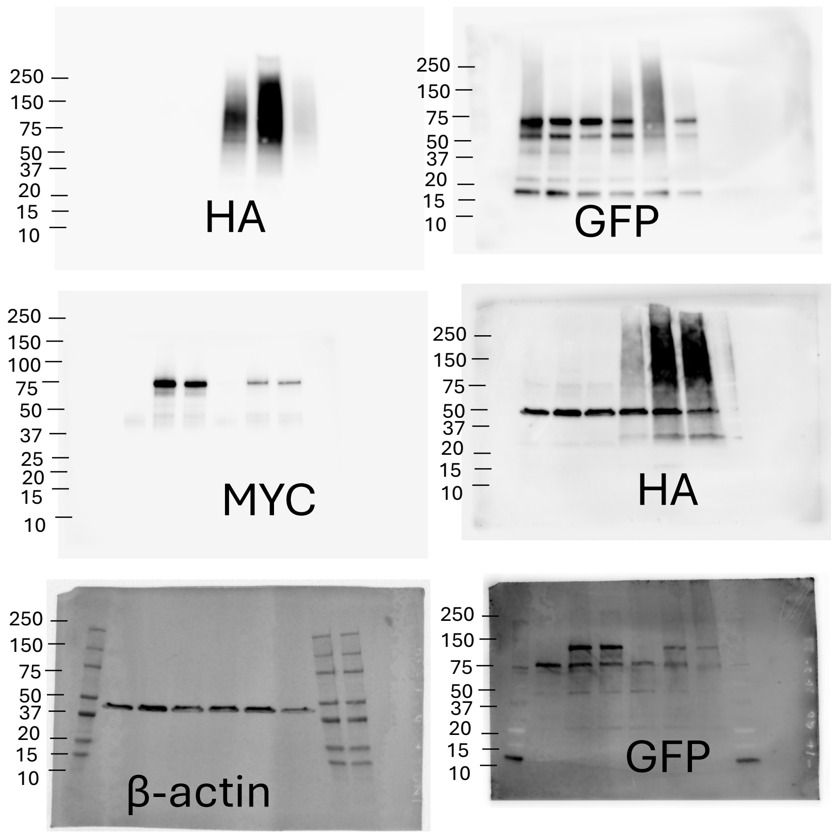

Figure 4C : GSDMD

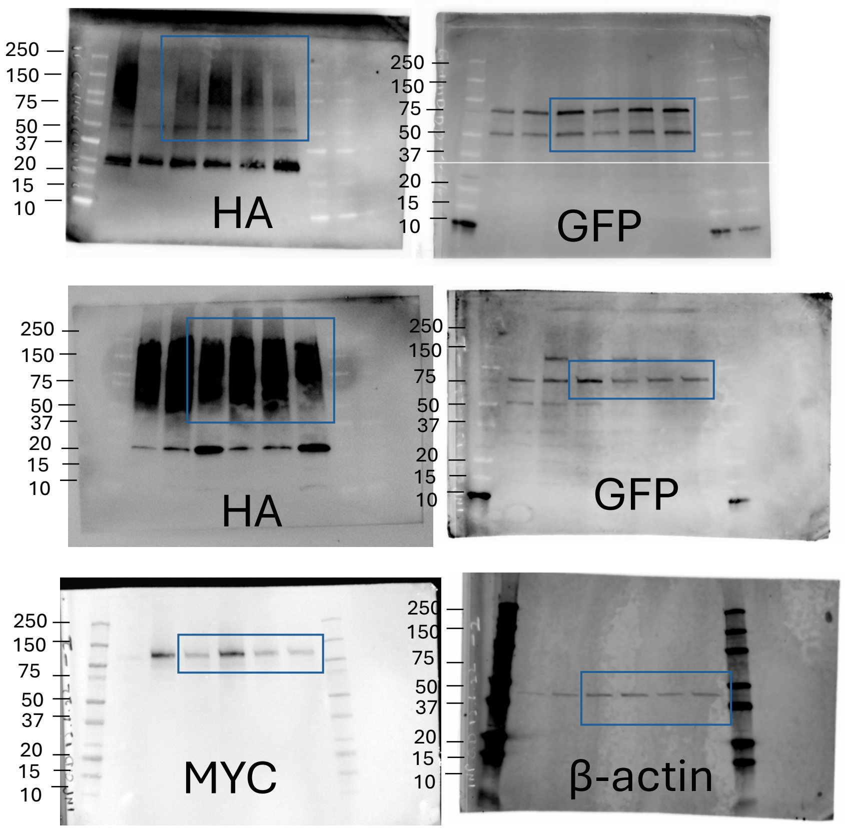

Figure 4C: GSDME

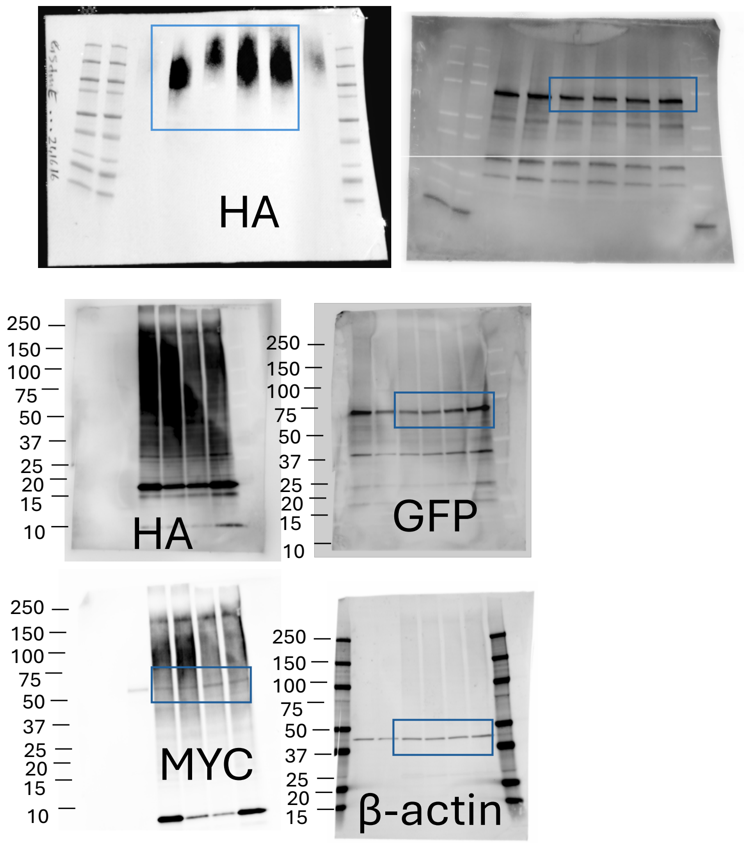

Figure 4D:GSDMD

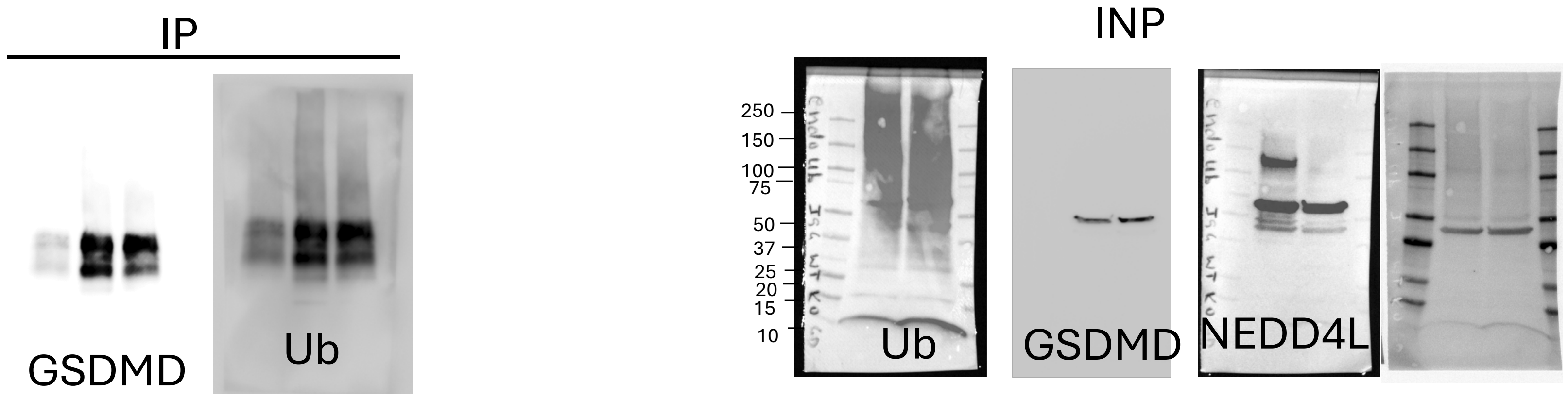

Figure 4D:GSDME

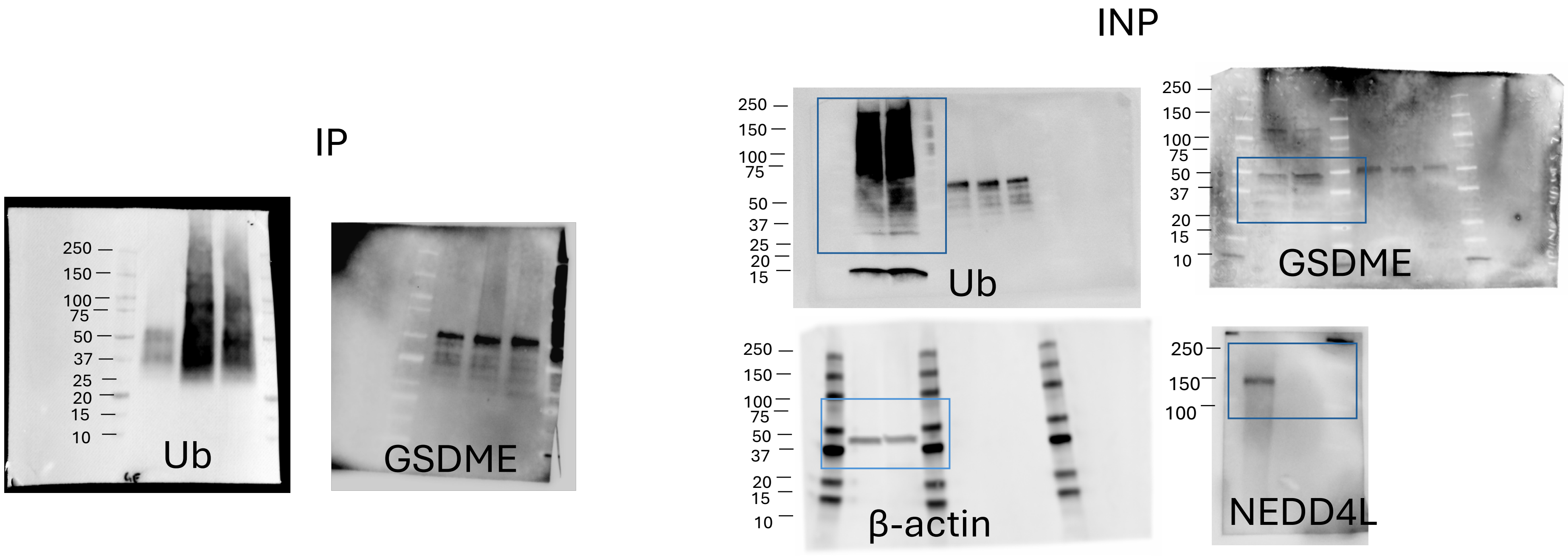

Figure 4E:GSDMD

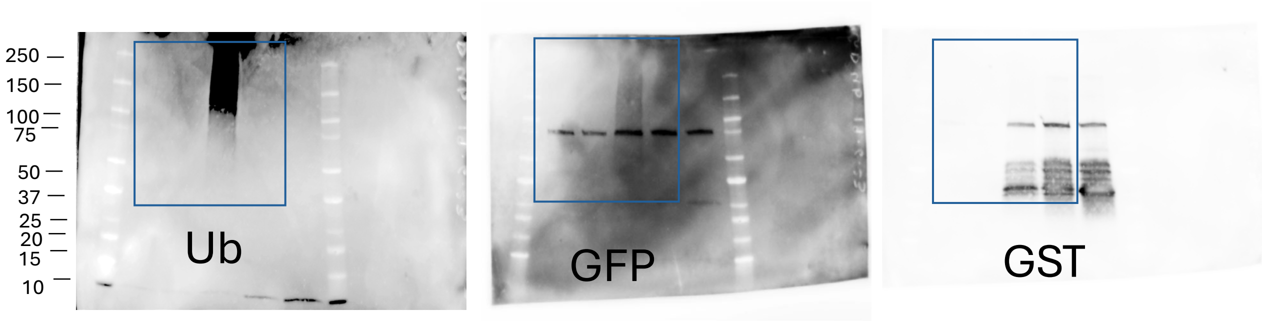

Figure 4E:GSDME

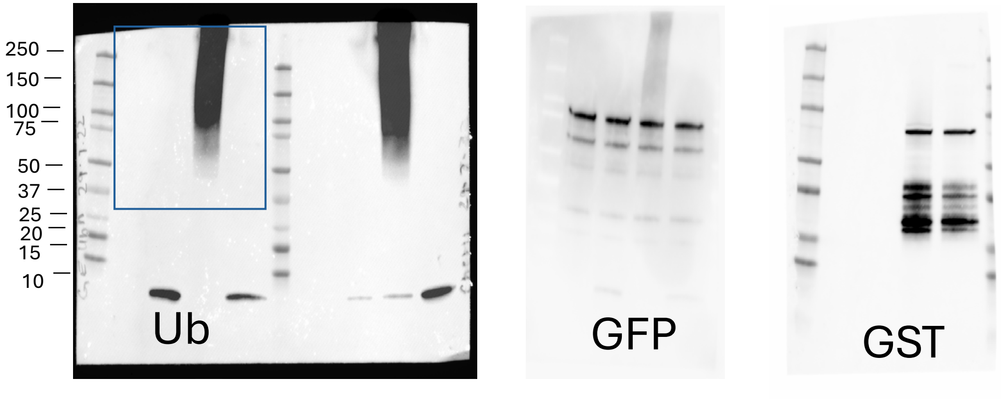

Figure 4E:GFP

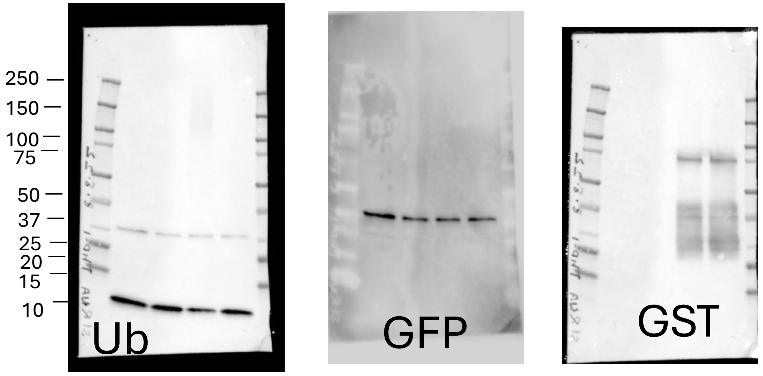

Figure 5A

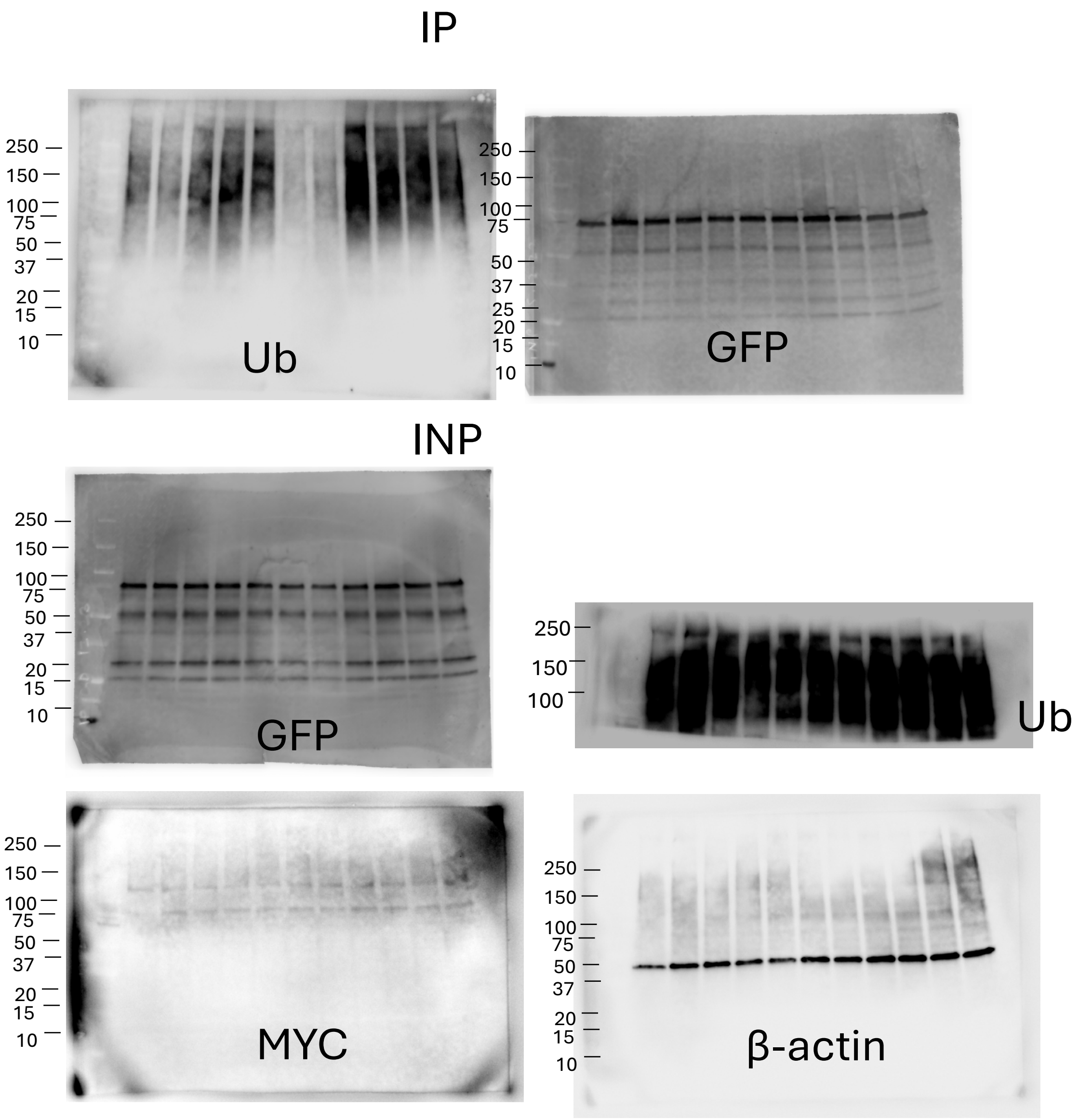

Figure 5B

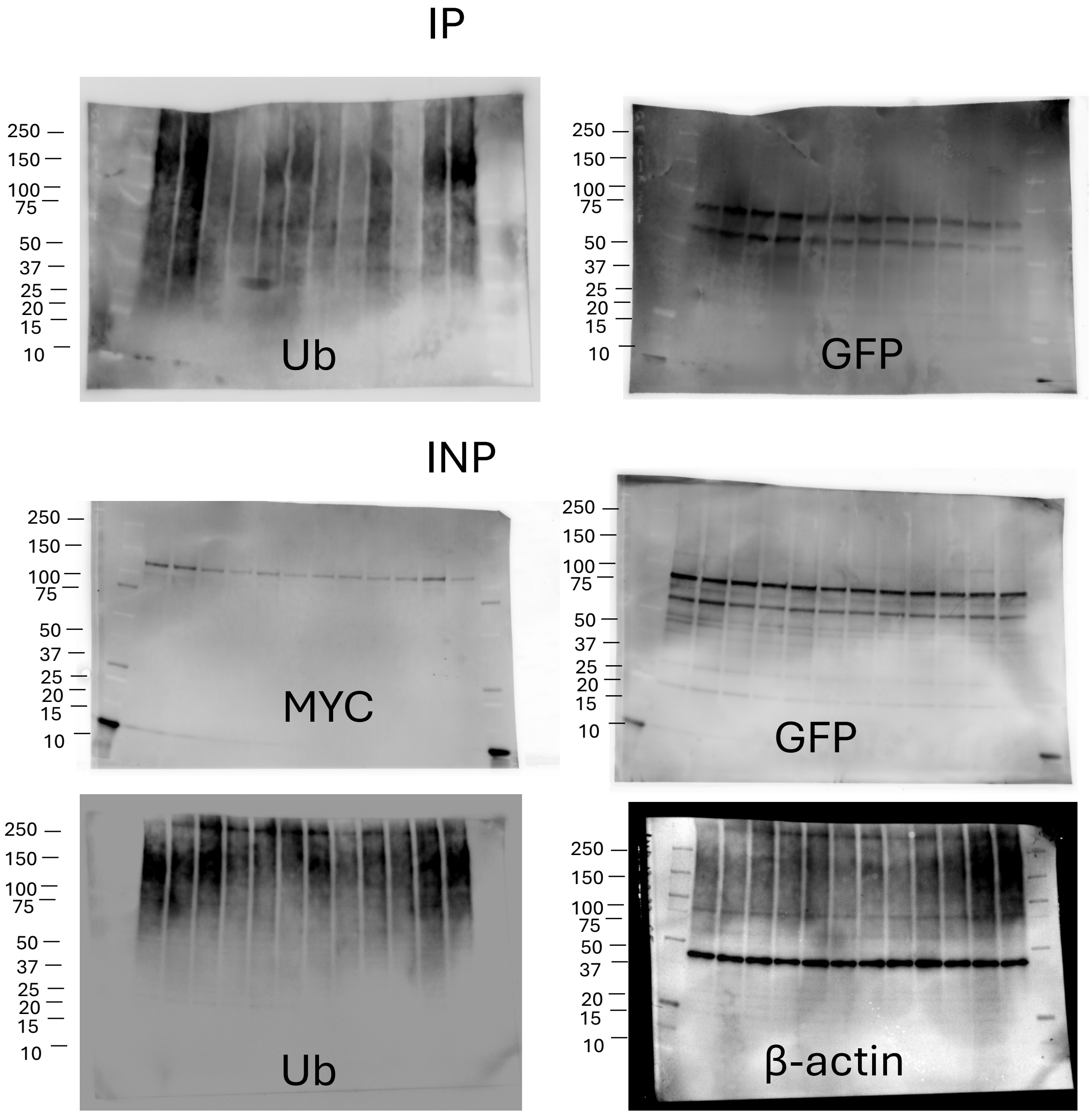

Figure 5C

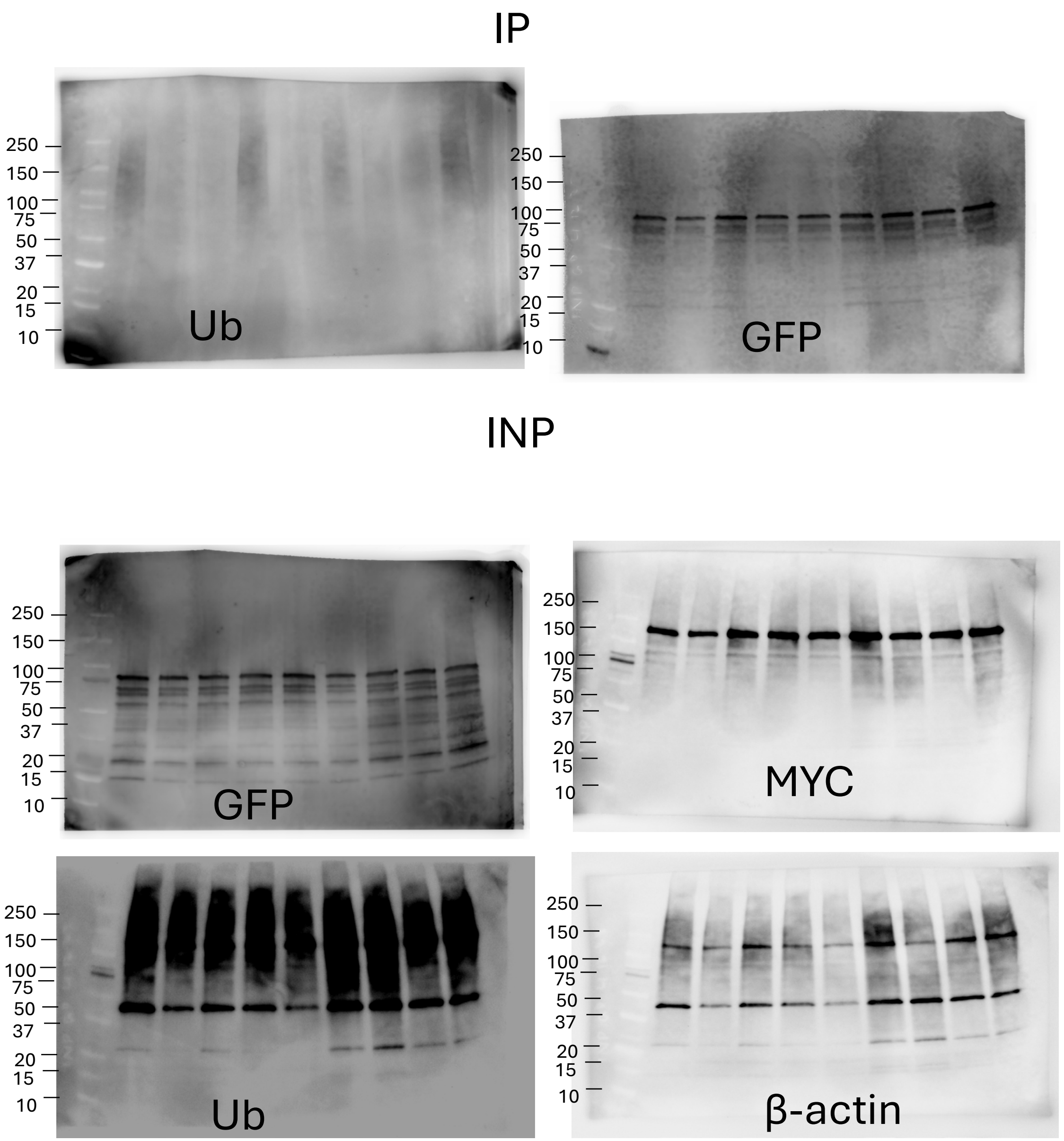

Figure 5D

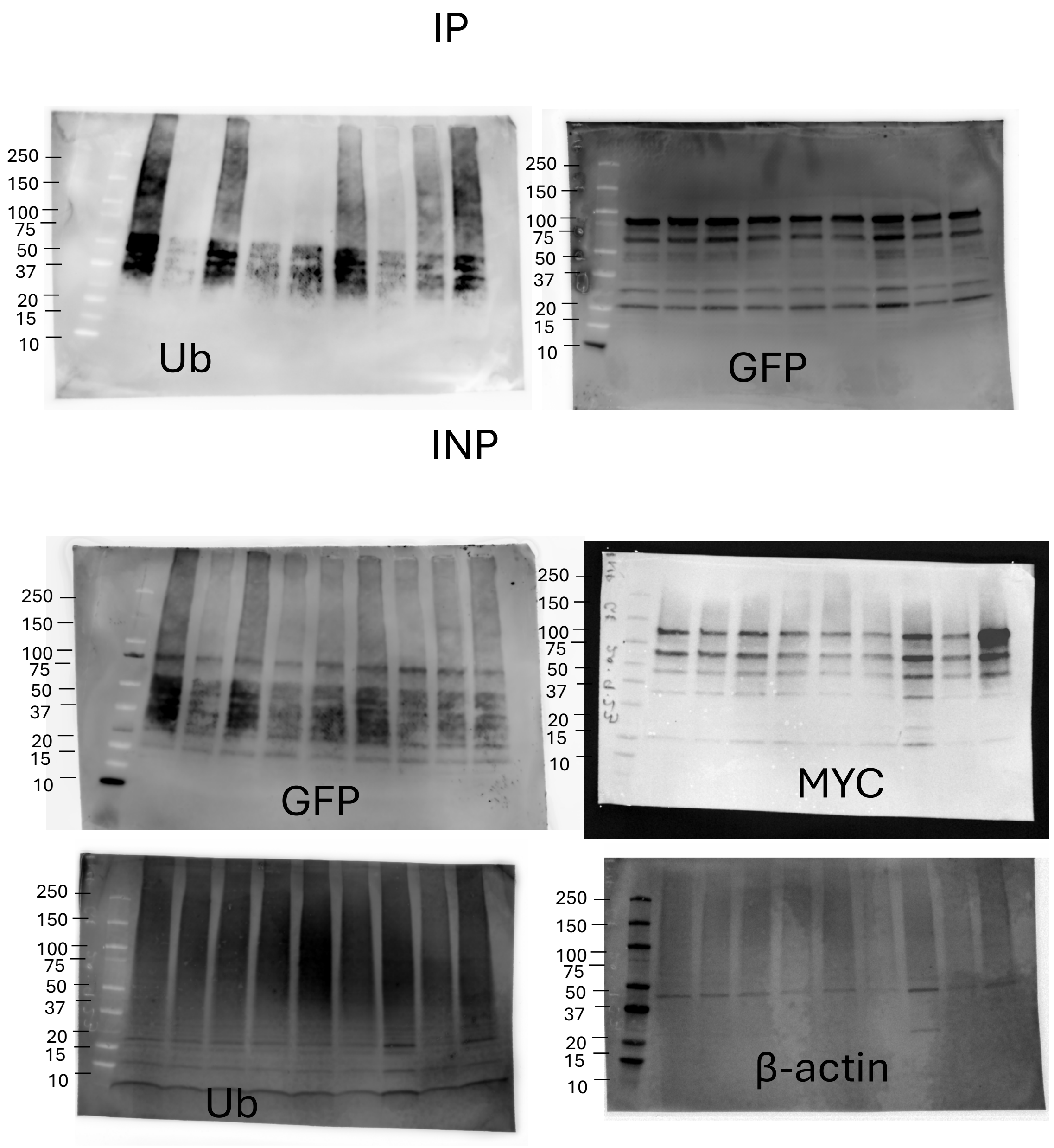

Figure 6D

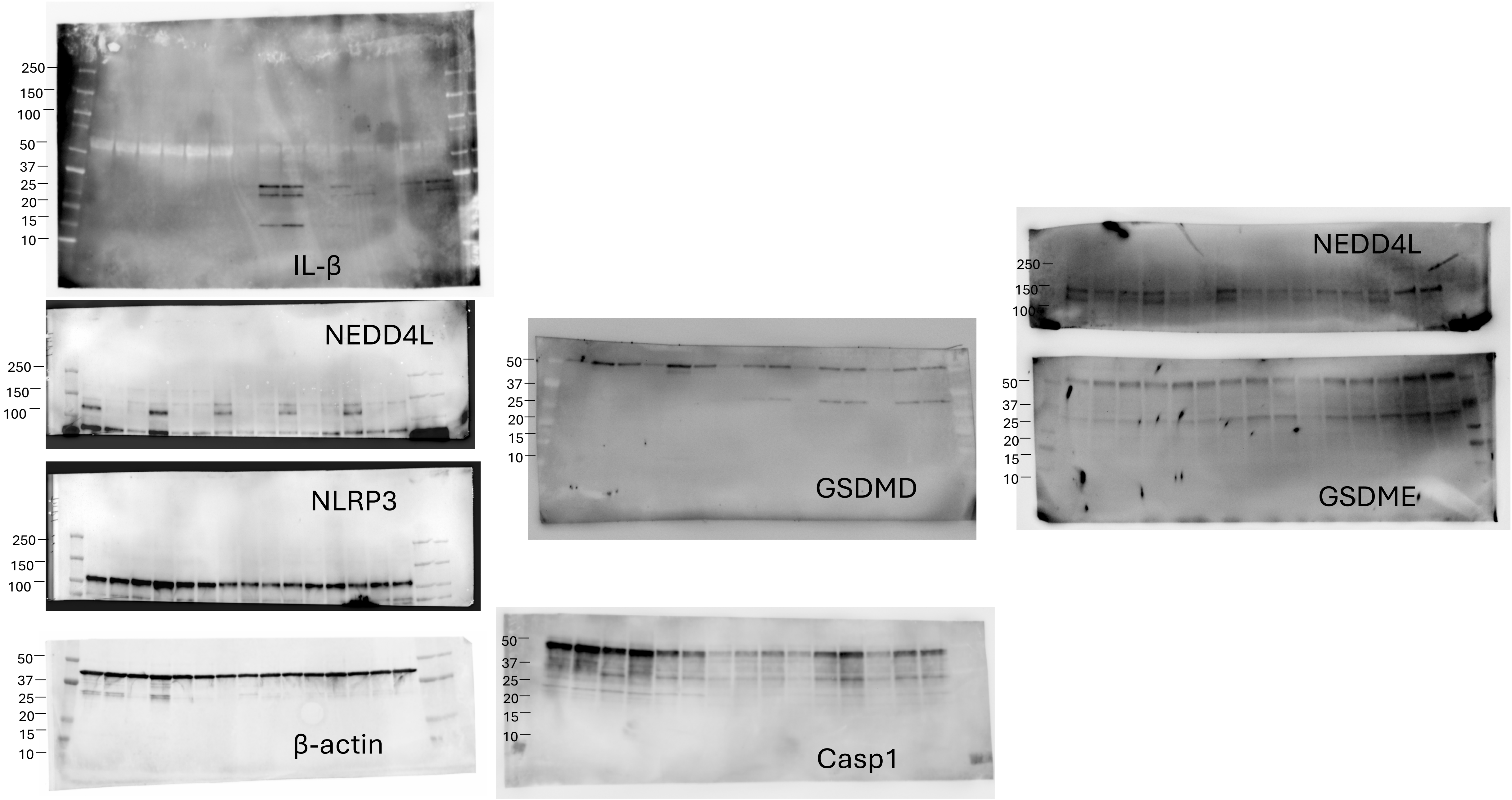

IL-β
NEDD4L
NLRP3
β-actin
GSDMD
Casp1
NEDD4L
GSDME
250
150
100
50
37
25
20
15
10

Figure 6H

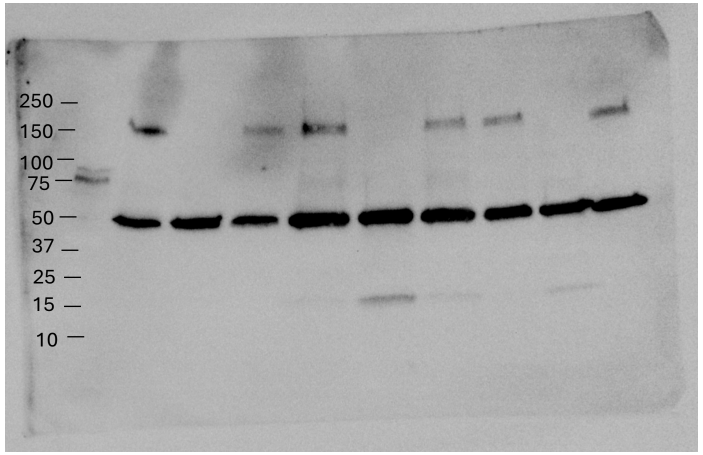

GSDME

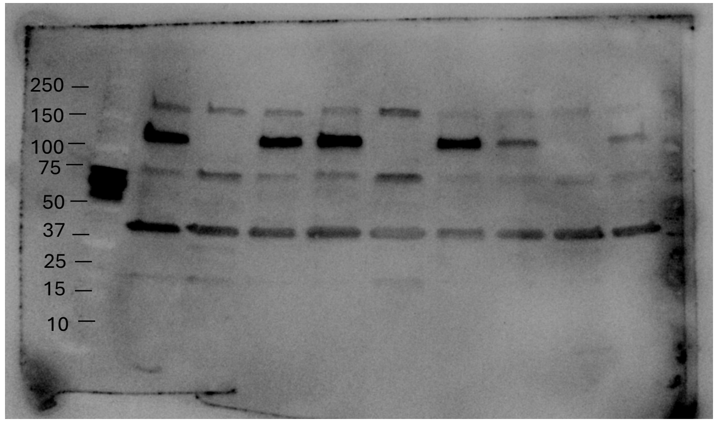

Nedd4-2/β-actin

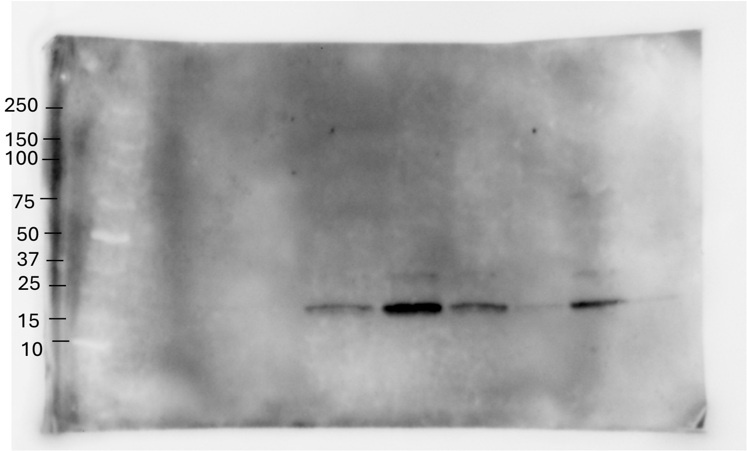

Cl Casp3

Figure 7D

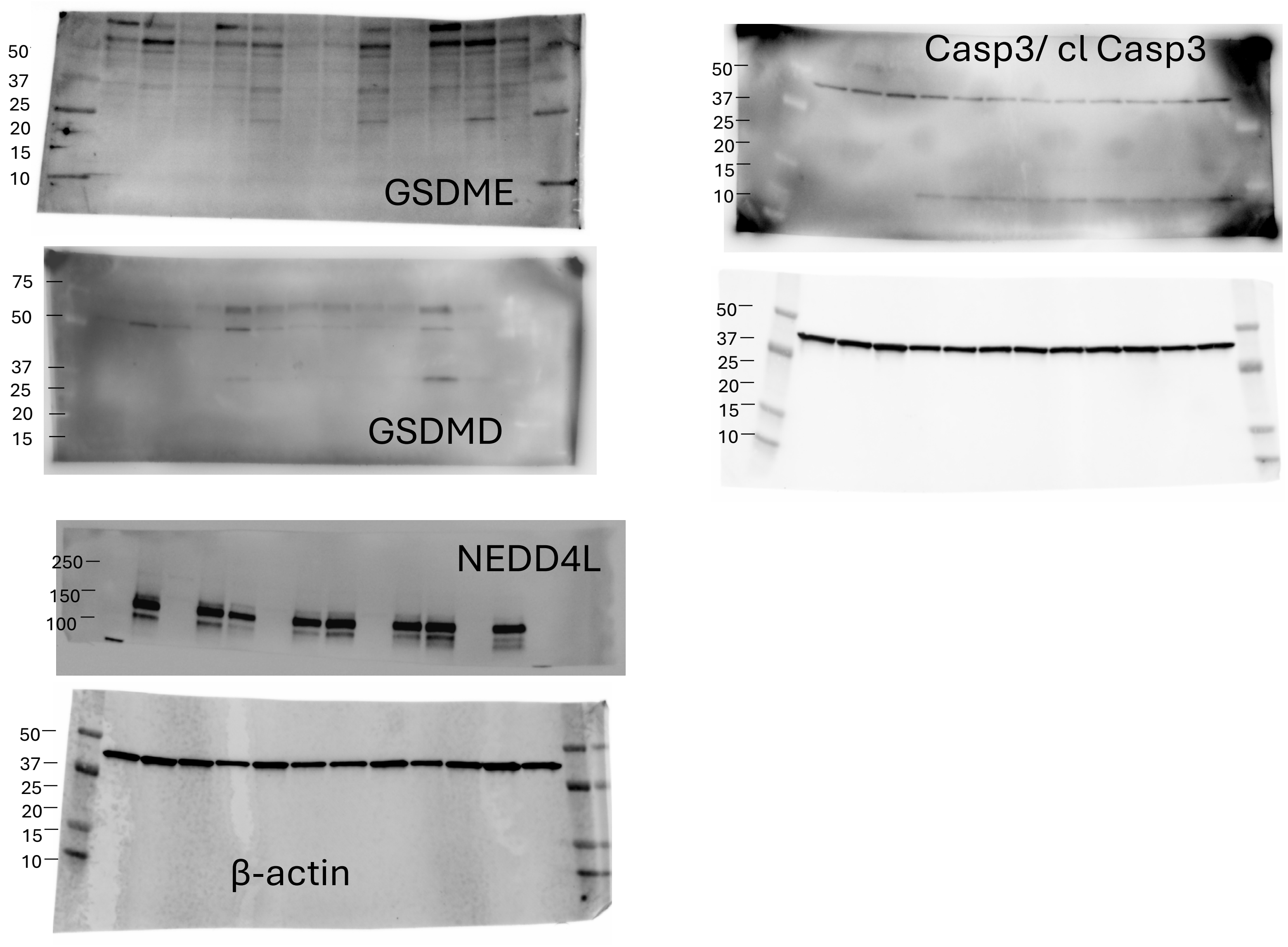

Figure 7E

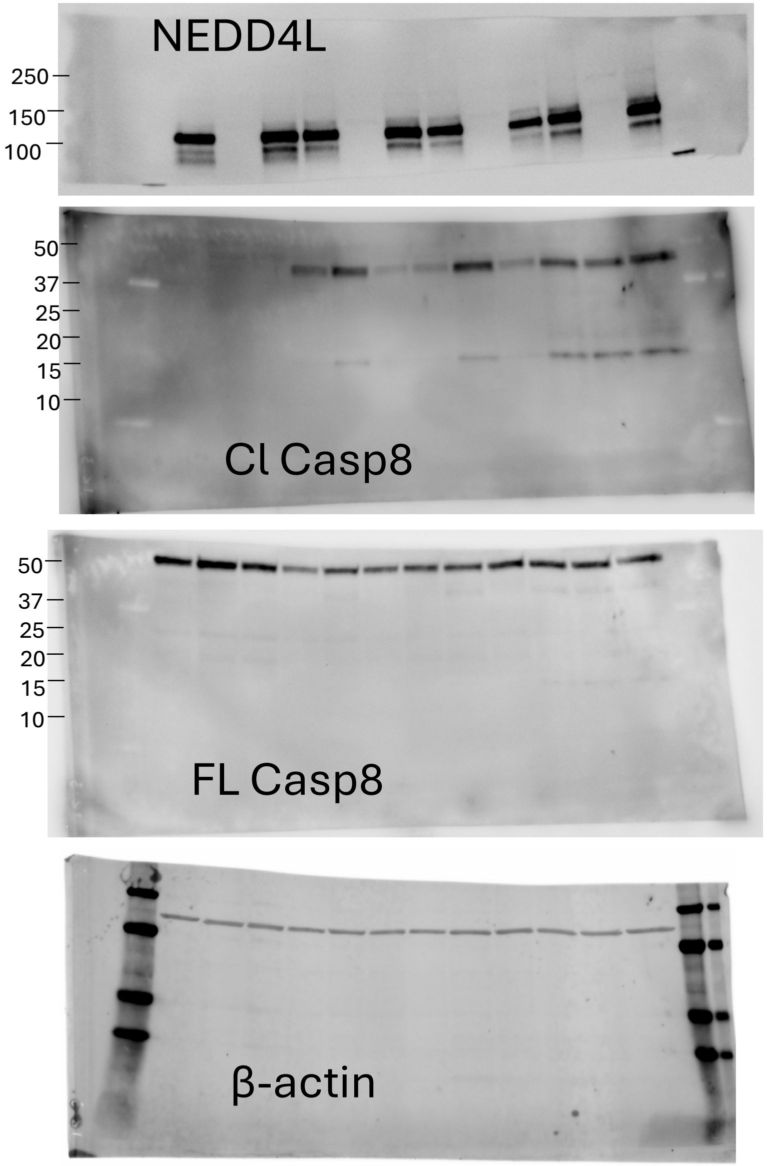

Figure 8B

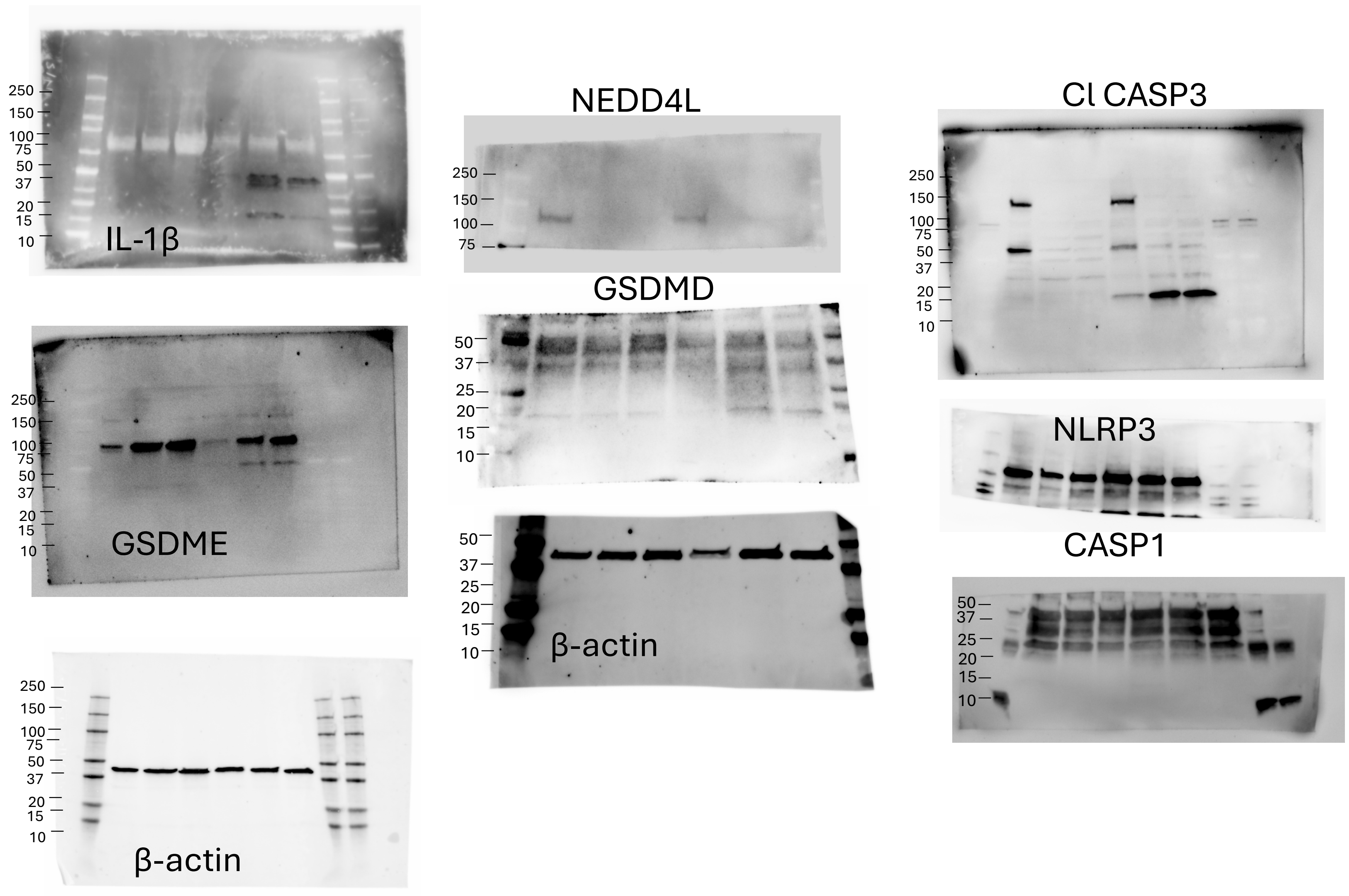

Supplementary figures

Fig. S1E, S1G

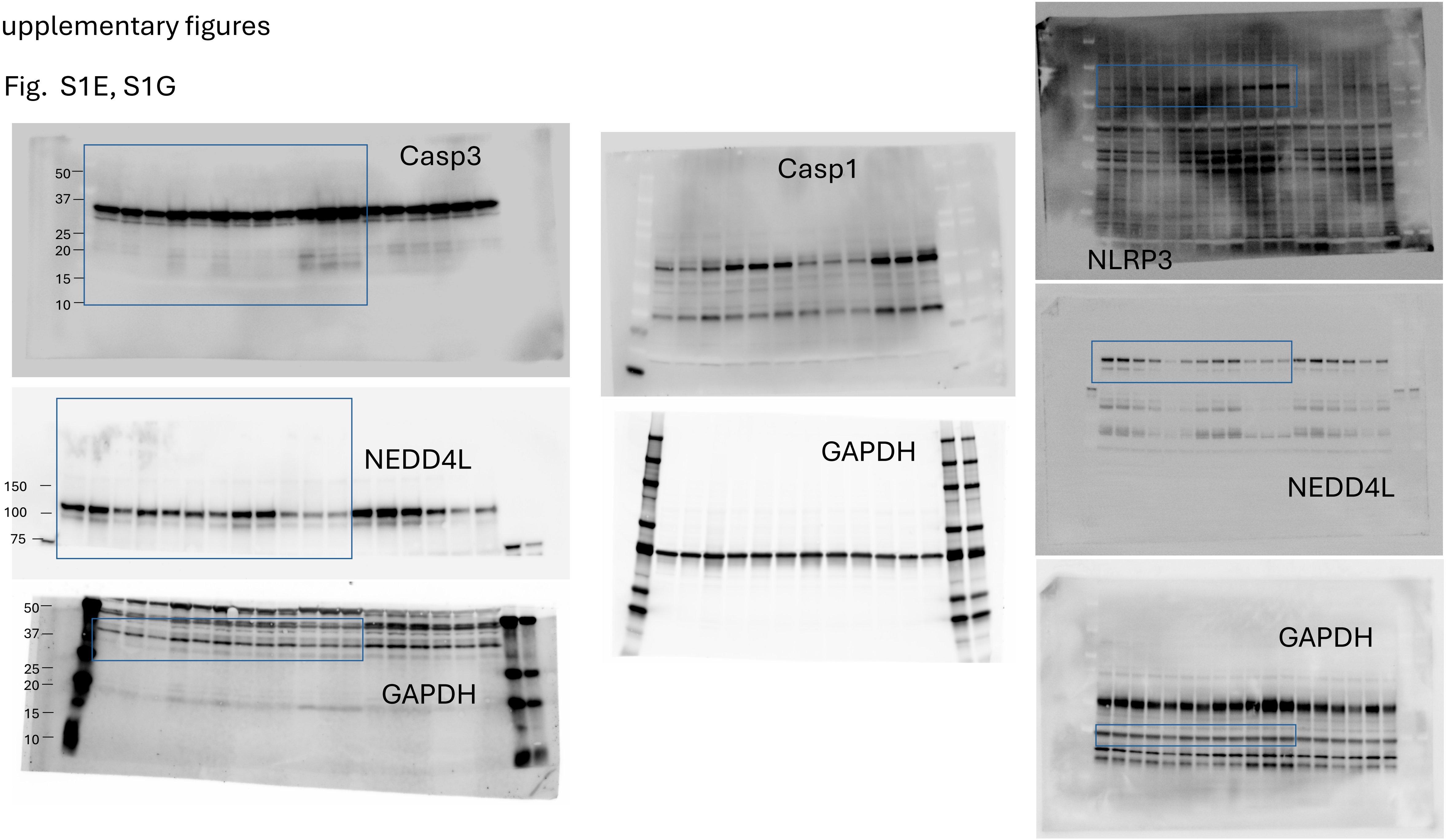

Fig S3A

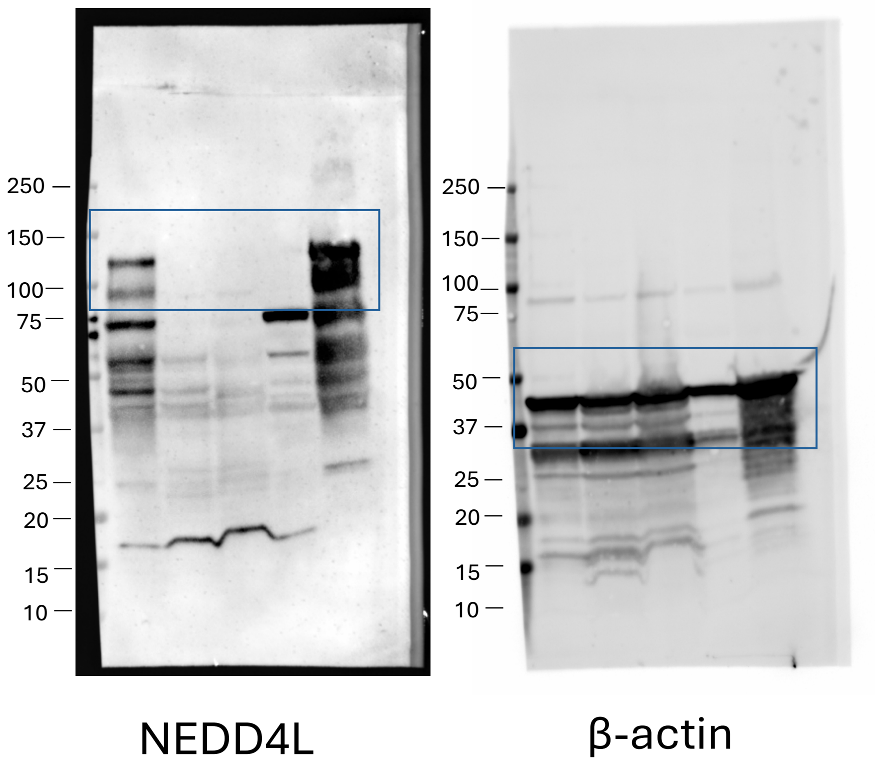
250
150
100
75
50
37
25
20
15
10
NEDD4L
250
150
100
75
50
37
25
20
15
10
β-actin

Fig S3B

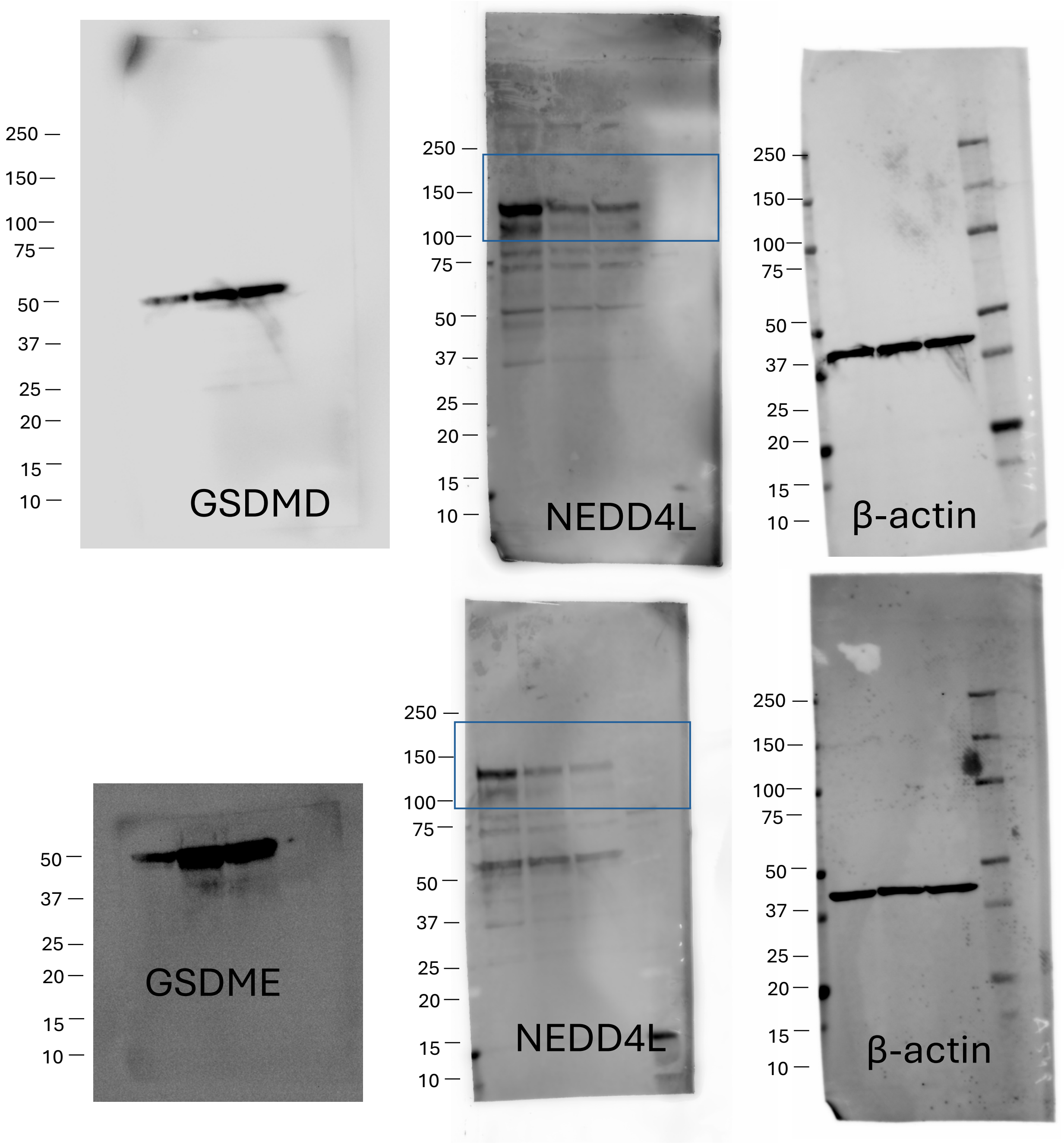

Fig S3C

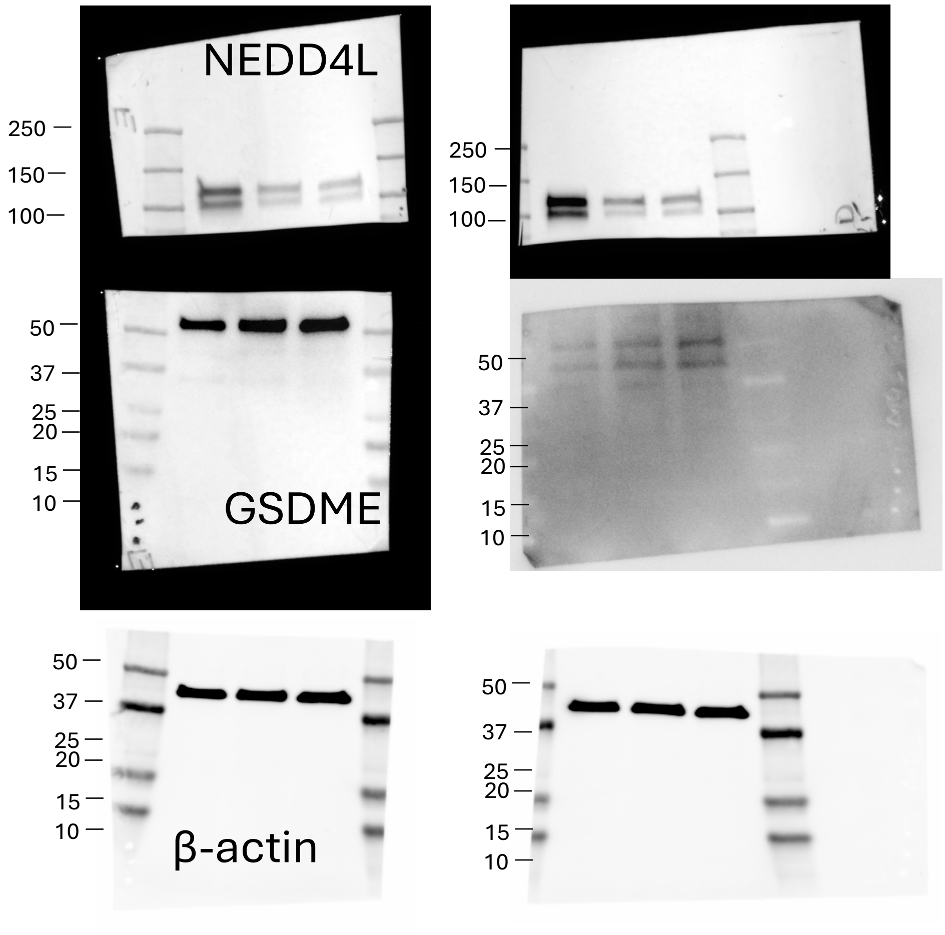

Fig. S4C

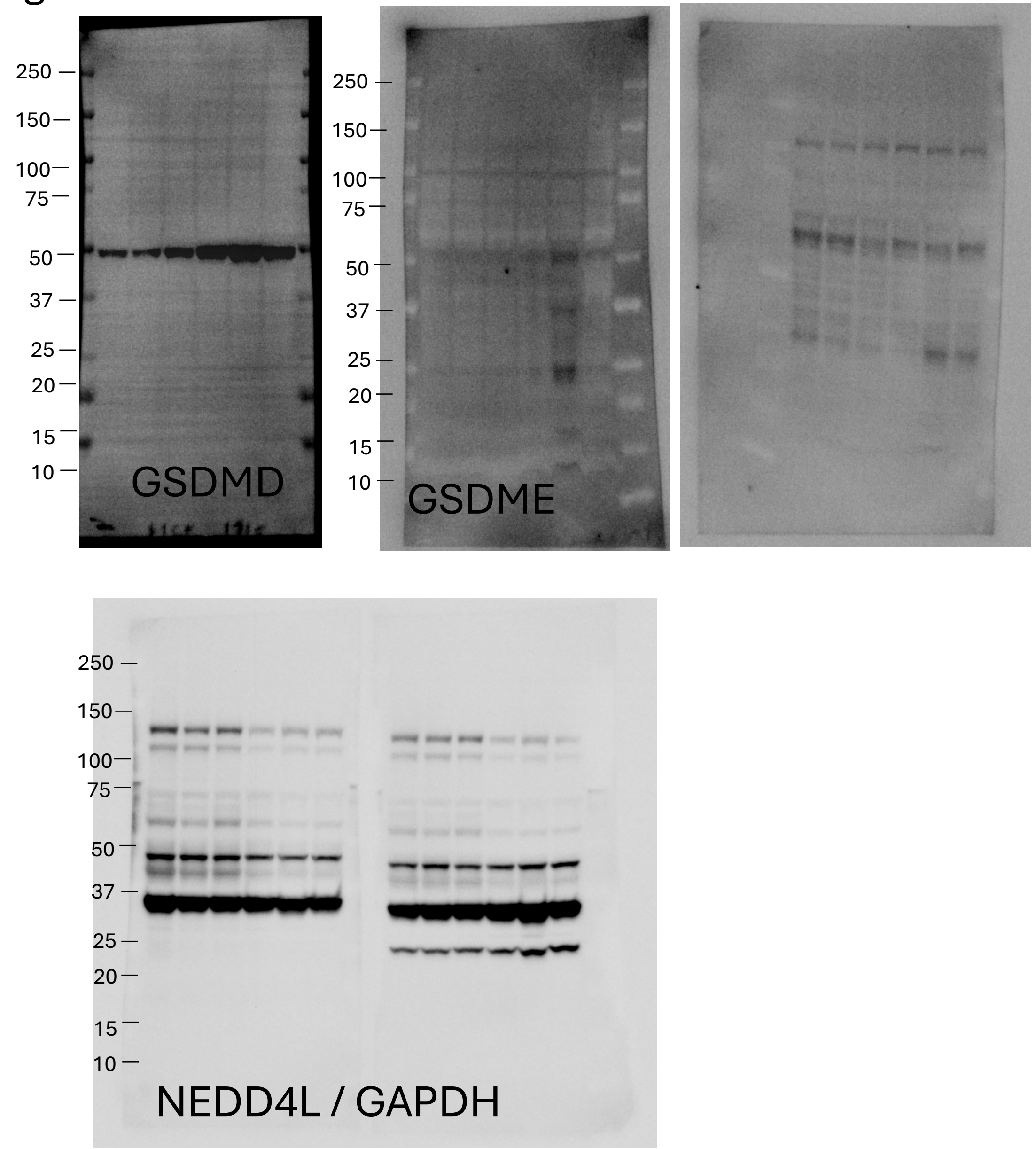

Fig. S4D

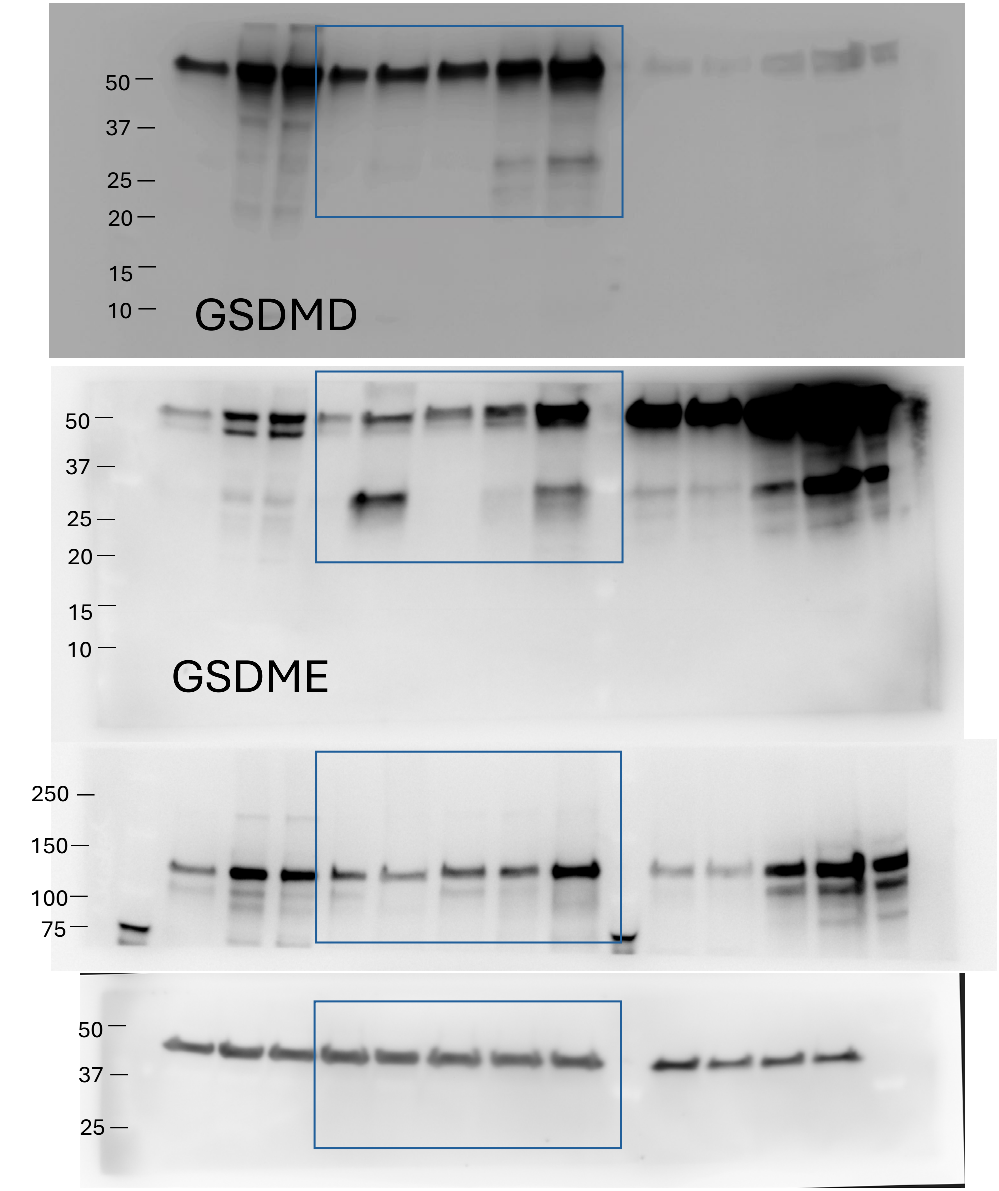

Fig. S5A

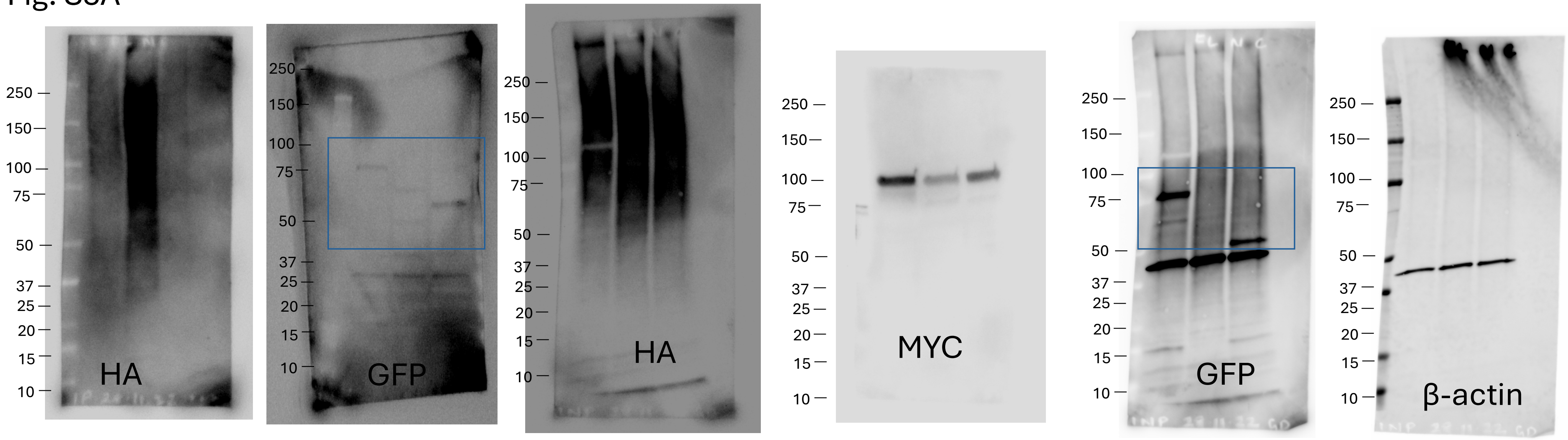

Fig. S5B

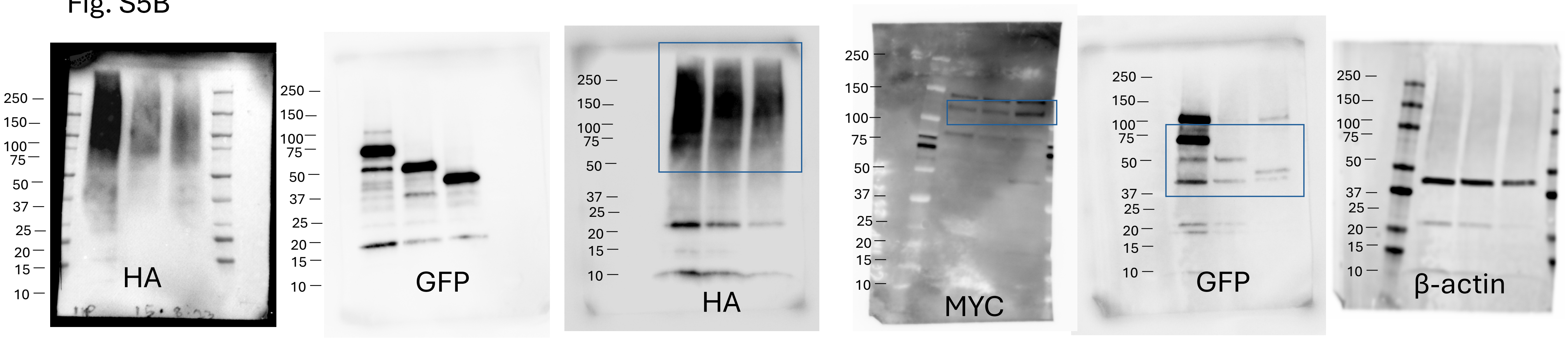

Fig. S6A

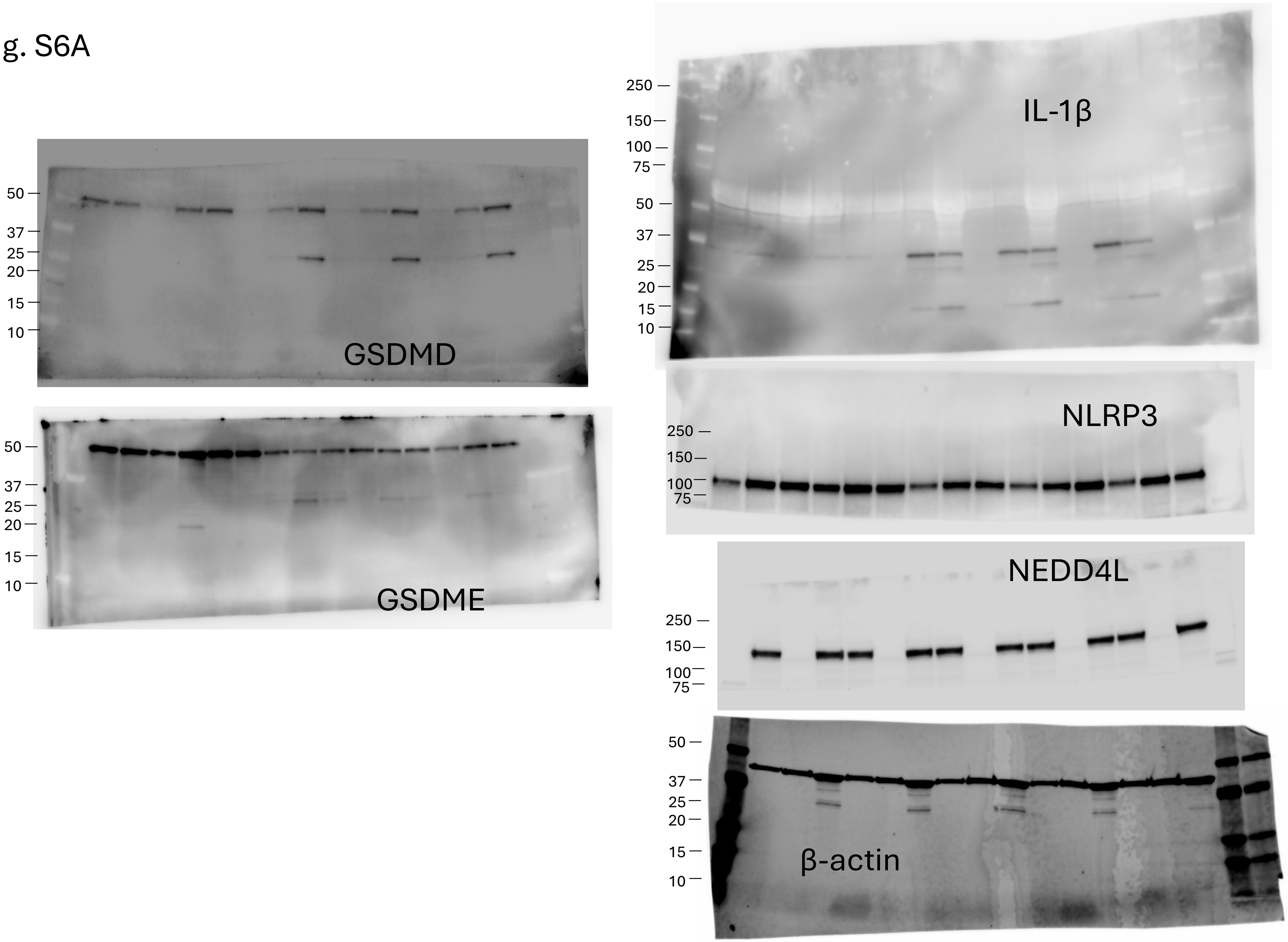
GSDMD
GSDME
IL-1β
NLRP3
NEDD4L
β-actin
50
37
25
20
15
10
250
150
100
75

Fig. S7B

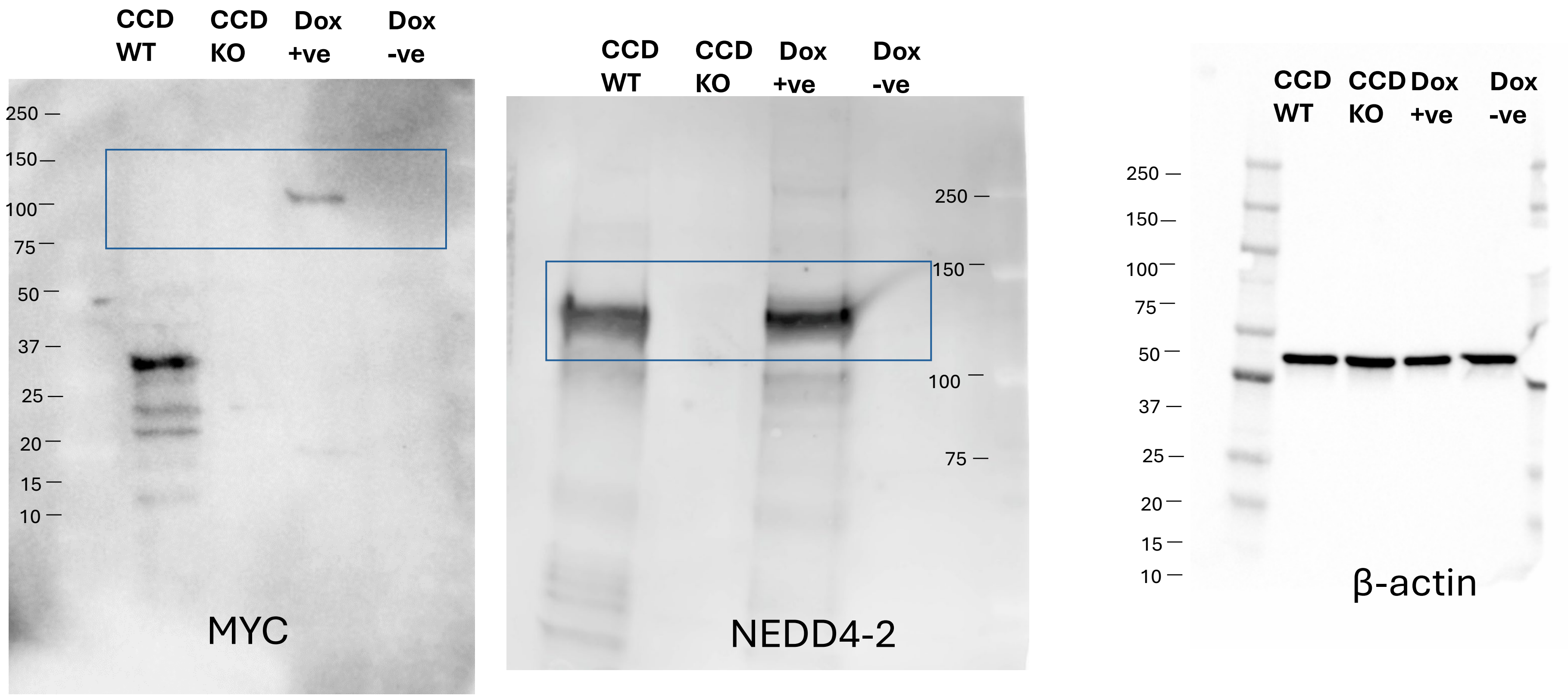
CCD WT
CCD KO
Dox +ve
Dox -ve
250
150
100
75
50
37
25
20
15
10
MYC
CCD WT
CCD KO
Dox +ve
Dox -ve
250
150
100
75
NEDD4-2
CCD WT
CCD KO
Dox +ve
Dox -ve
250
150
100
75
50
37
25
20
15
10
β-actin
